# Delaunay-Like Compact Equilibria in the Liquid Drop Model

**DOI:** 10.1007/s00205-025-02144-6

**Published:** 2025-11-19

**Authors:** Manuel del Pino, Monica Musso, Andres Zuniga

**Affiliations:** 1 Department of Mathematical Sciences, University of Bath, BA2 7AY Bath, UK; 2 Instituto de Ciencias de la Ingeniería, Universidad de O’Higgins (UOH), Avenida Libertador Bernardo O’Higgins 611, Rancagua, Chile

## Abstract

The *liquid drop model* was introduced by Gamow in 1928 and Bohr–Wheeler in 1938 to model atomic nuclei. The model describes the competition between the surface tension, which keeps the nuclei together, and the Coulomb force, corresponding to repulsion among protons. More precisely, the problem consists of finding a surface $\Sigma =\partial \Omega$ in ${\mathbb {R}}^3$ that is critical for the energy

$$\begin{aligned} {\mathcal {E}} (\Omega ) = {{{\textrm{Per}}}\,} (\Omega ) + \frac{1}{2} \int _\Omega \int _\Omega \frac{{\text {d}}x{\text {d}}y}{|x-y|} \end{aligned}$$

under the volume constraint $|\Omega | = m$. The term $\mathrm{Per\,} (\Omega )$ corresponds to the surface area of $\Sigma$. The associated Euler–Lagrange equation is

$$\begin{aligned} H_\Sigma (x) + \int _{\Omega } \frac{{\text {d}}y}{|x-y|} = \lambda \quad \hbox { for all } x\in \Sigma , \quad \end{aligned}$$

where $H_\Sigma$ stands for the mean curvature of the surface, and where $\lambda \in {\mathbb {R}}$ is the Lagrange multiplier associated to the constraint $|\Omega |=m$. Round spheres enclosing balls of volume *m* are always solutions; they are minimizers for sufficiently small *m*. Since the two terms in the energy compete, finding non-minimizing solutions can be challenging. We find a new class of compact, embedded solutions with large volumes, whose geometry resembles a “pearl necklace” with an axis located on a large circle, with a shape close to a Delaunay’s unduloid surface of constant mean curvature. The existence of such equilibria is not at all obvious, since, for the closely related constant mean curvature problem $H_\Sigma = \lambda$, the only compact embedded solutions are spheres, as stated by the classical Alexandrov result.

## Introduction

For open regions $\Omega \subset {\mathbb {R}}^3$, we consider the energy functional

1.1 $$\begin{aligned} {\mathcal {E}} (\Omega ) = \text {Per}(\Omega ) + D(\Omega ), \quad D(\Omega ) = \frac{1}{2} \int _\Omega \int _\Omega \frac{{\text {d}}x\,{\text {d}}y}{|x-y|}, \end{aligned}$$

where $\text {Per}(\Omega )$ denotes the perimeter of $\Omega$, which in the smooth case corresponds to the surface area of its boundary $\partial \Omega$. This energy traces back to Gamow’s liquid drop model [2, 17], a theoretical model used in nuclear physics to describe the structure of atomic nuclei; it treats the nucleus as a drop of incompressible fluid represented by $\Omega$, where nucleons (protons and neutrons) are bound together by strong nuclear forces, explaining phenomena like nuclear fission and nuclear binding energies.

The nucleons are assumed to be distributed with constant density, implying that the number of nucleons is proportional to $|\Omega |$. The perimeter term in the energy (1.1) corresponds to surface tension, which holds the nuclei together. The second term represents the electrostatic Coulomb repulsion among protons.

The basic variational problem in search for equilibria of such system is as follows: Given $m > 0$, we aim to find regions $\Omega \subset {\mathbb {R}}^3$ with smooth boundaries that are critical for the energy ${\mathcal {E}}$ under the volume constraint $|\Omega | = m$. Initially formulated by Bohr and Wheeler [3] to describe the mechanism of nuclear fission, the problem consists in identifying regions $\Omega$ that, for some constant Lagrange multiplier $\lambda$, solve the following equation:

1.2 $$\begin{aligned} H_{\partial \Omega }(x) + \int _\Omega \frac{{\text {d}}y}{|x-y|} = \lambda \quad \quad \text{ for } \text{ all }\quad x\in \partial \Omega . \end{aligned}$$

Here, $H_{\partial \Omega }(x)$ represents the mean curvature operator of the boundary $\partial \Omega$, and the integral term involves the interaction potential between the boundary point *x* and points *y* inside the region $\Omega$.

The first observation is that balls with volume *m* always constitute solutions to this problem. These are critical for both terms in the formula (1.1). Due to the classical isoperimetric inequality, balls minimize perimeter subject to the volume constraint $|\Omega | = m$; see [11] and also [23, Theorem 14.1]. On the other hand, the balls instead maximize the Coulomb interaction term, as shown in [29], and also in the book [21, Theorem 3.7]. The competing nature of the two terms in the energy (1.1) renders problem (1.2) delicate. This problem has been extensively treated in recent years, see [10] for a detailed survey of results until 2017.

Alexandrov’s classical theorem asserts that spheres are the only compact embedded surfaces $\Sigma$ that are critical for perimeter under the enclosed volume constraint, namely, with constant mean curvature, $H_{\Sigma }(x) = \lambda$ for all $x\in \Sigma$. In contrast, Problem (1.2) exhibits richer features.

Let $\Omega$ be a region with $|\Omega |=m$. The scaling $\Omega = m^{1\over 3} E$, such that $|E|=1$, shows that (1.1) becomes

$$\begin{aligned} {{\mathcal {E}}} (\Omega ) = m^{2\over 3} \left( {\textrm{Per}} (E) + m D(E) \right) . \end{aligned}$$

In the small volume regime $m\sim 0$, the above expression indicates that the perimeter term dominates the energy; while the Coulomb interaction energy is dominant in the large mass regime $m>>1$. This suggests that for any mass $m > 0$ sufficiently small, there is a global minimizer, while for large *m*, there are no global minimizers. Indeed, Knupfer–Muratov [20] proved that for any small $m>0$, the energy (1.1) has the ball *B* with $|B|=m$ as a minimizer, see also Julin [18] and Bonacini–Cristoferi [4]. Chodosh–Ruohoniemi [8] have recently shown that is the case for any $m\le 1$. Balls are no longer global minimizers for $m>m_*$, where

$$\begin{aligned} m_*= 5 \frac{(2^{1/3} -1)}{ 1-2^{-2/3}} \approx 3.51. \end{aligned}$$

The value of $m_*$ is precisely the one for which the energy of one ball of mass $m_*$ equals the energy of two balls of half such mass, located at an infinite distance:

$$\begin{aligned} {\mathcal {E}} \left( \left( {m_* \over |B_1|}\right) ^{1/3} \, B_1 \right) = 2\, {\mathcal {E}} \left( \left( {m_* \over 2|B_1|}\right) ^{1/3} \, B_1 \right) . \end{aligned}$$

Frank–Nam [15] proved that for $m\ge 8$ no global minimizes exists, improving a result by Otto–Lu who established this for any sufficiently large *m*. Choksi and Peletier [7] conjectured that global minimizers exist if and only if $m\in (0,m_*]$ and, moreover, they are precisely balls. Frank and Nam [15] proved that for all $0<m\le m_*$ there exists a global minimizer. They also established that these minimizers are balls under the condition that for $m>m_*$ there is no minimizer. Frank–Killip–Nam [14] proved that for $m>8$ there is no minimizer, improving a result by Lu–Otto [22]. The fractional scenario for minimizers was analyzed in [12], while the study of minimizers in the setting of density perimeter was carried out in [1]. See also [13, 26] for results on related models.

It is natural to ask whether for $m>m_*$ there exist solutions to Problem (1.2) other than balls. Very few solutions are known for larger *m*. It turns out that balls are local minimizers well beyond $m_*$: in fact, for any $0<m\le 10$, balls are linearly stable while stability is lost for $m>10$. At $m=10$ a local bifurcation branch in *m* appears which, for $m>10$ yields a local minimizer that is not a ball and, for $m<m_*$, renders a saddle point. The presence of this bifurcation was first detected by Bohr and Wheeler [3] and rigorously established by Frank [16]. As far as the authors are aware, the only known solutions to Problem (1.2), other than balls for very large *m*, are the ones constructed by Ren-Wei: the revolution torii [27], and the double torii [28]. In the periodic case in a higher-dimensional torus, Cristoferi [9] has built highly oscillating local minimizing solutions to the related Ohta–Kawasaki model.

Xu and Du [30] numerically obtained a global description of the bifurcation branches, leading for large masses to toroidal and Delaunay-like shapes, along with providing interesting pictures.

This paper aims to unveil a new class of compact embedded solutions to (1.2) with Delaunay-type shapes. The existence of such equilibria is not at all obvious, since for the closely related constant mean curvature problem $H_\Sigma = \lambda$, the only compact embedded solutions are spheres, as stated by the classical Alexandrov result.

### Delaunay Unduloids

The Delaunay surfaces in the Euclidean 3-dimensional space, denoted as ${\mathbb {R}}^3 = \{(y_1, y_2, y_3) \mid y_j \in {\mathbb {R}}\}$, are Constant Mean Curvature (CMC) surfaces of revolution which are translationally periodic. After a rigid motion and a dilation, we can position its axis of revolution to align with the $y_3$-axis, and its constant mean curvature is set to $H = 2$ (hereafter assumed).

**Fig. 1:**
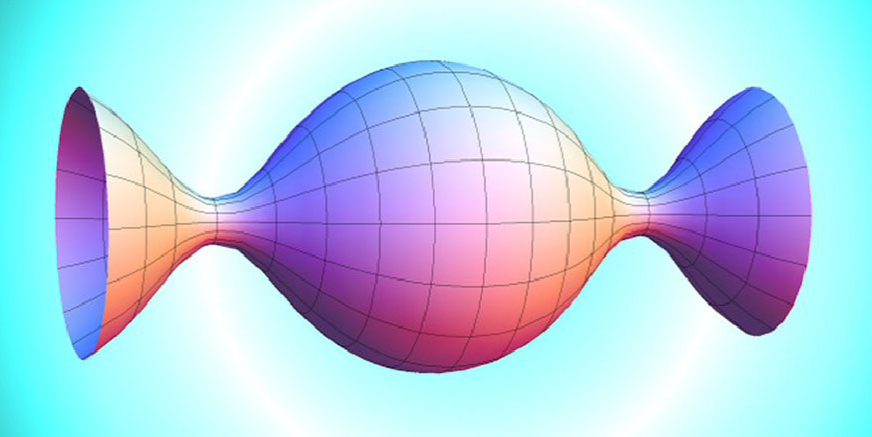
Delaunay surface $$ \Sigma _a$$, $$ 0<a< \frac{1}{2} $$

**Fig. 2:**
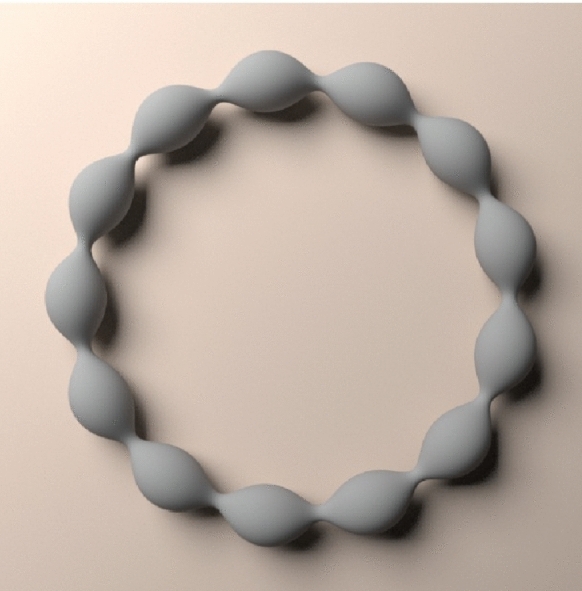
Domain in Theorem 1: $c_a|\log n|^{\frac{1}{3}} \Omega \approx {\tilde{\Omega }}^n$

We examine the profile curve in the half-plane $$ \{(y_1, 0, y_3) \in {\mathbb {R}}^3 \mid y_3 > 0\} $$, which, when rotated about the $$ y_3$$-axis, traces out a Delaunay surface. This curve alternates periodically between maximal and minimal heights concerning the positive $$ y_3 $$ direction. These respective heights are referred to as the bulge radius and the neck radius of the Delaunay surface. Denote the neck radius by $$ a $$. Embedded Delaunay surfaces manifest as a 1-parameter family, known as *unduloids*, which can be parameterized by the neck radius $$ a \in (0, 1/2] $$. For unduloids, $$ a = 1/2 $$ corresponds to the round cylinder. The singular surface as $$ a\rightarrow 0 $$ is a chain of tangent spheres with radii 1 centered along the $$ y_3 $$-axis. Notice that we employ the definition of the mean curvature being the sum of the principal curvatures, rather than their average (Figs. 1 and 2).

We assume that $0<a<{1\over 2}$ is fixed in what follows. The unduloid $\Sigma$ with neck size *a*, also written $\Sigma (a)$, can be parametrized in the form

1.3 $$\begin{aligned} y (\omega ) = (f(y_3)\cos \theta , f(y_3) \sin \theta , y_3), \quad \omega = (\theta ,y_3) \in [0,2\pi )\times {\mathbb {R}}\end{aligned}$$

where $$f (t) = f_a (t) $$ is a positive, smooth function, periodic with period $$T = T_a >0$$, corresponding to the distance between two consecutive necks, its minima, which can also be chosen even, i.e. $$ f(s)= f(-s) $$. The function *f*(*s*) solves the Cauchy problem

1.4 $$\begin{aligned} \left\{ \begin{aligned} - {f'' \over (1+ (f')^2 )^{3\over 2}}&+ {1\over f \sqrt{1+ (f')^2}} = 2\\ f(0) = 1-a,&\quad f'(0)= 0. \end{aligned}\right. \end{aligned}$$

Let us denote by $\Omega$ the volume enclosed by $\Sigma$, namely,

$$\begin{aligned} \Omega \, = \, \big \{\, y \in {\mathbb {R}}^3 \mid |(y_1,y_2)| < f(y_3)\, \big \}. \end{aligned}$$

$\Sigma$ can be decomposed as the superposition of identical portions which we describe as follows: let us define that

1.5 $$\begin{aligned} \Sigma _0= \left\{ \, y \in \Sigma \,\biggr | -{T \over 2} \le y_3 < {T\over 2} \right\} , \end{aligned}$$

and denote that

$$\begin{aligned} \Sigma _k = \Sigma _0 + (0,0, kT) = \left\{ \, y \in \Sigma \,\biggr | -{T \over 2} + kT \le y_3 < {T\over 2} + k T \right\} . \end{aligned}$$

We define similarly $\Omega _k$. Thus,

$$\begin{aligned} \Sigma = \bigcup _{k=-\infty }^\infty \Sigma _k, \quad \Omega = \bigcup _{k=-\infty }^\infty \Omega _k. \end{aligned}$$

The volume of a single block of the Delaunay surface $\Sigma =\Sigma (a)$ will be denoted by

$$\begin{aligned} V= V_a = |\Omega _0|. \end{aligned}$$

To carry out the construction of the critical set, we first consider finite truncations of the sets above, $\Sigma ^n$ and $\Omega ^n$, given by

1.6 $$\begin{aligned} \Sigma ^n:= \bigcup _{k=0}^{n-1} \Sigma _k, \quad \Omega ^n:= \bigcup _{k=0}^{n-1}\Omega _k \quad \text { for } n\in {\mathbb {N}}, \end{aligned}$$

and then we allow for *normal graphs perturbations* of such sets, $\Sigma ^n_h$ and $\Omega ^n_h$, depending on a small smooth function *h*(*y*) defined on $\Sigma ^n$, as

1.7 $$\begin{aligned} \Sigma _h^n:= \{ y + h(y) \nu (y) \, | \, y \in \Sigma ^n \}, \end{aligned}$$

where $\nu (y)$ is the unit normal vector at $y\in \Sigma$, which is explicitly given by

1.8 $$\begin{aligned} \nu (y) = \frac{1}{\sqrt{1+ f' (y_3)^2}} \left( \begin{aligned}&f(y_3)^{-1} { (y_1, y_2) }\\&-f'(y_3) \end{aligned}\right) \end{aligned}$$

and the corresponding enclosed region $\Omega _h^n$ is described by

$$\begin{aligned} \Omega _h^n:= \{ (r y_1, ry_2, y_3) \mid y= (y_1,y_2, y_3) \in \Sigma _h^n, \, r \in [0,1]\, \}. \end{aligned}$$

The construction procedure allow us to work with perturbations $h: \Sigma \rightarrow {\mathbb {R}}$ satisfying the following symmetries:

1.9 $$\begin{aligned} \begin{aligned} h(y_1, y_2, y_3)&= h(-y_1, y_2, y_3 ), \\ h(y_1, y_2, y_3)&= h(y_1, y_2, -y_3 ), \\ h(y_1, y_2, y_3)&= h(y_1, y_2, y_3 +T). \end{aligned} \end{aligned}$$

Let us now consider the change of coordinates *X* given by

1.10 $$\begin{aligned} \begin{aligned} X:{\mathbb {R}}^3\setminus \{0\}&\rightarrow {\mathbb {R}}^3\setminus \{0\},\quad ({\tilde{y}}_1, {\tilde{y}}_2, {\tilde{y}}_3) = X(y_1,y_2,y_3),\\ X(y_1,y_2,y_3)&= \Big ( y_1 \,, \, (R+y_2) \cos \left( \frac{ y_3}{ R} \right) , (R+y_2) \sin \left( \frac{ y_3}{ R} \right) \Big ), \end{aligned} \end{aligned}$$

where $R>0$ is determined by the constraint

1.11 $$\begin{aligned} 2\, \pi \, R= n \, T_a. \end{aligned}$$

The transformation *X* is the composition of a rigid translation of (0, *R*, 0), followed by a rotation in the plane $(y_2,y_3)$ at the angle ${y_3 \over R}$. Finally, we now define the *Delaunay unduloid collar* ${\tilde{\Sigma }}^n:= X(\Sigma ^n)$ and its perturbed version

1.12 $$\begin{aligned} {\tilde{\Sigma }}_h^n:= X \left( \Sigma _h^n \right) ,\end{aligned}$$

and we also introduce the associated solid bounded regions

1.13 $$\begin{aligned} {\tilde{\Omega }}^n = X \left( \Omega ^n \right) , \quad {\tilde{\Omega }}_h^n = X\left( \Omega _h^n \right) . \end{aligned}$$

Under the symmetry assumptions (1.9) on *h*, we have that, for

$$\begin{aligned} {\tilde{y}} \in {\tilde{\Sigma }}_h^n, \quad {\tilde{y}} = X (y), \quad y \in \Sigma _h^n, \end{aligned}$$

it holds that

1.14 $$\begin{aligned} \begin{aligned} X ( y_1,y_2, y_{3} +T)&= {{\mathcal {R}}}_{2\pi \over n} {\tilde{y}} \\ X ( -y_1,y_2, y_{3} )&= {R}_{2,3} {\tilde{y}}, \quad -{T\over 2}< y_3<{T\over 2} \\ X ( y_1,y_2, - y_{3} )&= { R}_{1,2} {\tilde{y}},\quad -{T\over 2}< y_3 <{T\over 2}, \end{aligned} \end{aligned}$$

where ${{\mathcal {R}}}_{\theta }$ is the rotation of angle $\theta$ in the $(y_2,y_3)$-plane, $R_{2,3}$ is the reflection with respect to the plane $y_1=0$, and $R_{1,2}$ is the reflection with respect to the plane $y_3=0$:

$$\begin{aligned} \begin{aligned} {{\mathcal {R}}}_{\theta }&=\left( \begin{matrix} 1 &  0 &  0 \\ 0 &  \cos \theta &  -\sin \theta \\ 0&  \sin \theta &  \cos \theta \end{matrix} \right) , \quad R_{2,3}= \left( \begin{matrix} -1 &  0 &  0 \\ 0 &  1 &  0 \\ 0&  0 &  1\end{matrix} \right) , \quad R_{1,2}= \left( \begin{matrix} 1 &  0 &  0 \\ 0 &  1 &  0 \\ 0&  0 &  -1\end{matrix} \right) . \end{aligned} \end{aligned}$$

### Main Result

In this paper, we prove that in the large mass regime *m*, we can find a collar-like set $\Omega$ that is an equilibrium (critical) for the liquid drop problem, which is a small normal perturbation of a coiled Delaunay surface, of small neck size *a*.

Our main result states that, for any sufficiently small neck size $a>0$ and any large mass $m>>1$, there exists a solution $\Omega$ to the problem (1.2) with $| \Omega |=m$ and such that the scaled domain $c{|\log n|^{\frac{1}{3}} } \Omega$ is a $O( |\log n|^{-1})$-perturbation of the coiled unduloid ${\tilde{\Omega }}^n$ in (1.13). The number *n* is taken close to $[m(\log m - \log (\log m)) C_a]$ for an explicit positive number $C_a$, where $[\cdot ]$ denotes the integer part.

#### Theorem 1

For any sufficiently small neck size $a>0$ there exist (explicit) positive constants $c_a, C_a$ so that, for all sufficiently large $m>1$, there is a domain $\Omega$ with $| \Omega |=m$, solution of Problem (1.2) and $n\in {\mathbb {N}}$ with

1.15 $$\begin{aligned} |[m(\log m - \log (\log m)) C_a] - n| \le 1 \end{aligned}$$

such that the scaling $c_a|\log n|^{\frac{1}{3}}\Omega$ is a $O( |\log n|^{-1})$-normal perturbation of the surface ${\tilde{\Omega }}^n$ in $(1.13)$.

Let us consider a number $\alpha >0$ and replace $\Omega$ with $\alpha {\tilde{\Omega }}$ in equation (1.2) and get the equivalent problem

$$\begin{aligned} \alpha ^{- 1 } H_{{\partial } {\tilde{\Omega }}} ( x) + \int _{ {\tilde{\Omega }} } \frac{ \alpha ^3 {\text {d}}y}{| \alpha x- \alpha y|} = \lambda \quad \text{ for } \text{ all }\quad x\in {\partial } {\tilde{\Omega }}, \end{aligned}$$

so that letting $\gamma = \alpha ^3$ and redefining the Lagrange multiplier $\lambda$, we get that Problem (1.2) with $|\Omega |=m$ is equivalent to finding ${\tilde{\Omega }}$ such that

$$\begin{aligned} \left\{ \begin{aligned} |{\tilde{\Omega }} | =&\gamma ^{-1} m, \\ H_{{\partial } {\tilde{\Omega }}} ( x) \, +&\, \gamma \int _{ {\tilde{\Omega }} } \frac{ {\text {d}}y}{| x- y|}\ =\ \lambda \quad \text{ for } \text{ all }\quad x\in {\partial } {\tilde{\Omega }}. \end{aligned}\right. \end{aligned}$$

To prove Theorem 1 we consider a large number $n\in {\mathbb {N}}$ and then prove that there exists a number $\gamma \approx \frac{c}{\log n}$ with $c>0$ depending of the neck size *a*, and a region ${\tilde{\Omega }}$ satisfying

1.16 $$\begin{aligned} H_{{\partial } {\tilde{\Omega }}} ( x) \, + \, \gamma \int _{ {\tilde{\Omega }} } \frac{ {\text {d}}y}{| x- y|}\ =\ \lambda \quad \text{ for } \text{ all }\quad x\in {\partial } {\tilde{\Omega }} \end{aligned}$$

for a domain ${\tilde{\Omega }}$ which is $O(|\log n|^{-1})$-close to the coiled truncated Delaunay ${\tilde{\Omega }}^n$. This is the content of Theorem 2 below. We will be able to establish this for $a>0$ small or $\frac{1}{4} \le a< \frac{1}{2}$. Using a continuity argument in small $a>0$ we will then be able to establish the relation $|{\tilde{\Omega }} | = \gamma ^{-1} m$ for any large *m* and suitable *n* that satisfies (1.15), so that (1.16) is satisfied. This continuity argument could also be used in the range $\frac{1}{4} \le a < \frac{1}{2}$ under further information on the integral quantity $I_a$ introduced in (2.2) below; see Remark 2.2.

## Scheme of the Proof

Let us recall that we want to solve (1.16), that is,

2.1 $$\begin{aligned} H_{{\tilde{\Sigma }} } ( x) \, + \, \gamma \int _{ {\tilde{\Omega }} } \frac{ {\text {d}}y}{| x- y|}\ =\ \lambda \quad \text{ for } \text{ all }\quad x\in {\tilde{\Sigma }} =\partial {\tilde{\Omega }}. \end{aligned}$$

First, we fix a Delaunay surface $\Sigma = \Sigma (a)$ for a fixed value of the neck size *a*, for which we prove the existence of solutions to Problem (2.1) with ${\tilde{\Sigma }}$ of the form (1.12), namely,

$$\begin{aligned} {\tilde{\Sigma }}:= {{\tilde{\Sigma }}}_h^n = {\partial } {\tilde{\Omega }}_h^n, \end{aligned}$$

provided that $\gamma$ is chosen to fulfill a certain solvability condition. This is the main step of our construction and will occupy most of the paper.

The second step consists of showing that by changing the value of the parameter *a*, we can still obtain a surface solution to (2.1) that is of the form (1.12), having its volume prescribed by any large value *m*. Thus, we fix $0<a< \frac{1}{2}$ and define that

2.2 $$\begin{aligned} I_a:= \int _0^{T/2} \frac{f}{(1+ f'(s)^2)^\frac{5}{2} } \Big [ ff''(2-f'(s)^2) + (1+ 3f'(s)^2) (1+ f'(s)^2) \Big ]\, ds,\nonumber \\ \end{aligned}$$

where $T=T_a$ is the period of the Delaunay surface $\Sigma$ and $f=f_a$ is the profile describing $\Sigma$ as in (1.3), characterized by equation (1.4). We denote by $V_a= |\Omega _0|$ the volume of a single block repeated $T_a$-periodically, to conform the whole of $\Sigma$.

We will establish the following result:

### Theorem 2

Let $a \in (0,{1\over 2})$ be such that

2.3 $$\begin{aligned} I_a>0. \end{aligned}$$

For all sufficiently large $n\in {\mathbb {N}}$, there exist $\gamma _{n,a}, \lambda _{n,a}\in {\mathbb {R}}$, a function $h=h_{n,a}:\Sigma (a) \rightarrow {\mathbb {R}}$, and a compact surface as in (1.12) of the form

$$\begin{aligned} {\tilde{\Sigma }}_h^n = {\partial } {\tilde{\Omega }}_h^n, \end{aligned}$$

which solves Problem (2.1), with

2.4 $$\begin{aligned} |{\tilde{\Omega }}_h^n | = n \, V_a \, \left( 1+ o(1) \right) \quad \text{ as }\quad n\rightarrow \infty . \end{aligned}$$

Moreover, there exists a uniform constant $C>0$ such that

2.5 $$\begin{aligned} \Vert h \Vert _{C^{2, \alpha } (\Sigma )} \le {C \over \log n}, \quad \gamma _{n,a} = {2 I_a \, T_a \over V_a } \, {1\over \log n} \, \left( 1+ o(1) \right) \end{aligned}$$

In (2.4) and (2.5), the limit $o(1) \rightarrow 0$ as $n \rightarrow \infty$ is uniformly in *a*. The function *h* depends continuously on *a*.

### Remark 2.1

The positivity of the integral $I_a$ is a crucial element in our construction. When $a=0$ we have $\frac{T}{2} =1$ and $f(s) = \sqrt{1-s^2}$, so the integral equals 0. When $a=\frac{1}{2}$, the case of the cylinder, we have $f\equiv \frac{1}{2}$ and the integral is trivially positive. We also have $T\rightarrow \pi$ as $a\rightarrow \frac{1}{2}^{-}$. In fact, assumption (2.3) holds for any small neck size *a* and also for $a\ge \frac{1}{4}$. This follows from the Lemma 2.1 below, whose proof we postpone to appendix §7. We conjecture that $I_a>0$ for all $a\in (0,\frac{1}{2})$.

### Lemma 2.1

There exists $a_* \in (0, {1\over 4})$ such that

$$\begin{aligned} I_a >0 \quad \text{ for } \text{ all }\quad a \in (0, a_*] \cup [\tfrac{1}{4}, \tfrac{1}{2}). \end{aligned}$$

In addition, it holds that

2.6 $$\begin{aligned} I_a = 2 a + O(a^2), \quad {\text{ as }} \quad a \rightarrow 0^+. \end{aligned}$$

It is plausible that for a fixed, sufficiently large *n* this result recovers as $a\rightarrow \frac{1}{2}$ the toroidal solution obtained in [27]. Using axial symmetry, they reduce the problem to a two-dimensional setting. We observe that we formally recover their construction. Indeed, for a fixed $a>0$, and a large *n*, we approximately have $m \approx \gamma \, n \, V_a$. On the other hand when $a\rightarrow \frac{1}{2}$ we have $T_a\rightarrow \pi$, $V_a\rightarrow \frac{1}{4} \pi ^2$ and $I_a \rightarrow {\pi \over 4}$. From (2.5) we get, for large *n*,

$$\begin{aligned} \gamma \approx \frac{2I_a T_a}{V_a} \frac{1}{\log n} \approx \frac{2}{\log n}. \end{aligned}$$

Thus $m \approx {\pi ^2 \over 2} {n \over \log n}$. According to (1.11) The large radius of the toroidal solution is

$$\begin{aligned} \gamma ^{\frac{1}{3}}R = \gamma ^{\frac{1}{3}} \frac{nT_a}{2\pi } \approx {2^{1\over 3} \over \pi ^2} \, m \, (\log m )^{2 \over 3}, \end{aligned}$$

while the small radius is $\gamma ^{1\over 3} f\approx {1 \over 2^{2\over 3} (\log m)^{1\over 3} } .$ These expansions coincide with those obtained in [27]. Proving rigorously the connection would require a more precise estimate on the variation of our solutions with the parameter *a* when it is close to the cylinder case $a=\frac{1}{2}$

As in many problems of this type, including the related construction of asymptotically singular CMC surfaces, such as those in [24], the proof of Theorem 2 involves *a Lyapunov–Schmidt type reduction argument*. We now briefly detail this procedure, applied to our problem. For all $\gamma$ suitably small, we perform an expansion of the terms involved in the equation as functions of the perturbation *h*. In Section 3 we expand the mean curvature $H_{{\tilde{\Sigma }}_h^n}$ of the perturbed toroidal Delaunay ${\tilde{\Sigma }}_h^n$, see (1.12). This expansion involves the Jacobi operator of the Delaunay surface under periodic and symmetry conditions. In Section 4 we expand the integral operator in *h* and combine inhomogeneous and smaller order terms in *h* arising from both parts. This leads to solving a nonlinear PDE involving the Jacobi operator of the perturbation *h* and an integral operator of *h*, for which we first solve a projected version. The solution turns out to be a nice function of the parameter $\gamma$. A solution to the full problem is then found by suitably adjusting $\gamma$, that needs to be of size $O(|\log n|^{-1})$. This approach leads us to a perturbation *h* of a comparable size. Finally, a fixed point argument is performed to solve the nonlinear PDE, in which the Jacobi operator is inverted under a zero-mean condition for *h*.

In Sections 5 and 6 we introduce the necessary linear elements and the full fixed point argument. We devote the appendix to some technical results needed in the body of the paper.

### Proof of Theorem 1

The result in Theorem 2 shows that for $n\in {\mathbb {N}}$ large enough there exists a solution to Problem (1.16) for regions $\Omega \subset {\mathbb {R}}^3$ satisfying

$$\begin{aligned}&|\Omega | = \gamma _{n,a}^{-1} m, \quad {\text{ for }} \quad m= \gamma _{n,a} \, |{\tilde{\Omega }}_h^n | = {n \over \log n} \, g_n (a) \\&{\text{ where }} \quad g_n (a) = 2 \, I_a \, T_a \, (1+ o(1)), \end{aligned}$$

and $o(1) \rightarrow 0$ as $n \rightarrow \infty$, uniformly for *a*. The second step in our argument consists in showing that the result remains true for any *m* sufficiently large.

To this purpose, let $m\in {\mathbb {R}}$ be given, large and positive, and take $n \in {\mathbb {N}}$ so that

$$\begin{aligned} {n \over \log n} \, g_n (a)< m < {n +1 \over \log (n+1) } \, g_{n+1} (a) \end{aligned}$$

for some $a\in (0, a_*)$ fixed as in Lemma 2.1. Let $\zeta (x) = \frac{x}{\log x}$. We consider a large number *n* and Taylor expand

$$\begin{aligned} \zeta (n+1) = \zeta (n) + \zeta '(n) + \frac{1}{2} \zeta ''(c), \quad c\in (n,n+1). \end{aligned}$$

Then, we get that

$$\begin{aligned} \frac{n+1}{\log (n+1)} = \frac{n}{\log n} \Big ( 1+ \frac{1}{n} + O( \frac{1}{n\log n}) \Big ) \quad \text{ as }\quad n\rightarrow +\infty . \end{aligned}$$

Hence, we get that

$$\begin{aligned} {n \over \log n} \, g_n (a)< m < {n \over \log n } \, g_{n+1} (a) (1+ o(1)), \quad \end{aligned}$$

with $o(1) \rightarrow 0$ as $n \rightarrow \infty$. From (2.6) in Lemma 2.1 and possibly choosing a smaller $a_*$, we can find $\delta >0$ small (for large *n*) such that

$$\begin{aligned}&a-\delta ,\, \, a+\delta \in (0, a_*),\text { and}\\&{n \over \log n} g_n (a-\delta )< {n \over \log n} \, g_n (a)< m< {n \over \log n } \, g_{n+1} (a) (1+ o(1)) < {n \over \log n} g_n (a+\delta ). \end{aligned}$$

By continuity, there exists $b \in (0, a_*)$ such that $m= {n \over \log n} g_n (b),$ and Theorem 2 guarantees the existence of a solution to (1.16) with $|\Omega |=m$. This concludes the proof of Theorem 1. $\square$

### Remark 2.2

Observe that the result in Theorem 2 is valid for $a\in [{1\over 4}, {1\over 2})$. Moreover, the validity of Theorem 2 only relies upon the condition $I_a >0$. This condition is satisfied in this range of values for *a*. Further information on $I_a$, such as on its derivatives in *a*, are required to establish Theroem 1 for *a* in this range. While we do not address this issue here, we believe that $I_a >0$ and ${d (T_aI_a) \over da} >0$ for all $a \in (0,{1\over 2}).$

## Expansion of the Curvature for ${\tilde{\Sigma }}^n_h$

The purpose of this section is to derive an expression for the mean curvature $H_{{\tilde{\Sigma }}_h^n}$ of the perturbed toroidal Delaunay ${\tilde{\Sigma }}_h^n$, see (1.12) in the Introduction. Before stating our result, we introduce some notation.

The Delaunay surfaces $\Sigma ^n$ mentioned in the introduction are surfaces of revolution, thus a natural parametrization for them is given in cylindrical coordinates. Specifically, if the axis of rotation is vertical, and $y_3$ represents a coordinate along this axis while $\theta$ represents the angular variable around it, then a parametrization of the unperturbed surface $\Sigma ^n$ is

$$\begin{aligned} y (\omega ) = \left[ \begin{matrix} f (y_3) \cos \theta \\ f(y_3) \sin \theta \\ y_3 \quad \end{matrix}\right] , \quad \omega = (\omega _1 , \omega _2) =(\theta , y_3); \end{aligned}$$

see (1.3)–(1.4). The set of parameters $\omega$ is given by

3.1 $$\begin{aligned} \omega \in \Lambda :=\{ (\theta , y_3) \,: \, \theta \in [0,2\pi ), \, y_3 \in [-\tfrac{T}{2}, -\tfrac{T}{2} + n T ] \}, \end{aligned}$$

and the function *f* solves the Cauchy problem (1.4) and it is periodic of period *T*. Tangent vectors to the surface $\Sigma ^n$ are linear combinations of

3.2 $$\begin{aligned} Y_1 (\omega )= D_{\omega _1} y = \left[ \begin{matrix} -f (y_3) \sin \theta \\ f(y_3) \cos \theta \\ 0 \quad \end{matrix}\right] , \quad Y_2 (\omega )= D_{\omega _2} y = \left[ \begin{matrix} f' (y_3) \cos \theta \\ f'(y_3) \sin \theta \\ 1 \quad \end{matrix}\right] , \end{aligned}$$

where $D_{\omega _1} = D_\theta$, $D_{\omega _2} = D_{y_3}$. The unit normal vector $\nu (y)$ to $\Sigma ^n$ at $y \in \Sigma ^n$ as in (1.8) is expressed in the coordinates $\omega$ as

3.3 $$\begin{aligned} \nu (\omega ) = {1\over \sqrt{1+ f' (y_3)^2} } \left[ \begin{matrix} \cos \theta \\ \sin \theta \\ -f' (y_3 ) \quad \end{matrix}\right] , \quad \omega = (\theta , y_3). \end{aligned}$$

The metric on $\Sigma ^n$ is defined by

3.4 $$\begin{aligned} g= Y^T \, Y, \quad {\text{ where }} \quad Y = [Y_1 \, \, Y_2] \end{aligned}$$

and the second fundamental form corresponds to

3.5 $$\begin{aligned} A= - (Y^T \, Y)^{-1} \, Y^T \, D_\omega \nu . \end{aligned}$$

In this section we prove the following result:

### Proposition 3.1

Let $\alpha \in (0,1)$ and $h: \Sigma \rightarrow {\mathbb {R}}$ be a $C^{2,\alpha }$-function satisfying (1.9). For a point $y\in \Sigma ^n$, we write

3.6 $$\begin{aligned} {\tilde{H}}_{{\tilde{\Sigma }}_h^n} ( y ) = H_{{\tilde{\Sigma }}_h^n} \left( {\tilde{y}}_h \right) , \quad {\tilde{y}}_h = X (y_h) \in {\tilde{\Sigma }}_h^n, \quad \end{aligned}$$

where

3.7 $$\begin{aligned} y_h = y + h(y) \nu (y) \in \Sigma _h^n,\quad y \in \Sigma ^n, \end{aligned}$$

and where $\nu$ is given by (1.8). Then, we have the expansion of the curvature

3.8 $$\begin{aligned} \begin{aligned} {\tilde{H}}_{{\tilde{\Sigma }}_h^n} (y ) =&\; 2+ {y_2 \over R \, f} \Biggl ( { (2-(f')^2 ) \, f \, f'' \over (1+ (f')^2 )^{5\over 2}} +{1 + 3 (f')^2 \over (1+ (f')^2 )^{3\over 2}} \Biggl )\\&+ n^{-2} a (y) - J_{\Sigma ^n} [h] + n^{-1} \, \ell [h, D h, D^2 h](y) \\&+ q[h, D h, D^2 h] (y)\quad {\text{ as } } n \rightarrow \infty , \end{aligned} \end{aligned}$$

where *f* solves equation (1.4), *R* in (3.8) is given by (1.11), $a: \Sigma ^n \rightarrow {\mathbb {R}}$ is a smooth function uniformly bounded together with its derivatives as $n \rightarrow \infty$, and it satisfies the symmetries (1.9). Here, $J_{\Sigma ^n}$ denotes the Jacobi operator of $\Sigma ^n$, defined by

$$\begin{aligned} J_{\Sigma ^n}[h] = \Delta _\Sigma h + |A|^2 h, \end{aligned}$$

also expressed in local coordinates as

3.9 $$\begin{aligned} J_{\Sigma ^n } [h] = \frac{1}{ \sqrt{\det g}} {\partial } _j (g^{ij} \sqrt{\det g}\,{\partial } _j h) + |A|^2 h, \end{aligned}$$

where *g* is the metric in (3.4), $g^{ij}:=(g^{-1})_{ij}$, while *A* is the second fundamental form defined in (3.5). In (3.8), $\ell$ is a smooth function, which is uniformly bounded, together with its derivatives, as $n \rightarrow \infty$, and it satisfies the symmetries (1.9). Moreover, $\ell (0,0,0) = 0$, $\nabla \ell (0,0,0) \not = 0$. In (3.8), *q* is a smooth function, uniformly bounded with its derivatives as $n \rightarrow \infty$, and it satisfies the symmetries (1.9). It depends of *h*, $D_\omega h$ and $D_\omega ^2\,h$ with

$$\begin{aligned} q(0,0,0)=0, \quad D q (0,0,0) = 0, \quad D^2 q(0,0,0) \not = 0. \end{aligned}$$

If *h* is also even with respect to $y_2$, then *q* is even in $y_2$.

### Proof

We introduce the parametrization for ${\tilde{\Sigma }}_h^n$ which is induced by one for $\Sigma ^n_h$ via the map *X*, see (1.10).

For a small function *h* on $\Sigma$ we consider the normal graph $\Sigma _h^n$ to the unperturbed Delaunay surface $\Sigma ^n$, given by

$$\begin{aligned} \Sigma _h^n = \{ y+ h(y)\nu (y)\ | \ y\in \Sigma ^n\}. \end{aligned}$$

This surface is naturally parametrized as

$$\begin{aligned} y_h (\omega ) = \left[ \begin{matrix} (f(y_3) + {h (\omega ) \over \sqrt{1+ (f')^2 (y_3) }} ) \cos \theta \\ (f (y_3) + {h (\omega )\over \sqrt{1+ (f')^2 (y_3) }} ) \sin \theta \\ y_3 - {f' (y_3) h (\omega ) \over \sqrt{1+ (f')^2 (y_3) }} \quad \end{matrix}\right] , \quad \omega = (\theta , y_3). \end{aligned}$$

Therefore, a parametrization of the surface ${\tilde{\Sigma }}_h^n = X( \Sigma _h^n )$ is given by ${\tilde{y}}_h = X\circ y_h$ or

$$\begin{aligned} {\tilde{y}}_h (\omega )&= \left[ \begin{matrix} f_h \cos \theta \\ ( R+ f_h \sin \theta ) \, \cos \left( {y_3 - {\bar{h}} \over R}\right) \\ ( R+ f_h \sin \theta ) \, \sin \left( {y_3 - {\bar{h}} \over R}\right) \end{matrix}\right] , \quad \omega = (\theta , y_3) \quad {\text{ where }}\\ f_h (\omega )&=f + {h \over \sqrt{1+ (f')^2 }},\quad {\bar{h}} (\omega ) = {f' h \over \sqrt{1+ (f' )^2} }. \end{aligned}$$

As a first step in our argument, we look for a relation between the mean curvatures of the perturbed toroidal-Delaunay surface ${\tilde{\Sigma }}_h^n$ and the perturbed Delaunay surface $\Sigma _h^n$. $\square$

### Lemma 3.1

Let $\alpha \in (0,1)$ and $h: \Sigma \rightarrow {\mathbb {R}}$ be a $C^{2, \alpha }$ function satisfying (1.9). Then, for $y \in \Sigma ^n$,

3.10 $$\begin{aligned} \begin{aligned} {\tilde{H}}_{{\tilde{\Sigma }}_h^n} (y )&= H_{\Sigma _h^n } (y_h) + {\sin \theta \over R} \Biggl ( { (2-(f')^2 ) \, f \, f'' \over (1+ (f')^2 )^{5\over 2}} +{1 + 3 (f')^2 \over (1+ (f')^2 )^{3\over 2}} \Biggl )\\&\quad + n^{-2} \, a(y) + n^{-1} \, \ell [h, D h, D^2 h] \quad {\text{ as } } n \rightarrow \infty , \end{aligned} \end{aligned}$$

where ${\tilde{H}}_{{\tilde{\Sigma }}_h^n}$ as in (3.6) and

$$\begin{aligned} y_h = y + h(y) \nu (y) \end{aligned}$$

as in (3.7). Here *a*(*y*) and $\ell$ stand for smooth functions of *y*, which are uniformly bounded with their derivatives, as $n \rightarrow \infty$, and they satisfy the symmetries (1.9). Besides, $\ell$ depends on *h* and its derivatives, with $\ell (0,0,0) = 0$ and $\nabla \ell (0,0,0) \not = 0$.

The second step in our argument to prove Proposition 3.1 consists in relating the mean curvatures of the perturbed Delaunay surface $\Sigma _h^n$ and the un-perturbed Delaunay surface $\Sigma ^n$.

### Lemma 3.2

Let $\alpha \in (0,1)$ and $h: \Sigma ^n \rightarrow {\mathbb {R}}$ be a $C^{2, \alpha }$ function satisfying (1.9). Then, for $y \in \Sigma ^n$,

$$\begin{aligned} H_{\Sigma ^n_h} (y_h) = H_{\Sigma ^n} (y) - J_{\Sigma ^n} [h] + q[h, D_\omega h, D_\omega ^2 h], \end{aligned}$$

where

$$\begin{aligned} y_h = y + h(y) \nu (y), \end{aligned}$$

as in (3.7), and $J_{\Sigma ^n}$ is the Jacobi operator defined in (3.9). The function *q* is a smooth function of *y*, uniformly bounded with its derivatives as $n \rightarrow \infty$, and it satisfies the symmetries (1.9). If *h* is also even with respect to $y_2$, then *q* is even in $y_2$. It depends of *h*, $D_\omega h$ and $D_\omega ^2\,h$ with

$$\begin{aligned} q(0,0,0)=0, \quad D q (0,0,0) = 0, \quad D^2 q(0,0,0) \not = 0. \end{aligned}$$

The proof of Proposition 3.1 readily follows from Lemmas 3.1 and 3.2, and the fact that $H_{\Sigma ^n} (y)=2.$ $\square$

### Remark 3.1

For $h: \Sigma ^n \rightarrow {\mathbb {R}}$ satisfying (1.9), the function

$$\begin{aligned} y \rightarrow H_{\Sigma _h^n} (y_h), \quad y_h = y + h(y) \nu (y) \end{aligned}$$

satisfies the same symmetries (1.9). Besides, if *h* is even with respect to $y_2$, then $H_{\Sigma _h^n} (y_h)$ is even in $y_2$. Also, the function

$$\begin{aligned} y \rightarrow {\tilde{H}}_{{\tilde{\Sigma }}_h^n} (y), \end{aligned}$$

defined in (3.6), enjoys (1.9): it is even in $y_1$, $y_3$ and periodic of period *T* in $y_3$. This is consequence of the properties (1.14) of the map *X*.

The rest of the section is devoted to prove Lemmas 3.1 and 3.2.

### Proof of Lemma 3.1

Let ${\tilde{Y}}_h$ and $Y_h$ be the $3 \times 2$ matrices given by

3.11 $$\begin{aligned} {\tilde{Y}}_h (\omega ) = [ {\tilde{Y}}_{h1} \quad {\tilde{Y}}_{h2} ], \quad Y_h (\omega ) = [ Y_{h1} \quad Y_{h2} ], \end{aligned}$$

where

3.12 $$\begin{aligned} {\tilde{Y}}_{hj} = D_{\omega _j} {\tilde{y}}_h (\omega ), \quad Y_{hj} = D_{\omega _j} y_h (\omega ), \end{aligned}$$

for $j=1,2$. The Riemannian metrics on ${\tilde{\Sigma }}_h^n$ and $\Sigma _h^n$ are respectively given by the $2 \times 2$ matrices

3.13 $$\begin{aligned} {\tilde{g}}_h = {\tilde{Y}}_h^T \, {\tilde{Y}}_h, \quad g_h = Y_h^T \, Y_h, \end{aligned}$$

where the symbol $^T$ stands for taking the transposed matrix. Let $\nu _h$ be the unit normal to $\Sigma _h^n$

$$\begin{aligned} \nu _h (\omega )= {Y_{h1} \times Y_{h2} \over \Vert Y_{h1} \times Y_{h2} \Vert } \end{aligned}$$

and ${\tilde{\nu }}_h (\omega )$ be the unit normal vector to ${\tilde{\Sigma }}_h^n$,

$$\begin{aligned} \begin{aligned} {\tilde{\nu }}_h (\omega )&= {{\tilde{Y}}_{h1} \times {\tilde{Y}}_{h2} \over \Vert {\tilde{Y}}_{h1} \times {\tilde{Y}}_{h2} \Vert }, \quad {\text{ where }} \quad {\tilde{Y}}_{hj} = D_{\omega _j} {\tilde{y}}_h = D_j \left( X \circ y_h \right) , \quad j=1,2. \end{aligned} \end{aligned}$$

We also introduce the $3 \times 2$ matrices for ${\tilde{\Sigma }}^n_h$ and $\Sigma _h^n$ given, respectively, by

3.14 $$\begin{aligned} {\tilde{B}}_h = [ D_{\omega _1} {\tilde{\nu }}_h \quad D_{\omega _2} {\tilde{\nu }}_h ] \quad B_h = [ D_{\omega _1} \nu _h \quad D_{\omega _2} \nu _h ]. \end{aligned}$$

The mean curvatures of ${\tilde{\Sigma }}^n_h$ and $\Sigma ^n_h$ can be computed using the following formulas (see for instance [25]):

3.15 $$\begin{aligned} \begin{aligned} H_{{\tilde{\Sigma }}_h^n}&= \, {d \over dz} \log \left( \sqrt{ \det {\tilde{g}}_h (z)} \right) _{|_{z=0}} \\ H_{ \Sigma _h^n}&=\, {d \over dz} \log \left( \sqrt{ \det g_h (z)} \right) _{|_{z=0}}. \end{aligned} \end{aligned}$$

Here, for any $z \in {\mathbb {R}}$,

$$\begin{aligned} {\tilde{g}}_h (z)&= [ {\tilde{Y}}_h + z {\tilde{B}}_h ]^T [ {\tilde{Y}}_h + z {\tilde{B}}_h ] , \\ g_h(z)&= [Y_h + z B_h ]^T [Y_h + z B_h ]. \end{aligned}$$

Let ${\tilde{A}}_h$ and $A_h$ be the second fundamental forms of ${\tilde{\Sigma }}_h^n$ and $\Sigma _n^h$, respectively,

$$\begin{aligned} {\tilde{A}}_h = - \left( {\tilde{Y}}_h^T \, {\tilde{Y}}_h \right) ^{-1} \, {\tilde{Y}}_h^T \, D_\omega {\tilde{\nu }}_h, \quad A_h = - \left( Y_h^T \, Y_h \right) ^{-1} \, Y_h^T \, D_\omega \nu _h. \end{aligned}$$

We claim that

3.16 $$\begin{aligned} \begin{aligned} {\tilde{g}}_h (z)&= {\tilde{Y}}_h^T \, {\tilde{Y}}_h \, \left( I - z {\tilde{A}}_h \right) ^2 = {\tilde{g}}_h \, \left( I - z {\tilde{A}}_h \right) ^2 \\ g_h (z)&= Y_h^T \, Y_h \, \left( I - z A_h \right) ^2 = g_h \, \left( I - z A_h \right) ^2. \end{aligned} \end{aligned}$$

The proof of the first formula in (3.16) follows from the following geometric considerations: for any $z \in {\mathbb {R}}$ a straightforward computation gives that

$$\begin{aligned} {\tilde{g}}_h(z)&= {\tilde{Y}}_h^T \, {\tilde{Y}}_h + z \left( {\tilde{Y}}_h^T \, D_\omega {\tilde{\nu }}_h + D_\omega {\tilde{\nu }}_h^T \, {\tilde{Y}}_h \right) + z^2 D_\omega {\tilde{\nu }}_h^T \, D_\omega {\tilde{\nu }}_h . \end{aligned}$$

By definition of ${\tilde{\nu }}_h$ we have that ${\tilde{Y}}_{hi}^T \, {\tilde{\nu }}_h = {\tilde{\nu }}_h \cdot {\tilde{Y}}_{hi} =0$ for $i=1,2$ (see (3.12)). Differentiating this expression by $\omega _j$ and using the fact that $D_{\omega _i } {\tilde{Y}}_{hj} = D_{\omega _j } {\tilde{Y}}_{hi}$, we obtain that $D_{\omega _i} {\tilde{\nu }}_h \cdot {\tilde{Y}}_{hj} = D_{\omega _j} {\tilde{\nu }}_h \cdot {\tilde{Y}}_{hi}$. Hence, for $i, j=1,2$,

$$\begin{aligned} \left( {\tilde{Y}}_h^T \, D_\omega {\tilde{\nu }}_h \right) _{ij} = \tilde{Y}_{hi}^T \, D_{\omega _j} {\tilde{\nu }}_h = D_{\omega _j} {\tilde{\nu }}_h^T \, {\tilde{Y}}_{hi} = \left( D_\omega ^T{\tilde{\nu }}_h \, \, {\tilde{Y}}_h \right) _{ij}; \end{aligned}$$

that is,

$$\begin{aligned} {\tilde{Y}}_h^T \, D_\omega {\tilde{\nu }}_h = D_\omega ^T {\tilde{\nu }}_h \, {\tilde{Y}}_h. \end{aligned}$$

We use that $D_\omega {\tilde{\nu }}_h^T = D_\omega ^T {\tilde{\nu }}_h$. Besides, the vectors $D_{\omega _i} {\tilde{\nu }}_h$, $i=1,2$, belong to the tangent space to ${\tilde{\Sigma }}_h^n$ which is spanned by ${\tilde{Y}}_{h1}$ and ${\tilde{Y}}_{h2}$. Hence $D_\omega {\tilde{\nu }}_h$ coincides with its projection on that space, that is,

$$\begin{aligned} D_\omega {\tilde{\nu }}_h= {\tilde{Y}}_h \left( {\tilde{Y}}_h^T \, \tilde{Y}_h \right) ^{-1} \, {\tilde{Y}}_h^T \, D_\omega {\tilde{\nu }}_h. \end{aligned}$$

Using the above facts, we obtain the first equality in (3.16). The second equality can be derived in the same manner.

Formula (3.16) readily gives that

$$\begin{aligned} \det {\tilde{g}}_h (z)&= \det {\tilde{g}}_h \left( 1 - z \, {\textrm{trace }} ({\tilde{A}}_h ) + z^2 \det {\tilde{A}}_h \right) ^2 \end{aligned}$$

and hence, from (3.15),

$$\begin{aligned} H_{{\tilde{\Sigma }}_h^n}&=-\, {\textrm{trace}} ({\tilde{A}}_h) \quad {\text{ and }} \quad H_{\Sigma _h^n} =-\, {\textrm{trace}} ( A_h). \end{aligned}$$

Thus the relation between the mean curvatures of ${\tilde{\Sigma }}^n_h$ and $\Sigma ^n_h$ follows from comparing the trace of the matrix ${\tilde{A}}_h = - \left( {\tilde{Y}}_h^T \, {\tilde{Y}}_h \right) ^{-1} \, {\tilde{Y}}_h^T \, D_\omega {\tilde{\nu }}_h$ with the trace of the matrix $A_h = - \left( Y_h^T \, Y_h \right) ^{-1} \, Y_h^T \, D_\omega \nu _h$.

We recall that the parametrization $${\tilde{y}}_h$$ of $${\tilde{\Sigma }}^n_h$$ is given in terms of the parametrization $$ y_h$$ of $$ \Sigma ^n_h$$ through the map *X* defined in (1.10), that is $${\tilde{y}}_h (\omega ) = ( X \circ y_h ) \, (\omega )$$. Hence,

$$\begin{aligned} {\tilde{Y}}_h = DX \, Y_h , \quad {\text{ and }} \quad {\tilde{Y}}_h^T \, {\tilde{Y}}_h&=Y_h^T \, (D^TX \, DX )\, Y_h. \end{aligned}$$

A straightforward computation yields

3.17 $$\begin{aligned} \begin{aligned} D X&= Q+ {y_2 \over R} \, M, \quad {\text{ where }} \quad \\ Q&= \left[ \begin{matrix} 1& 0& 0\\ 0&  \cos ({y_3 \over R}) &  - \sin ({y_3 \over R}) \\ 0&  \sin ({y_3 \over R}) &  \cos ({y_3 \over R}) \end{matrix} \right] , \quad M= \left[ \begin{matrix} 0& 0& 0\\ 0&  0 &  - \sin ({y_3 \over R}) \\ 0&  0 &  \cos ({y_3 \over R}) \end{matrix} \right] \end{aligned} \end{aligned}$$

and

3.18 $$\begin{aligned} DX^T \, DX = I + \left( 2 {y_2 \over R} + {y_2^2 \over R^2} \right) \, C, \quad C= \left[ \begin{matrix} 0& 0& 0\\ 0&  0 &  0 \\ 0&  0 &  1 \end{matrix} \right] . \end{aligned}$$

We shall do all computations in the first period, where ${y_3 \over R}$ is of order ${1\over n}$. An interesting characteristic of the matrix *M* that we will use later is that

3.19 $$\begin{aligned} M \left[ \begin{matrix} a\\ b\\ c \end{matrix} \right] = c \left[ \begin{matrix} 0\\ - \sin ({y_3 \over R}) \\ \cos ({y_3 \over R}) \end{matrix} \right] , \quad M^T \left[ \begin{matrix} a\\ b\\ c \end{matrix} \right] = \left[ \begin{matrix} 0\\ 0 \\ - \sin ({y_3 \over R}) b +\cos ({y_3 \over R}) c \end{matrix} \right] . \end{aligned}$$

Thus,

$$\begin{aligned} {\tilde{Y}}_h^T \, {\tilde{Y}}_h&= Y_h^T \, \left( I + \left( 2 {y_2 \over R} + {y_2^2 \over R^2} \right) C \right) \, Y_h \\&= Y_h^T \, Y_h + \left( 2 {y_2 \over R} + {y_2^2 \over R^2} \right) \, Y_h^T \, C \,Y_h \end{aligned}$$

and

3.20 $$\begin{aligned} \begin{aligned} \left( {\tilde{Y}}_h^T \, {\tilde{Y}}_h\right) ^{-1}&= \, \left( I + \left( 2 {y_2 \over R} + {y_2^2 \over R^2} \right) \, g_h^{-1} \, Y_h^T \, C \, Y_h \right) ^{-1} \, g_h^{-1}, \\ \quad&{\text{ where }} \quad g_h = \, Y_h^T \, Y_h \end{aligned} \end{aligned}$$

and *C* is given by (3.18). This tells us that

$$\begin{aligned} {\tilde{A}}_h&=- \left( I + \left( 2 {y_2 \over R} + {y_2^2 \over R^2} \right) \, g_h^{-1} \, Y_h^T \, C \, Y_h \right) ^{-1} \, g_h^{-1} \, Y_h^T \, (D \, X )^T D_\omega {\tilde{\nu }}_h . \end{aligned}$$

Now we look for an expression of $D_\omega {\tilde{\nu }}_h$ in terms of $D_\omega \nu _h$. From the relation ${\tilde{Y}}_h =DX \, Y_h$, we use (3.17) and (3.19) to compute that

3.21 $$\begin{aligned} \begin{aligned} {\tilde{Y}}_{h1} \times {\tilde{Y}}_{h2}&= Q Y_{h1} \times Q Y_{h2} + {y_2 \over R} Z_h\\&\quad \quad {\text{ where }} \\ Z_h&= M \, Y_{h1} \times Q \, Y_{h2} + Q \, Y_{h1} \times M \, Y_{h2}. \end{aligned} \end{aligned}$$

and

$$\begin{aligned}&\Vert {\tilde{Y}}_{h1} \times {\tilde{Y}}_{h2} \Vert ^{-1} = \Vert Y_{h1} \times Y_{h2} \Vert ^{-1} \\&\left( 1+ 2 {y_2 \over R} { Q (Y_{h1} \times Y_{h2} ) \cdot Z_h \over \Vert Y_{h1} \times Y_{h2} \Vert ^2 } +{y_2^2 \over R^2} {\Vert Z_h \Vert ^2 \over \Vert Y_{h1} \times Y_{h2} \Vert ^2} \right) ^{-{1\over 2}}. \end{aligned}$$

Hence, we explicitly obtain that

3.22 $$\begin{aligned} {\tilde{\nu }}_h = Q \, \nu _h + \nu _h^{(1)}, \end{aligned}$$

where

$$\begin{aligned} \nu _h^{(1)}&= Q \, \nu _h \left[ \left( 1+ 2 {y_2 \over R} { Q (\nu _h ) \cdot Z_h \over \Vert Y_{h1} \times Y_{h2} \Vert } +{y_2^2 \over R^2} {\Vert Z_h \Vert ^2 \over \Vert Y_{h1} \times Y_{h2} \Vert ^2} \right) ^{-{1\over 2}} -1 \right] \\&\quad +{y_2 \over R} {Z_h \over \Vert Y_{h1} \times Y_{h2} \Vert } \left( 1+ 2 {y_2 \over R} { Q (\nu _h ) \cdot Z_h \over \Vert Y_{h1} \times Y_{h2} \Vert } +{y_2^2 \over R^2} {\Vert Z_h \Vert ^2 \over \Vert Y_{h1} \times Y_{h2} \Vert ^2} \right) ^{-{1\over 2}}. \end{aligned}$$

Later we will analyze $\nu _h^{(1)}$ in detail. For the moment we observe that

$$\begin{aligned} D_\omega {\tilde{\nu }}_h&= Q D_\omega \nu _h + ( D_\omega Q ) \nu _h + D_\omega \nu _h^{(1)}. \end{aligned}$$

Here $(D_\omega Q) \nu _h$ is the $3\times 2$ matrix whose columns are $(D_{\omega _i} Q) \nu$, $i=1,2$. Since $Q^T Q=I$, we obtain

3.23 $$\begin{aligned} \begin{aligned} (DX)^T \, D_\omega {\tilde{\nu }}_h&= (Q^T +{y_2 \over R} M^T ) \, \left( Q D_\omega \nu _h + ( D_\omega Q ) \nu _h + D_\omega \nu _h^{(1)} \right) \\&= D_\omega \nu _h +V_h \\&\quad \quad {\text{ where }}\\ V_h&= Q^T D_\omega \nu _h^{(1)} + Q^T \, (D_\omega Q) \nu _h + {y_2 \over R} M^T D_\omega {\tilde{\nu }}_h. \end{aligned} \end{aligned}$$

We insert the computations (3.20), (3.23) in the definition of ${\tilde{A}}_h$, see (3.16), and we get

3.24 $$\begin{aligned} {\tilde{A}}_h = - \, g_h^{-1} \, Y_h^T \, D_\omega \nu _h + A_{n,h}^{(1)}, \end{aligned}$$

where

$$\begin{aligned} A_{n,h}^{(1)} =&- g_h^{-1} \, Y_h^T\, V_h\\&- \left[ \left( I + \left( 2 {y_2 \over R} + {y_2^2 \over R^2} \right) \, g_h^{-1} \, Y_h^T \, C \, Y_h \right) ^{-1} - I \right] \, g_h^{-1} \, Y_h^T \, (DX)^T \, D_\omega {\tilde{\nu }}_h , \end{aligned}$$

with $V_h$ given in (3.23).

Since $H_{\Sigma _h^n} = - \, {\textrm{trace}} (A_h)$, with $A_h= - \, g_h^{-1} \, Y_h^T \, D_\omega \nu _h$, we get that

3.25 $$\begin{aligned} {\tilde{H}}_{{\tilde{\Sigma }}^n_h } (y) = H_{ \Sigma ^n_h } (y) + H^{(1)}_{n,h} (y), \quad {\text{ where }} \quad H^{(1)}_{n,h} = - \, {\textrm{trace}} \, A_{n,h}^{(1)}. \end{aligned}$$

The rest of the proof of Lemma 3.1 is devoted to analyzing $$H^{(1)}_{n,h} $$.

Let $A_n^{(1)}$ be the matrix $A_{n,h}^{(1)}$ in (3.24) taking $h=0$. We claim that

3.26 $$\begin{aligned} \begin{aligned} {\textrm{trace}} \, A_n^{(1)}&= - {\sin \theta \over R} \Biggl ( { (2-(f')^2 ) \, f \, f'' \over (1+ (f')^2 )^{5\over 2}} +{1 + 3 (f')^2 \over (1+ (f')^2 )^{3\over 2}} \Biggl )+ {a(y)\over n^2} \end{aligned} \end{aligned}$$

where *a*(*y*) is a smooth function of *y*, uniformly bounded with its derivatives, as $n \rightarrow \infty$, and it satisfies the symmetries (1.9). In addition,

3.27 $$\begin{aligned} {\textrm{trace}} \, A_{n,h}^{(1)} = {\textrm{trace}} \, A_n^{(1)} + O\left( {1\over n} \right) \, \ell (h, D_\omega h, D^2_\omega h ), \quad {\text{ as } } n \rightarrow \infty , \end{aligned}$$

where $\ell$ is a smooth function in $\omega \in \Lambda$ (see (3.1)), which is uniformly bounded, with its derivatives, as $n \rightarrow \infty$, and it satisfies the symmetries (1.9). Besides $\ell (0,0,0) = 0$, $\nabla \ell (0,0,0) \not = 0$.

The rest of this proof is devoted to establish the validity of (3.26) and (3.27).

We start with (3.26). We write that

$$\begin{aligned} A_{n}^{(1)}&= A_{n1} + A_{n2} \\ A_{n1}&=- g^{-1} \, Y^T\, V \\ A_{n2}=&- \left[ \left( I + \left( 2 {y_2 \over R} + {y_2^2 \over R^2} \right) \, g^{-1} \, Y^T \, C \, Y \right) ^{-1} - I \right] \, g^{-1} \, Y^T \, (DX)^T \, D_\omega {\tilde{\nu }} . \end{aligned}$$

To simplify notation, we write *V* for the function $V_h$ as in (3.23) for $h=0$.

We first compute the trace of $$ A_{n2}$$. Since

3.28 $$\begin{aligned} g= \left[ \begin{matrix} f^2& 0\\ 0 &  1 + (f')^2 \end{matrix} \right] , \quad g^{-1} = {1\over f^2 (1+ (f')^2) } \left[ \begin{matrix} 1 + (f')^2 & 0\\ 0 &  f^2 \end{matrix} \right] , \end{aligned}$$

we use the explicit definition of the matrix *C* (3.18) to obtain

$$\begin{aligned} g^{-1} \, Y^T \, C \, Y = {1\over 1+ (f')^2} \left[ \begin{matrix} 0 & 0\\ 0 &  1 \end{matrix} \right] . \end{aligned}$$

Hence

$$\begin{aligned} \left( I + \left( 2 {y_2 \over R} + {y_2^2 \over R^2} \right) \, g^{-1} \, Y^T \, C \, Y \right) ^{-1}&= \left[ \begin{matrix} 1 & 0\\ 0 &  \left[ 1+ \left( 2 {y_2 \over R} + {y_2^2 \over R^2} \right) {1\over 1+ (f')^2} \right] ^{-1} \end{matrix} \right] , \end{aligned}$$

and

$$\begin{aligned} \left( I + \left( 2 {y_2 \over R} + {y_2^2 \over R^2} \right) \, g^{-1} \, Y^T \, C \, Y \right) ^{-1}&-I = \left[ \begin{matrix} 0 & 0\\ 0 &  \alpha (y) \end{matrix} \right] \\ \alpha (y)&= -{ \left( 2 {y_2 \over R} + {y_2^2 \over R^2} \right) {1\over 1+ (f')^2} \over 1+ \left( 2 {y_2 \over R} + {y_2^2 \over R^2} \right) {1\over 1+ (f')^2} }. \end{aligned}$$

Observe now

$$\begin{aligned} {\textrm{trace}} (A_{n2} )&=- {\textrm{trace}} \left( \left[ \begin{matrix} 0 & 0\\ 0 &  \alpha (y) \end{matrix} \right] \, g^{-1} \, Y^T \, (DX)^T \, D_\omega {\tilde{\nu }} \right) . \end{aligned}$$

Letting

$$\begin{aligned} g^{-1} \, Y^T \, (DX)^T \, D_\omega {\tilde{\nu }} = \left[ \begin{matrix} a & b\\ c &  d \end{matrix} \right] , \end{aligned}$$

we get that

$$\begin{aligned} {\textrm{trace}} (A_{n2} )&=- \alpha (y) \, d. \end{aligned}$$

Thus we proceed with the computation of the element (2, 2) of the matrix $g^{-1} \, Y^T \, (DX)^T \, D_\omega {\tilde{\nu }}.$ Using again (3.28) we obtain that

$$\begin{aligned} d= { Y_2 \cdot \left( (DX)^T D_{y_3} {\tilde{\nu }} \right) \over 1+ (f')^2}, \end{aligned}$$

where $Y_2$ is given by (3.2). A direct computation gives

3.29 $$\begin{aligned} \begin{aligned} D_1 \nu&= {1\over \sqrt{1+ (f')^2} } \left[ \begin{matrix} -\sin \theta \\ \cos \theta \\ 0 \end{matrix}\right] , \quad \\ D_2 \nu&= \left( {1\over \sqrt{1+ (f')^2} }\right) ' \left[ \begin{matrix} \cos \theta \\ \sin \theta \\ -f' \end{matrix}\right] - {f'' \over \sqrt{1+ (f')^2}} \left[ \begin{matrix} 0 \\ 0 \\ 1\end{matrix}\right] \end{aligned} \end{aligned}$$

as consequence of (3.3), and

$$\begin{aligned} Y_2 \cdot \left( (DX)^T D_{y_3} \nu \right)&= -{f'' \over \sqrt{1+ (f')^2}} \\&\quad + \left( \cos {y_3 \over R} -1 \right) \left( {f' \over \left( \sqrt{1+ (f')^2}\right) '} \sin ^2 \theta \right. \\  &\quad \left. - \left( {f' \over \sqrt{1+ (f')^2}}\right) ' \left( 1+ {y_2 \over R}\right) \right) \\&\quad - \sin {y_3 \over R} \, \sin \theta \, \left( f' \left( {f' \over \sqrt{1+ (f')^2}}\right) ' \right. \\  &\quad \left. + {1 \over \left( \sqrt{1+ (f')^2}\right) '} \left( 1+ {y_2 \over R}\right) \right) . \end{aligned}$$

Combining these computations with the previous ones we conclude that

3.30 $$\begin{aligned} {\textrm{trace}} (A_{n2} )= - 2 {y_2 \over R} \, {f'' \over (1+ (f')^2 )^{5\over 2}} + {a_2(y)\over n^2} \quad {\text{ as } } n \rightarrow \infty , \end{aligned}$$

where $a_2$ is a smooth function, uniformly bounded as $n \rightarrow \infty$ which satisfies the symmetry assumptions (1.9).

We now treat the trace of $A_{n1}$. From (3.28) we observe that

$$\begin{aligned} {\textrm{trace}} \left( g^{-1} \left[ \begin{matrix} a & b\\ c &  d \end{matrix} \right] \right) = {1\over f^2 (1+ (f')^2) } \left( (1+ (f')^2) \, a + f^2 \, d \right) . \end{aligned}$$

This suggests that

3.31 $$\begin{aligned} \begin{aligned} {\textrm{trace}} \, A_{n1}&= -{1\over f^2 (1+ (f')^2) } \left( (1+ (f')^2) \, a + f^2 \, d \right) \\ a&= Y_1 \cdot V_1, \quad d= Y_2 \cdot V_2 \end{aligned} \end{aligned}$$

where $V_1$ and $V_2$ are the first and second columns of the $3\times 2$ matrix

$$\begin{aligned} V= Q^T D_\omega \nu ^{(1)} + Q^T \, (D_\omega Q) \nu + {y_2 \over R} M^T ( D_\omega \nu + ( D_\omega Q ) \nu + D_\omega \nu ^{(1)}). \end{aligned}$$

In order to find $V_1$ and $V_2$, we observe that

$$\begin{aligned} {y_2 \over R} M^T ( ( D_\omega Q ) \nu + D_\omega \nu ^{(1)}) = O\left( {1\over n^2}\right) \quad {\text{ as } } n \rightarrow \infty . \end{aligned}$$

We thus turn to analyze $$Q^T D_\omega \nu ^{(1)} + {y_2 \over R} M^T D_\omega \nu + Q^T \, (D_\omega Q) \nu $$. Calling $$V_j^\#$$, $$V_j^*$$ and $$V_j^{**}$$ the columns respectively of $$Q^T D_\omega \nu ^{(1)}$$, $${y_2 \over R} M^T D_\omega \nu $$ and $$ Q^T \, (D_\omega Q) \nu _h$$, we have that

$$\begin{aligned} V_j = V_j^\# + V_j^* +V_j^{**}+O\left( {1\over n^2}\right) \quad j=1,2, \quad {\text{ as } } n \rightarrow \infty . \end{aligned}$$

We start from ${y_2 \over R} M^T D_\omega \nu$. From (3.29) we get the columns $V_j^*$ of ${y_2 \over R} M^T D_\omega \nu$ are

3.32 $$\begin{aligned} V_1^* = O\left( {1\over n^2}\right) , \quad V_2^* = - {y_2 \over R} \left( {f' \over \sqrt{1+ (f')^2}} \right) ' \, \left[ \begin{matrix} 0 \\ 0 \\ 1\end{matrix}\right] + O\left( {1\over n^2}\right) \quad {\text{ as } } n \rightarrow \infty .\nonumber \\ \end{aligned}$$

Next we consider $Q^T \, (D_\omega Q) \nu$. From (3.17) we easily check that

3.33 $$\begin{aligned} V_1^{**} = O\left( {1\over n^2}\right) , \quad V_2^{**} = {1\over R} {1\over \sqrt{1+ (f')^2}} \, \left[ \begin{matrix} 0 \\ f' \\ \sin \theta \end{matrix}\right] + O\left( {1\over n^2}\right) \quad {\text{ as } } n \rightarrow \infty .\nonumber \\ \end{aligned}$$

We turn now to $Q^T D_\omega \nu ^{(1)}$ and its columns $V_j^\#$. From (3.22) we get that

$$\begin{aligned} \nu ^{(1)} = {y_2 \over R} {1\over \Vert Y_1 \times Y_2 \Vert } \left[ Z - (\nu \cdot Z) \nu \right] + O\left( {1\over n^2}\right) \quad {\text{ as } } n \rightarrow \infty , \end{aligned}$$

where *Z* is the vector field $Z_h$ introduced in (3.21) taking $h=0$. Using (3.19) and (3.2), a close inspection to *Z* gives

$$\begin{aligned} Z= Y_1 \times \left[ \begin{matrix} 0 \\ 0 \\ 1 \end{matrix}\right] + O({1\over n}) = f \left[ \begin{matrix} \cos \theta \\ \sin \theta \\ 0 \end{matrix}\right] + O({1\over n}), \quad {\text{ as } } n \rightarrow \infty . \end{aligned}$$

Since $\Vert Y_1 \times Y_2 \Vert = f \sqrt{1+ (f')^2}$, $y_2 = f \sin \theta$ and $\nu \cdot Z = {f \over \sqrt{1+ (f')^"}} + O({1\over n})$, we get

$$\begin{aligned} \nu ^{(1)}&= { \sin \theta \over R} v + O\left( {1\over n^2}\right) , \quad v= {f \over \sqrt{1+ (f')^2} }\left( \left[ \begin{matrix} \cos \theta \\ \sin \theta \\ 0 \end{matrix}\right] -{\nu \over \sqrt{1+ (f')^2} } \right) . \end{aligned}$$

We get that the columns of $Q^T D_\omega \nu ^{(1)}$ are

3.34 $$\begin{aligned} \begin{aligned} V_1^\#:= Q^T \, D_1 \nu ^{(1)}&= { \cos \theta \over R} v + {\sin \theta \over R} D_1 v + O\left( {1\over n^2}\right) \\ V_2^\#:= Q^T \, D_2 \nu ^{(1)}&= {\sin \theta \over R} D_2 v + O\left( {1\over n^2}\right) \end{aligned} \end{aligned}$$

as $n \rightarrow \infty$. We recall that $D_1$ stands for $D_\theta$, and $D_2$ for $D_{x_3} = \, '$. From (3.32)–(3.33)–(3.34) and using the fact that $Y_1 \cdot v=0$ we obtain that

$$\begin{aligned} Y_1 \cdot V_1&= {\sin \theta \over R} \, Y_1 \cdot D_1 v + O\left( {1\over n^2}\right) \\ Y_2 \cdot V_2&= - {y_2 \over R} \left( {f' \over \sqrt{1+ (f')^2}} \right) ' + {\sin \theta \over R} \, \sqrt{1+ (f')^2} \\&\quad + {\sin \theta \over R} Y_2 \cdot D_2 v + O\left( {1\over n^2}\right) \end{aligned}$$

as $n \rightarrow \infty$. It is straightforward to check that

$$\begin{aligned} D_1 v&= {f \, (f')^2 \over (1+ (f')^2 )^{3\over 2} } \left[ \begin{matrix} - \sin \theta \\ \cos \theta \\ 0 \end{matrix}\right] \\ D_2 v&= \left( {f \over \sqrt{1+ (f')^2} } \right) ' \left[ \begin{matrix} \cos \theta \\ \sin \theta \\ 0 \end{matrix}\right] - \left( {f \over 1+ (f')^2} \right) ' \nu - {f \over 1+ (f')^2} D_2 \nu . \end{aligned}$$

With these computations at hand and using that $y_2 = f \, \sin \theta$, we conclude that

$$\begin{aligned} Y_1 \cdot V_1&= {\sin \theta \over R} \,{ f^2 \, (f')^2 \over (1+ (f')^2 )^{3\over 2} } \, + O\left( {1\over n^2}\right) \\ Y_2 \cdot V_2&= - {y_2 \over R} \left( {f' \over \sqrt{1+ (f')^2}} \right) ' + {\sin \theta \over R} \, \sqrt{1+ (f')^2} \\&\quad + {\sin \theta \over R} \, f' \, \left( {f \over \sqrt{1+ (f')^2} } \right) ' + {\sin \theta \over R} {f \, f'' \over (1+ (f')^2 )^{3\over 2}} + O\left( {1\over n^2}\right) \\&= {\sin \theta \over R} \Biggl [ - { (f')^2 \, f \, f'' \over (1+ (f')^2 )^{3\over 2}} +{1 + 2 (f')^2 \over (1+ (f')^2 )^{1\over 2}} \Biggl ] + O\left( {1\over n^2}\right) \end{aligned}$$

as $n \rightarrow \infty$. From (3.31) we conclude that

3.35 $$\begin{aligned} {\textrm{trace}} \, A_{n1} = -{\sin \theta \over R} \, \Biggl ( -{ (f')^2 \, f \, f'' \over (1+ (f')^2 )^{5\over 2}} +{1 + 3 (f')^2 \over (1+ (f')^2 )^{3\over 2}} \Biggl ) + {a_1 (y)\over n^2} \end{aligned}$$

where $a_1(y)$ is a smooth function of *y*, uniformly bounded with its derivatives, as $n \rightarrow \infty$. Combining this result with (3.30) we get that

$$\begin{aligned} {\textrm{trace}} \, A_n^{(1)}&= - {\sin \theta \over R} \Biggl ( { (2-(f')^2 ) \, f \, f'' \over (1+ (f')^2 )^{5\over 2}} +{1 + 3 (f')^2 \over (1+ (f')^2 )^{3\over 2}} \Biggl )+ {a (y)\over n^2}, \end{aligned}$$

where *a*(*y*) is a smooth function of *y*, uniformly bounded with its derivatives, as $n \rightarrow \infty$. To complete the proof of (3.26) we now show that the function $a_1(y)$ in (3.35) satisfies the symmetries (1.9). We see from (3.35) that this is consequence of the fact that

$$\begin{aligned} y \rightarrow {\textrm{trace}} \, A_{n1} (y) \end{aligned}$$

satisfies (1.9). Going back to formula (3.31), we shall prove that the functions in (3.31)

$$\begin{aligned} a = a(\theta , y_3), \quad d= d(\theta , y_3) \end{aligned}$$

are even in $\theta$, even in $y_3$ and periodic in $y_3$ of period *T*. The periodicity in $y_3$ follows from the fact that all functions involved in the definition of *a* and *d* are *T*-periodic in $y_3$. The remaining symmetries can be verified checking that the evenness of the components of the columns $V_1$ and $V_2$ in (3.31) have the form

$$\begin{aligned} V_1= \left[ \begin{matrix} { \text{ even } \text{ in } } y_3, { \text{ even } \text{ in } } \theta \\ { \text{ even } \text{ in } } y_3, { \text{ odd } \text{ in } } \theta \\ {\text{ any } \text{ function }} \end{matrix} \right] , \quad V_2= \left[ \begin{matrix} { \text{ odd } \text{ in } } y_3, { \text{ odd } \text{ in } } \theta \\ { \text{ odd } \text{ in } } y_3, { \text{ even } \text{ in } } \theta \\ { \text{ even } \text{ in } } y_3, { \text{ even } \text{ in } } \theta \end{matrix} \right] . \end{aligned}$$

We check this for $V_1$, which is given by

$$\begin{aligned} V_1= Q^T D_\theta \nu ^{(1)} + {y_2 \over R} M^T D_\theta {\tilde{\nu }}. \end{aligned}$$

Since the first two component of $M^T D_\theta {\tilde{\nu }}$ are zero (because of (3.19)), we just need to check $Q^T D_\theta \nu ^{(1)}$. We have

$$\begin{aligned} \nu ^{(1)} = \left[ \begin{matrix} { \text{ even } \text{ in } } y_3, { \text{ odd } \text{ in } } \theta \\ { \text{ odd } \text{ in } } y_3, { \text{ even } \text{ in } } \theta \\ { \text{ even } \text{ in } } y_3, { \text{ even } \text{ in } } \theta \end{matrix} \right] , \quad D_\theta \nu ^{(1)} = \left[ \begin{matrix} { \text{ even } \text{ in } } y_3, { \text{ even } \text{ in } } \theta \\ { \text{ odd } \text{ in } } y_3, { \text{ odd } \text{ in } } \theta \\ { \text{ even } \text{ in } } y_3, { \text{ odd } \text{ in } } \theta \end{matrix} \right] \end{aligned}$$

and

$$\begin{aligned} Q^T D_\theta \nu ^{(1)} = \left[ \begin{matrix} { \text{ even } \text{ in } } y_3, { \text{ even } \text{ in } } \theta \\ { \text{ even } \text{ in } } y_3, { \text{ odd } \text{ in } } \theta \\ { \text{ odd } \text{ in } } y_3, { \text{ odd } \text{ in } } \theta \end{matrix} \right] , \end{aligned}$$

which is what expected.

For $V_2$, we have

$$\begin{aligned} V_2 = Q^T (D_{y_3} Q) [\nu ] +Q^T D_{y_3} \nu ^{(1)} + {y_2 \over R} M^T D_{y_3} {\tilde{\nu }}. \end{aligned}$$

The desired form of $V_2$ follows from the fact that $Q\nu$, ${\tilde{\nu }}$ and $\nu ^{(1)}$ have the same structure

$$\begin{aligned} \left[ \begin{matrix} { \text{ even } \text{ in } } y_3, { \text{ odd } \text{ in } } \theta \\ { \text{ odd } \text{ in } } y_3, { \text{ even } \text{ in } } \theta \\ { \text{ even } \text{ in } } y_3, { \text{ even } \text{ in } } \theta \end{matrix} \right] . \end{aligned}$$

This completes the proof of (3.26).

We now turn to (3.27). We observe that

$$\begin{aligned} A_{n,h}^{(1)} =&A_n^{(1)} - B_1 - B_2 \\ B_1&= g_h^{-1} \, Y_h^T\, V_h - g^{-1} \, Y^T\, V \\ B_2&= \left[ \left( I + \left( 2 {y_2 \over R} + {y_2^2 \over R^2} \right) \, g_h^{-1} \, Y_h^T \, C \, Y_h \right) ^{-1} - I \right] \, g_h^{-1} \, Y_h^T \, (DX)^T \, D_\omega \nu _h \\&\quad - \left[ \left( I + \left( 2 {y_2 \over R} + {y_2^2 \over R^2} \right) \, g^{-1} \, Y^T \, C \, Y \right) ^{-1} - I \right] \, g^{-1} \, Y^T \, (DX)^T \, D_\omega \nu . \end{aligned}$$

We further split

$$\begin{aligned} B_1&= g_h^{-1} \, Y_h^T\, (V_h - V) \, + \, g_h^{-1} \, (Y_h^T - Y^T) \, V \, + \, (g_h^{-1} - g^{-1} ) \, Y^T\, V \end{aligned}$$

and

$$\begin{aligned} B_2&= \left[ \left( I + \left( 2 {y_2 \over R} + {y_2^2 \over R^2} \right) \, g_h^{-1} \, Y_h^T \, C \, Y_h \right) ^{-1} - I \right] \\&\quad \times \, \left( g_h^{-1} \, Y_h^T \, (DX)^T \, D_\omega \nu _h - g^{-1} \, Y^T \, (DX)^T \, D_\omega \nu \right) \\&\quad + \left[ \left( I + \left( 2 {y_2 \over R} + {y_2^2 \over R^2} \right) \, g_h^{-1} \, Y_h^T \, C \, Y_h \right) ^{-1}\right. \\  &\quad \left. - \left( I + \left( 2 {y_2 \over R} + {y_2^2 \over R^2} \right) \, g^{-1} \, Y^T \, C \, Y \right) ^{-1} \right] \\&\quad \times \, g^{-1} \, Y^T \, (DX)^T \, D_\omega \nu . \end{aligned}$$

We claim that

3.36 $$\begin{aligned} \begin{aligned} {\textrm{trace}} (B_1 + B_2)&= {1\over n} \, \ell (h, D_\omega h, D_\omega ^2 h), \quad {\text{ as }} \quad n \rightarrow \infty \end{aligned} \end{aligned}$$

where $\ell$ is a smooth function in $\omega \in \Lambda$ (see (3.1)), which is uniformly bounded, together with its derivatives, as $n \rightarrow \infty$. In addition, $\ell (0,0,0) = 0$, $\nabla \ell (0,0,0) \not = 0$.

This fact is consequence of

$$\begin{aligned} Y_h&= Y + h D_\omega \nu + \nu ^T D_\omega h,\\ g_h&= g + h \, \left( Y^T D_\omega \nu + D_\omega \nu ^T Y \right) + Y^T \nu ^T D_\omega h + D_\omega h^T \nu Y\\&\quad + h^2 D_\omega \nu ^T D_\omega \nu + h D_\omega \nu ^T \nu ^T D_\omega h + h D_\omega h^T \nu D_\omega \nu + D_\omega h^T \nu \nu ^T D_\omega h, \\&\quad {\text{ and }} \\ \nu _h&= \nu + {\vec {\ell }}_1 (h, D_\omega h), \end{aligned}$$

where ${\vec {\ell }}_1$ is an explicit vector, which is a smooth function of $\omega$, such that ${\vec {\ell }}_1 (0, 0) =0$, $D {\vec {\ell }}_1 (0, 0) \not =0.$ Using that $2\, \pi \, R = n \, T$, from (3.22) and (3.23) we obtain

$$\begin{aligned} \nu _h^{(1)}&= {1\over n} \, \nu ^{(1)}_* + {1\over n} {\vec {\ell }}_1 (h, D_\omega h)\\ V_h&= {1\over n} \, V_* + {1\over n} {\vec {\ell }}_2 (h, D_\omega h, D_\omega ^2 h), \end{aligned}$$

with $\nu ^{(1)}_*$ and $V_*$ smooth functions of $\omega$, which are uniformly bounded (with their derivatives) as $n \rightarrow \infty$; ${\vec {\ell }}_1$ has the same properties as before, and ${\vec {\ell }}_2$ is an explicit vector, which is a smooth function of $\omega$, such that ${\vec {\ell }}_2 (0, 0,0) =0$, $D {\vec {\ell }}_2 (0, 0,0) \not =0.$

Combining these expansions with (3.27), we obtain (3.10). We conclude with the remark that $y \rightarrow \ell (h, D_\omega h , D_\omega ^2\,h) (y)$ in (3.36) satisfies (1.9), as consequence of Remark 3.1 and of (3.26)–(3.25). $\square$

### Proof of Lemma 3.2

Recall that

3.37 $$\begin{aligned} H_{\Sigma _h^n} = -\, \hbox {trace}\, (A_h), \quad {\text{ where }} \quad A_h= -g_h^{-1} Y_h^T B_h. \end{aligned}$$

We refer to (3.13) for $g_h$, to (3.11) for $Y_h$ and (3.14) for $B_h$.

Recall the parametrization of surface $\Sigma _h^n$ given by

$$\begin{aligned} y_h(w) = y(w) + h(w) \nu (w), \quad \end{aligned}$$

and

$$\begin{aligned} Y_h = D_w (y + h \nu ) = Y+ B h + \nu D_\omega h, \quad B= D_\omega \nu . \end{aligned}$$

The metric $g_h$ on $\Sigma _h^n$ is thus given by

3.38 $$\begin{aligned} \begin{aligned} g_h&= ( Y+ B h + \nu D_\omega h ) ^T (Y+ Bh + \nu D_\omega h )\\&= Y^TY + h ( B^TY + Y^TB ) + h^2 B^TB\\&\quad + Y^T \nu D_\omega h + D_\omega h^T \nu ^T Y + D_\omega h^T \nu ^T \nu D_\omega h \end{aligned}\end{aligned}$$

Recall that $\nu ^T \nu =1$. The relations $\nu ^T Y_i =0 = \nu ^T Y_j$ imply

$$\begin{aligned} \nu ^T Y = Y^T\nu =0, \end{aligned}$$

and after differentiation,

$$\begin{aligned} \nu ^T {\partial } _j Y_i + B_j^T Y_i= 0=\nu ^T{\partial } _i Y_j + B_i^T Y_j. \end{aligned}$$

Since ${\partial } _j Y_i= {\partial } _i Y_j$, we get $(B^T Y)_{ij} = B_j^T Y_i = B_i^T Y_j = (B^T Y)_{ji}.$ Thus the matrix $B^T Y$ is symmetric, and therefore,

$$\begin{aligned} B^TY = Y^TB. \end{aligned}$$

We observe that the vectors $B_i$ belong to the tangent space, which is spanned by the $Y_i's$. Hence for a column vector $\alpha _j$ we have $B_i = Y \alpha _{i}.$ Hence $\alpha _i = (Y^T Y)^{-1} Y^T B_i$. It follows that

$$\begin{aligned} B = Y (Y^T Y)^{-1} Y^T B. \end{aligned}$$

Using these relations in (3.38) we obtain

$$\begin{aligned} \begin{aligned} g_h =&Y^TY + 2h Y^TB + h^2B^TB+ D_\omega h^T D_\omega h \\ =&g(0) \left[ I + 2h (Y^TY)^{-1} Y^TB + h^2 (Y^TY)^{-1}B^TB \right] + D_\omega h^T D_\omega h. \end{aligned} \end{aligned}$$

We observe that

$$\begin{aligned} \begin{aligned} {[} (Y^TY)^{-1} Y^TB]^2 =&(Y^TY)^{-1} Y^TB (Y^TY)^{-1} Y^TB\\ =&(Y^TY)^{-1} B^TY (Y^TY)^{-1} Y^TB = (Y^TY)^{-1} B^TB, \end{aligned} \end{aligned}$$

and hence,

3.39 $$\begin{aligned} g_h = g(0) [ I- hA ]^2 \left( I + \left( g(0) [ I- hA ]^2 \right) ^{-1} D_\omega h^T D_\omega h \right) , \end{aligned}$$

where *A* is the matrix

$$\begin{aligned} A = - [\,Y^TY]^{-1} Y^T B. \end{aligned}$$

Next we compute an expansion in *h* for the normal vector to $\Sigma _h^n$,

$$\begin{aligned} \nu _h = \frac{Y_{1h} \times Y_{2h}}{|Y_{1h} \times Y_{2h}|}. \end{aligned}$$

It holds that

$$\begin{aligned} \begin{aligned} Y_{h1} \times Y_{h2} =&(Y_1 + hB_1 + \nu D_1 h) \times (Y_2 + hB_2 + \nu D_2 h ) \\ =&Y_1 \times Y_2+ v (h) + \ q (h, D_\omega h ), \end{aligned} \end{aligned}$$

where $B_i$, $i=1,2$, are the columns of *B*, $D_i$ stands for $D_{\omega _i}$, $i=1,2$,

$$\begin{aligned} \begin{aligned} v (h) =&D_2 h\, ( Y_1\times \nu ) + D_1 h \, (\nu \times Y_2) + h\, [ D_2h\, (B_1\times \nu ) + D_1h (\nu \times B_2) ] \\ =&h\, (B_1 \times Y_2 + Y_1\times B_2 ) + D_2h \, (Y_1\times \nu ) + D_1 h \, (\nu \times Y_2) \end{aligned} \end{aligned}$$

and $q (h, D_\omega h)$ a smooth function, with $q(0,0)= Dq (0,0)=0$ and uniformly bounded as $n \rightarrow \infty$. Then

$$\begin{aligned} \begin{aligned} \nu _h =&\nu - \nu \frac{\nu \cdot v (h) }{ |Y_1\times Y_2 | } + \frac{ v (h) }{ |Y_1\times Y_2 | } + {\hat{\nu }}_h\\ =&\nu + \frac{1}{ |Y_1\times Y_2 | } Y (Y^TY)^{-1} Y^T v (h) + {\hat{\nu }}_h.\end{aligned} \end{aligned}$$

Hence we have

3.40 $$\begin{aligned} \begin{aligned} B_h = D_\omega \nu _h =&D_\omega \nu + D_\omega \left( \frac{1}{ |Y_1\times Y_2 | } Y \, (Y^TY)^{-1} Y^T v (h) \right) + D_\omega {\hat{\nu }}_h\\ =&B + D_\omega \left( \frac{1}{ |Y_1\times Y_2 |} ( Y g^{-1}Y^T v (h) )\right) + q(h, D_\omega h, D_\omega ^2 h) \end{aligned} \end{aligned}$$

where $q (h, D_\omega h, D_\omega ^2 h )$ a smooth function, with $q(0,0, 0)= Dq (0,0,0)=0$ and uniformly bounded as $n \rightarrow \infty$. Inserting (3.39)–(3.40) into (3.37), we get

$$\begin{aligned} A_h&= A - 2hAg^{-1}Y^TB - g^{-1} B^TB h \\&\quad - g^{-1} Y^T D \left( \frac{1}{ |Y_1\times Y_2 |} Y(Y^TY)^{-1} Y^T \, v (h) \right) + q (h, D_\omega h, D_\omega ^2 h) \end{aligned}$$

for a quadratic term *q* with the same properties as the one in (3.40). Observe now that

$$\begin{aligned} -A^2 = A g^{-1}Y^TB, \quad g^{-1}B^T B = A^2, \end{aligned}$$

hence

3.41 $$\begin{aligned} \begin{aligned} A_h&= A + hA^2 - g^{-1} Y^T D \left( \frac{1}{ |Y_1\times Y_2 |} Y(Y^TY)^{-1} Y^T v (h) \right) \\&\quad + q(h, D_\omega h, D_\omega ^2 h). \end{aligned} \end{aligned}$$

On the other hand, we use the vector identity

$$\begin{aligned} a\cdot (b\times c) = \det [ a\, b\, c] \end{aligned}$$

to get

$$\begin{aligned} \begin{aligned} \nu \times Y_2 \cdot Y_1 =&Y_1 \times \nu \cdot Y_2 = -\sqrt{\det g } \\ \nu \times Y_2 \cdot Y_2 =&\nu \times Y_1 \cdot Y_1 = 0,\\ (B_1\times Y_2 + Y_1 \times B_2) \cdot Y_1 =&(B_1\times Y_2 + Y_1 \times B_2)\cdot Y_2 = 0, \end{aligned} \end{aligned}$$

and we get the simple relation

$$\begin{aligned} Y^T v (h) = - \sqrt{\det g }D^T h \end{aligned}$$

which simplifies formula (3.41) to

$$\begin{aligned} \begin{aligned} A_h =&A + hA^2 + g^{-1} Y^T D_\omega ( Y g^{-1} D_\omega ^T h) + q(h, D_\omega h, D_\omega ^2 h). \end{aligned} \end{aligned}$$

From here we obtain

3.42 $$\begin{aligned} \begin{aligned} {\textrm{trace}} \, A_h&= {\textrm{trace}} \, A + h |A|^2 + {\textrm{trace}} \, \left( g^{-1} Y^T D( Y g^{-1} D^T h) \right) \\&\quad + q(h, D_\omega h, D_\omega ^2 h ). \end{aligned} \end{aligned}$$

The function *q* is a smooth function of *y*, uniformly bounded with its derivatives as $n \rightarrow \infty$. It depends of *h*, $D_\omega h$ and $D_\omega ^2 h$ with

$$\begin{aligned} q(0,0,0)=0, \quad D q (0,0,0) = 0, \quad D^2 q(0,0,0) \not = 0. \end{aligned}$$

We now focus on the term $g^{-1} Y^T D_\omega ( Y g^{-1} D_\omega ^T h)$. We drop $\omega$ from the notation $D_\omega$. We write

$$\begin{aligned} g^{-1} Y^T D( Y g^{-1} D^T h)&=g^{-1} \left[ D_1 D^T h \, | \, D_2 D^T h \right] \\&\quad + \left[ D_1 g^{-1} \ |\ D_2 g^{-1} \right] D^Th \\&\quad + \left[ g^{-1} Y^T D_1Y g^{-1}D^Th \ |\ g^{-1} Y^T D_2Y g^{-1} D^Th\right] \end{aligned}$$

A direct computation gives

$$\begin{aligned}&{\textrm{trace }} \left( g^{-1} \left[ D_1 D^T h \, | \, D_2 D^T h \right] \right) \\&\quad = {\textrm{trace }} \left( \left[ \begin{matrix} g^{11} &  g^{12} \\ g^{12} &  g^{22} \end{matrix}\right] \left[ \begin{matrix} D_{11} h &  D_{12} h \\ D_{12} h &  D_{22} h \end{matrix}\right] \right) \\&\quad = g^{ij} D_{ij} h,\\&{\textrm{trace }} \left( \left[ D_1 g^{-1} D^Th \ |\ D_2 g^{-1} D^Th \right] \right) \\&\quad = {\textrm{trace }} \left( \left[ \begin{matrix} D_1 g^{11} &  D_1 g^{12} \\ D_1 g^{12} &  D_1 g^{22} \end{matrix}\right] \left[ \begin{matrix} D_{1} h \\ D_2 h \end{matrix} \right] \, | \, \left[ \begin{matrix} D_2g^{11} &  D_2 g^{12} \\ D_2 g^{12} &  D_2 g^{22} \end{matrix}\right] \left[ \begin{matrix} D_{1} h \\ D_2 h \end{matrix} \right] \right) \\&\quad = D_i g^{ij} D_j h \end{aligned}$$

and

3.43 $$\begin{aligned} \begin{aligned}&{\textrm{trace }}\, [g^{-1} Y^T D( Y g^{-1} D^T h) ] \\&\quad = {\textrm{trace }}\, \left[ g^{-1} Y^T D_1Y g^{-1}D^Th \ |\ g^{-1} Y^T D_2Y g^{-1} D^Th\right] \\&\quad = [g^{-1} Y^T D_1Y g^{-1}D^Th]_1 + [g^{-1} Y^T D_2Y g^{-1} D^Th]_2\\&\quad = [ (g^{-1}Y^TD_1Y)_{11} + (g^{-1}Y^TD_2Y)_{21} ] \, [ g^{11} D_1 h + g^{12} D_2 h ]\\&\qquad + [ (g^{-1}Y^TD_1Y)_{12} + (g^{-1}Y^TD_2Y)_{22} ]\, [ g^{21} D_1 h + g^{22} D_2 h ]. \end{aligned} \end{aligned}$$

Since

$$\begin{aligned} D_1Y_2 =&D_2Y_1,\quad D_1Y_1\cdot Y_2 = D_1 g_{12} - \frac{1}{2} D_2 g_{11}, \quad \\&D_2Y_2\cdot Y_1 = D_2 g_{12} - \frac{1}{2} D_1 g_{22}, \end{aligned}$$

we obtain

$$\begin{aligned} Y^T D_1Y= &   \left[ \begin{array}{cc} \frac{1}{2} D_1 g_{11} &  \frac{1}{2} D_2 g_{11} \\ D_1 g_{12} - \frac{1}{2} D_2 g_{11} &  \frac{1}{2} D_1 g_{22} \end{array} \right] \\ Y^T D_2Y= &   \left[ \begin{array}{cc} \frac{1}{2} D_2 g_{11} &  D_2 g_{12} - \frac{1}{2} D_1 g_{22} \\ \frac{1}{2} D_1 g_{22} &  \frac{1}{2} D_2 g_{22} \end{array} \right] . \end{aligned}$$

We have

$$\begin{aligned} (g^{-1}Y^TD_1Y)_{11} =&\frac{1}{2\det g} [ g_{22} D_1 g_{11} + g_{12} D_2 g_{11} - 2 g_{12} D_1 g_{12} ] \\ (g^{-1}Y^TD_1Y)_{21} =&\frac{1}{2\det g} [ g_{11} D_1 g_{22} - g_{12} D_2 g_{11} ]\\ (g^{-1} Y^T D_1Y)_{12} =&\frac{1}{2\det g} [g_{22}D_2 g_{11} - g_{12} D_1 g_{22} ]\\ (g^{-1} Y^T D_2Y)_{22} =&\frac{1}{2\det g} [g_{11}D_2 g_{22} + g_{12} D_1 g_{22} - 2 g_{12} D_2 g_{12} ]. \end{aligned}$$

From the above formulas we get

$$\begin{aligned}&{\textrm{trace }}\, [g^{-1} Y^T D( Y g^{-1} D^T h) ] \\&\quad = \frac{1}{2\det g} \left[ g_{22} D_1 g_{11} + g_{11} D_1 g_{22} - 2 g_{12} D_1 g_{12} \right] \left[ g^{11} D_1 h + g^{12} D_2 h \right] \\&\quad \quad + \frac{1}{2\det g} \left[ g_{22} D_2 g_{11} + g_{11} D_2 g_{22} - 2 g_{12} D_2 g_{12} \right] \, \left[ g^{21} D_1 h + g^{22} D_2 h \right] \\&\quad = \frac{1}{2} \frac{D_1 \det g}{\det g} \left[ g^{11} D_1 h + g^{12} D_2 h \right] \ + \ \frac{1}{2} \frac{D_2 \det g }{\det g} \, \, \left[ g^{21} D_1 h + g^{22} D_2 h \right] . \end{aligned}$$

Using these expressions in formula (3.43), we obtain from (3.41) and (3.42),

$$\begin{aligned} H_{\Sigma _h^n } = H_{\Sigma ^n } - J_\Sigma [h] + q(h, D_\omega h, D_\omega ^2 h), \end{aligned}$$

where

$$\begin{aligned} J_{\Sigma ^n} [h] = \frac{1}{2 \sqrt{\det g}} {\partial } _j ( g^{ij} \sqrt{\det g} {\partial } _j h) + |A|^2 h, \end{aligned}$$

and $q(h, D_\omega h, D_\omega ^2 h)$ has the same properties as in (3.42). We conclude with the remark that $y \rightarrow q (h, D_\omega h , D_\omega ^2\,h) (y)$ in satisfies (1.9), as consequence of Remark 3.1, the explicit expression of the Jacobi operator *J* and the fact that $H_{\Sigma ^n} (y) =2.$ This completes the proof of Lemma 3.2. $\square$

## Expansion of the Interaction Term

Let us consider a small smooth function *h*(*y*) defined on $\Sigma$ and the *normal graph* $\Sigma ^n_h$ defined as in (1.7). We also assume that the perturbation $h: \Sigma \rightarrow {\mathbb {R}}$ satisfies the symmetries (1.9).

For a point ${\tilde{y}}_h \in {\tilde{\Sigma }}^n_h = X (\Sigma ^n_h)$ we want to compute the interaction term

$$\begin{aligned} \int _{{\tilde{\Omega }}^n_h} {d {\tilde{x}} \over |{\tilde{y}}_h - {\tilde{x}}| }. \end{aligned}$$

For $y \in \Sigma ^n$, we write

4.1 $$\begin{aligned} {\tilde{N}}_{\Sigma ^n_h} (y ) = \int _{{\tilde{\Omega }}^n_h}\dfrac{d \tilde{x}}{|X(y_h) -{\tilde{x}}|},\quad {\tilde{y}}_h = X (y_h) \in {\tilde{\Sigma }}^n_h, \quad y_h \in \Sigma ^n_h, \end{aligned}$$

where

$$\begin{aligned} y_h = y + h (y) \nu (y) \end{aligned}$$

and $\nu (y)$ is the unit normal vector at $y\in \Sigma ^n$ as in (3.3).

Thanks to symmetries, it is enough to know ${\tilde{N}}_{\Sigma ^n_h} (y )$ at points *y* in $\Sigma _0$, the first piece of the truncated Delaunay $\Sigma ^n$ as defined in (1.5), that is,

$$\begin{aligned} \Sigma _0= \{\, y \in \Sigma \mid -{T \over 2} \le y_3 < {T\over 2} \}. \end{aligned}$$

Indeed, by construction any point ${\tilde{y}}_h \in {\tilde{\Sigma }}^n_h$ is the rotation of a multiple of ${2\pi \over n}$ of a point ${\tilde{y}}_{0h} = X(y_{0h})$, with $y_{0\,h} = y_0 + h(y_0) \nu (y_0)$, $y_0 \in \Sigma _0$, see (1.14). Besides, the domain of integration ${\tilde{\Omega }}^n_h$ in (1.13) is invariant under rotations. Thus in this section we will take points of the form

4.2 $$\begin{aligned} \begin{aligned} {\tilde{y}}_h&= X(y_h), \quad y_h = y + h(y) \nu (y), \quad y = \left( {\bar{y}}, y_{3} \right) \in \Sigma _0, \quad {\bar{y}} = (y_{1}, y_{2}), \quad \\ {\bar{y}}&= f(y_{3} ) \left( \cos \phi , \sin \phi \right) = f(y_{3}) \, e^{i \phi } \,, \quad {\text{ for } \text{ some }} \quad \phi \in [0,2\pi ]\\&\quad -{T\over 2} \le y_{3} \le {T\over 2}. \end{aligned} \end{aligned}$$

Our main result is the following:

### Proposition 4.1

Let $\alpha \in (0,1)$ and $h: \Sigma ^n \rightarrow {\mathbb {R}}$ be a small $C^{2, \alpha }$ function satisfying (1.9). Let $y \in \Sigma _0$ as in (4.2). Then

4.3 $$\begin{aligned} \begin{aligned} {\tilde{N}}_{ \Sigma ^n_h} (y)&={2|\Omega _0| \over T} \,\log n \, + F_0^n (y_{3} ) - y_{2} \, {2\pi |\Omega _0 | \over T^2} \, {\log n \over n} \, \\&\quad + \frac{1}{n} F_1^n (y)+ \, \left( {2 \over T} \, \log n \, + b(y) \, \right) \, \int _{\Sigma _0 } h \\&\quad +\, \ell _1 [h] (y) + \, {1\over n} \ell _2 [h] (y) \end{aligned} \end{aligned}$$

where $|\Omega _0|$ is the volume of the region enclosed by $\Sigma _0$ and *T* is the period of the Delaunay surface $\Sigma$. The functions $F_0^n$, $F_1^n$ and *b* are smooth functions of their variables, which are uniformly bounded, together with their derivatives, as $n \rightarrow \infty$. The function $F_0^n (y_3)$ is even and periodic of period *T*, the functions $F_1^n (y)$ and *b* satisfy the symmetries (1.9). Moreover, $\ell _1$, $\ell _2$ are smooth functions, uniformly bounded, together with their derivatives, as $n \rightarrow \infty$, and $\ell _i (0) = 0$, $\nabla _h \ell _i (0) \not = 0$, $i=1,2$. They both satisfy the symmetries (1.9). Besides, if *h* is also even with respect to $y_2$ then $\ell _1[h]$ is even in $y_2$.

The proof of this Proposition follows from two results. First we assume that $h=0$ and for a point ${\tilde{y}} \in {\tilde{\Sigma }}^n$ we compute

$$\begin{aligned} \int _{{\tilde{\Omega }}^n} {d {\tilde{x}} \over |{\tilde{y}} - {\tilde{x}}| }. \end{aligned}$$

We write

4.4 $$\begin{aligned} N_{\Sigma ^n} (y) = \int _{{\tilde{\Omega }}^n}\dfrac{d {\tilde{x}}}{|X(y) -{\tilde{x}}|},\quad {\tilde{y}} = X (y) \in {\tilde{\Sigma }}^n, \quad y \in \Sigma ^n. \end{aligned}$$

### Lemma 4.1

Let $y \in \Sigma _0$ as in (4.2). Then

4.5 $$\begin{aligned} \begin{aligned} N_{ \Sigma ^n} (y)&={2|\Omega _0| \over T} \,\log n \, + F_0^n (y_{3} ) - y_{2} \, {2\pi |\Omega _0 | \over T^2} \, {\log n \over n} \,,\\&\quad + \frac{1}{n} F_1^n(y) \end{aligned} \end{aligned}$$

where $|\Omega _0|$ is the volume of the region enclosed by $\Sigma _0$. The functions $F_0^n$ and $F_1^n$ are smooth functions of their variables, which are uniformly bounded, together with their derivatives, as $n \rightarrow \infty$. The functions $F_0^n (y_3)$ and $F_1^n (y)$ satisfy the symmetries (1.9).

The second result relates $N_{\Sigma ^n}$ in (4.4) and $\tilde{N}_{\Sigma ^n_h}$ in (4.1).

### Lemma 4.2

Let $\alpha \in (0,1)$ and $h: \Sigma ^n \rightarrow {\mathbb {R}}$ be a small $C^{2, \alpha }$ function satisfying (1.9). Let $y \in \Sigma _0$ as in (4.2). Then

4.6 $$\begin{aligned} \begin{aligned} {\tilde{N}}_{ \Sigma _h^n} (y )&=N_{\Sigma ^n} (y) + \, \left( {2 \over T} \, \log n \, + b(y) \, \right) \,\,\int _{\Sigma _0 } h \\&\quad + \, \ell _1 [h] (y) + \, {1\over n} \ell _2 [h] (y) \quad {\text{ as } } n \rightarrow \infty . \end{aligned} \end{aligned}$$

In (4.6), $N_{\Sigma ^n} (y)$ is defined in (4.4) and *b*, $\ell _1$, $\ell _2$ are smooth functions, uniformly bounded, together with their derivatives, as $n \rightarrow \infty$, and they satisfy the symmetries (1.9). Besides, $\ell _i (0) = 0$, $\nabla _h \ell _i (0) \not = 0$, $i=1,2$, and if *h* is also even with respect to $y_2$ then $\ell _1[h]$ is even in $y_2$.

### Remark 4.1

The functions ${\tilde{N}}_{\Sigma _h^n} (y)$ as defined in (4.1) and $N_{\Sigma ^n} (y)$ in (4.4) satisfy the symmetries described in (1.9): they are even with respect to $y_1$ and $y_3$, and they are *T*-periodic in $y_3$.

Proposition 4.1 is a direct consequence of Lemmas 4.1 and 4.2.

The rest of the section is devoted to prove these results. We start with Lemma 4.1.

### Proof of Lemma 4.1.

Assume *y* satisfies (4.2). The expression of the non-local operator $N_{{\tilde{\Sigma }}^n}$ becomes simpler after the natural change of variables

4.7 $$\begin{aligned} {\tilde{x}}= X(x) = \Big ( x_1 \,, \, (R+x_2) \cos \left( \frac{ x_3}{ R} \right) , (R+x_2) \sin \left( \frac{ x_3}{ R} \right) \Big ) \end{aligned}$$

which has volume element $d{\tilde{x}} = (1+{x_2 \over R} ) \, dx$.

For the integrand, we check that

$$\begin{aligned} |{\tilde{x}} - X(y)|^2&= |{\bar{x}} - {\bar{y}}|^2 + a_R^2 \left( 1+{x_2 + y_{2} \over R} + {x_2 y_{2} \over R^2 }\right) \end{aligned}$$

where we use the notation ${\tilde{x}} = X (x)$ as in (4.7) and

4.8 $$\begin{aligned} x= ({\bar{x}}, x_3), \quad a_R^2= 2\, R^2 \, \left( 1-\cos \left( {x_3 - y_{3} \over R}\right) \right) . \end{aligned}$$

Hence,

$$\begin{aligned} N_{ \Sigma ^n} (y ) = \iiint \limits _{\Omega ^n}\frac{(1+{x_2 \over R})}{\sqrt{|{\bar{x}} - {\bar{y}}|^2 + a_R^2 \left( 1+{x_2 + y_{2} \over R} + {x_2 y_{2} \over R^2 }\right) }} \, \, dx. \end{aligned}$$

Since

$$\begin{aligned} \Omega ^n = \bigcup _{k=0}^{n-1} \Omega _k \end{aligned}$$

as in (1.6), we split the region of integration over the sets $\Omega _k$ and get

$$\begin{aligned} N_{ \Sigma ^n} (y )&= \iiint \limits _{\Omega ^n}\frac{(1+{x_2 \over R})}{\sqrt{|{\bar{x}} - {\bar{y}}|^2 + a_R^2 \left( 1+{x_2 + y_{2} \over R} + {x_2 y_{2} \over R^2 }\right) }} \, \, dx\\&= \sum _{k=0}^{n-1} \iiint \limits _{\Omega _k } \frac{(1+{x_2 \over R})}{\sqrt{|{\bar{x}} - {\bar{y}}|^2 + a_R^2 \left( 1+{x_2 + y_{2} \over R} + {x_2 y_{2} \over R^2 }\right) }} \, \, dx \\&= \sum _{k=0}^{n-1} I_k (y) \quad {\text{ where }} \quad a_R \quad \text{ is } \text{ in } (4.8), \text{ and } \\ I_k (y)&:=\iiint \limits _{\Omega _0} \frac{(1+{x_2 \over R})}{\sqrt{|{\bar{x}} - {\bar{y}}|^2 + a_{Rk}^2 \left( 1+{x_2 + y_{2} \over R} + {x_2 y_{2} \over R^2 }\right) }} \, \, dx \end{aligned}$$

with

$$\begin{aligned} a_{Rk} = 2\, R \, \sin \left( {k T \over 2R } + {x_{3} - y_{3} \over 2\, R} \right) . \end{aligned}$$

Recalling that $2 R \pi = n \, T$, we get that $a_{Rk} = {n T \over \pi } \sin ( {k \over n} \pi + {x_3 - y_{3} \over nT } \pi )$ and

$$\begin{aligned} I_k (y)&:={\pi \over n T} \iiint \limits _{\Omega _0} \frac{(1+{x_2 \over R})}{\sqrt{ \sin ^2 ( {k \over n} \pi + {x_3 - y_{3} \over nT } \pi ) \left( 1+{x_2 + y_{2} \over R} + {x_2 y_{2} \over R^2 }\right) + {|{\bar{x}} - {\bar{y}}|^2 \over n^2 T^2} \pi ^2}} \, \, dx \\&= {\pi \over n T} \iiint \limits _{\Omega _0} \frac{\delta }{\sqrt{ \sin ^2 ( {k \over n} \pi + {\alpha \over n } ) + \frac{\beta ^2}{n^2}}} \, \, dx, \end{aligned}$$

where, to simplify notations, we use

4.9 $$\begin{aligned} \begin{aligned} \alpha&= {x_3 - y_{3} \over T } \, \pi , \quad \beta ^2 = {|{\bar{x}} - {\bar{y}}|^2 \over T^2} \, \pi ^2 \, (1+ {x_2 + y_{2} \over R} + {x_2 y_{2} \over R^2 })^{-1} \\ \delta&= (1+{x_2 \over R}) (1+ {x_2 + y_{2} \over R} + {x_2 y_{2} \over R^2 })^{-{1\over 2}}. \end{aligned} \end{aligned}$$

Notice that $\alpha$, $\beta$ and $\delta$ do not depend on *k*. Also, we can expand this as follows:

4.10 $$\begin{aligned} \delta = 1 - \frac{y_{2}}{2R} + \frac{x_2}{2R} + O( \frac{1}{n^2} F_n(x,y) ). \end{aligned}$$

Thus,

$$\begin{aligned} N_{\Sigma ^n}&(y ) = {1\over T} \, \iiint \limits _{\Omega _0} \delta \, {\pi \over n} \, \left( \sum _{k=0}^{n-1} \frac{1}{\sqrt{ \sin ^2 ( {k \over n} \pi + {\alpha \over n } ) + \frac{\beta ^2}{n^2}}} \right) \, \, dx, \end{aligned}$$

which we write as

4.11 $$\begin{aligned} \begin{aligned} N_{ \Sigma ^n} (y )&= {1\over T} \, \iiint \limits _{\Omega _0} \delta \, \left( \int _0^\pi \frac{1}{\sqrt{ \sin ^2 ( s + {\alpha \over n } ) + \frac{\beta ^2}{n^2}}} \, ds \right) \, dx\\&\quad + {1\over T} \, \iiint \limits _{\Omega _0} \delta \, J (x; y)\, \, dx, \end{aligned} \end{aligned}$$

where

$$\begin{aligned} J (x; y)= \sum _{k=0}^{n-1} \int _{k\pi \over n}^{(k+1) \pi \over n} \left( \frac{1}{\sqrt{ \sin ^2 ( {k \over n} \pi + {\alpha \over n } ) + \frac{\beta ^2}{n^2}}} - \frac{1}{\sqrt{ \sin ^2 ( s + {\alpha \over n } ) + \frac{\beta ^2}{n^2}}} \right) \, ds \end{aligned}$$

We claim that

4.12 $$\begin{aligned} \begin{aligned} \int _0^\pi \frac{1}{\sqrt{ \sin ^2 ( s + {\alpha \over n } ) + \frac{\beta ^2}{n^2}}} \, ds&=2 \log n - \log |{\bar{x}}- {\bar{y}}|^2 + 2\pi \, {x_2 + y_{2} \over n\, T} \\&\quad + \log 4T^2 +2\int _0^{\pi \over 2} {t-\sin t \over t \sin t} \, {\text {d}}t + {\log n \over n^2} \, F_n (\alpha , \beta ) \end{aligned} \end{aligned}$$

and

4.13 $$\begin{aligned} \begin{aligned} J (x; y_0)&= F(\alpha , \beta ) + {1\over n} \, F_n (\alpha , \beta ), \end{aligned} \end{aligned}$$

where *F*, $F_n$ are uniformly bounded, as $n \rightarrow \infty$, together with their derivatives in $\alpha$ || $\beta$. The specific definition of $F_n$ changes from one formula to the other. We refer to (4.9) for $\alpha$ and $\beta$.

We assume for the moment the validity of (4.12) and (4.13), the proofs if which are postponed until later.

Using the expansion of $\delta$ as in (4.10) from (4.12) we get

4.14 $$\begin{aligned} \begin{aligned}&{1\over T} \, \iiint \limits _{\Omega _0} \delta \, \left( \int _0^\pi \frac{1}{\sqrt{ \sin ^2 ( s + {\alpha \over n } ) + \frac{\beta ^2}{n^2}}} \, ds \right) \, dx = {2 |\Omega _0| \over T} \, \log n \\&\quad \quad + {|\Omega _0 |\over T} \, A -{1\over T} \iiint \limits _{\Omega _0} \log (|{\bar{x}} - {\bar{y}}|^2 )\, dx \\&\quad \quad - {2\, \pi \, |\Omega _0| \over T^2} \, y_{2} \, {\log n \over n} \\&\quad \quad + {2\pi \over n T^2} \, |\Omega _0 | \, \left( 1-{A\over 2} + {1\over 2 |\Omega _0 |} \iiint \limits _{\Omega _0} \log (|{\bar{x}} - {\bar{y}}|^2 )\, dx \right) \, y_{2} \\&\quad \quad +{1\over n^2} F_n (y ) \\&\quad \quad {\text{ where }} \quad A= \left( \log 4T^2 +2\int _0^{\pi \over 2} {t-\sin t \over t \sin t} \, {\text {d}}t \right) . \end{aligned} \end{aligned}$$

In order to compute ${1\over T} \, \iiint \limits _{\Omega _0} \delta \, J (x; y)\, \, dx$, we need a more precise expansion of (4.13). From (4.9), we get

$$\begin{aligned} \beta&= {|{\bar{x}} - {\bar{y}}| \over T} \, \pi \, (1+ {x_2 + y_{2} \over R} + {x_2 y_{2} \over R^2 })^{-{1\over 2} } \\&={\bar{\beta }} \left( 1- {x_2 + y_{2} \over 2R} + O \left( {1\over n^2} \right) F_n (x,y)\right) \quad {\text{ where }} \quad {\bar{\beta }} = {|{\bar{x}} - {\bar{y}}| \over T} \, \pi . \end{aligned}$$

We thus write the function $F(\alpha , \beta )$ in (4.13) as follows

$$\begin{aligned} F(\alpha , \beta ) = F(\alpha , {\bar{\beta }}) -{\pi \over n T} \partial _\beta F (\alpha , {\bar{\beta }} ) [{\bar{\beta }} (x_2 + y_{2} )] + O\left( {1\over n^2}\right) F_n (x,y). \end{aligned}$$

Thus we conclude that

4.15 $$\begin{aligned} \begin{aligned}&{1\over T} \, \iiint \limits _{\Omega _0} \delta \, J (x; y)\, \, dx = {1\over T} \iiint \limits _{\Omega _0} F(\alpha , {\bar{\beta }} ) \, dx \\&\quad \quad - {\pi \over n T^2} \, y_{2} \, \left( \iiint \limits _{\Omega _0} F(\alpha , {\bar{\beta }}) \, dx + \iiint \limits _{\Omega _0} \partial _\beta F (\alpha , {\bar{\beta }} ) {\bar{\beta }} \, dx \right) \\&\quad \quad +{1\over n} F_n ({\bar{y}} ). \end{aligned} \end{aligned}$$

Observe now that the function ${1\over T} \iiint \limits _{\Omega _0} \log (|{\bar{x}} - {\bar{y}}|^2 )\, dx$ in (4.14) depends on $|\bar{y}|$ as consequence of the fact that $\Omega _0$ is invariant under rotation in the $(x_1, x_2)$-plane. Similarly, ${1\over T} \iiint \limits _{\Omega _0} F(\alpha , {\bar{\beta }} ) \, dx$ in (4.15) depends on $y_{3}$ and $|{\bar{y}}|$. Since points *y* on the boundary of $\Omega _0$ are functions of $y_3$, the expression ${1\over T} \iiint \limits _{\Omega _0} F(\alpha , {\bar{\beta }} ) \, dx$ only depends on $y_3$. The validity of the expansion (4.5) readily follows from (4.11), (4.14) and (4.15).

Next we prove the evenness of $N_{\Sigma ^n}$ as a function of $y_{3}$. Observe that

$$\begin{aligned} I_k ({\bar{y}}, - y_{3} )&=\iiint \limits _{\Omega _0} \frac{(1+{x_2 \over R})}{\sqrt{|{\bar{x}} - {\bar{y}}|^2 + \left( 2\, R \, \sin \left( {k T \over 2R } + {x_{3} + y_{3} \over 2\, R} \right) \right) ^2 \left( 1+{x_2 + y_{2} \over R} + {x_2 y_{2} \over R^2 }\right) }}\, dx \\ \\&\quad ( x_3= - z_3 , \quad {\bar{x}} = {\bar{z}} )\\&=\iiint \limits _{\Omega _0}\frac{1}{\sqrt{|{\bar{z}} - {\bar{y}}|^2 + \left( 2\, R \, \sin \left( {k T \over 2R } + {-z_{3} + y_{3} \over 2\, R} \right) \right) ^2 \left( 1+{y_2 + y_{2} \over R} + {y_2 y_{2} \over R^2 }\right) }} \, dz \\&= \iiint \limits _{\Omega _0}\frac{1}{\sqrt{|{\bar{x}} - {\bar{y}}|^2 + \left( 2\, R \, \sin \left( -{k \over n } \pi + {x_{3} - y_{3} \over 2\, R} \right) \right) ^2 \left( 1+{x_2 + y_{2} \over R} + {x_2 y_{2} \over R^2 }\right) }} \, dx\\&= \iiint \limits _{\Omega _0}\frac{1}{\sqrt{|{\bar{x}} - {\bar{y}}|^2 + \left( 2\, R \, \sin \left( {n-k \over n } \pi + {x_{3} - y_{3} \over 2\, R} \right) \right) ^2\left( 1+{x_2 + y_{2} \over R} + {x_2 y_{2} \over R^2 }\right) }} \, {\text {d}}y \\&= I_{n-k} ({\bar{y}} , y_{3}). \end{aligned}$$

Hence $N_{ \Sigma ^n} ({\bar{y}}, y_{3})$ is an even function in $y_{3}$, for $({\bar{y}}, y_{3}) \in \Sigma _0$, namely,

$$\begin{aligned} N_{ \Sigma ^n} ({\bar{y}}, - y_{3} )&= \sum _{k=0}^{n-1} I_k (-y_{03}) = \sum _{k=0}^{n-1} I_{n-k} (y_{3}) = \sum _{k=0}^{n-1} I_k (y_{3}) = N_{ \Sigma ^n} ({\bar{y}}, y_{3} ) \end{aligned}$$

as $I_0 ({\bar{y}}, y_{3}) = I_n ({\bar{y}}, y_{3}).$ This proves part of the statements in Remark 4.1. $\square$

The rest of the proof is devoted to obtaining (4.12) and (4.13).

### Proof of (4.12)

We observe that

$$\begin{aligned} \int _0^\pi \frac{1}{\sqrt{ \sin ^2 ( s + {\alpha \over n } ) + \frac{\beta ^2}{n^2}}} \, ds&= ( \int _0^{\pi \over 2} + \int _{\pi \over 2}^\pi ) \frac{1}{\sqrt{ \sin ^2 ( s + {\alpha \over n } ) + \frac{\beta ^2}{n^2}}} \, ds \\&= \int _0^{\pi \over 2} \frac{1}{\sqrt{ \sin ^2 ( s + {\alpha \over n } ) + \frac{\beta ^2}{n^2}}} \, ds \\  &\quad + \int _0^{\pi \over 2} \frac{1}{\sqrt{ \sin ^2 ( s - {\alpha \over n } ) + \frac{\beta ^2}{n^2}}} \, ds, \end{aligned}$$

since

$$\begin{aligned}&\int _{\pi \over 2}^\pi \frac{1}{\sqrt{ \sin ^2 ( s + {\alpha \over n } ) + \frac{\beta ^2}{n^2}}} \, ds = \quad (s=\pi - t ) \\&\quad = -\int _{\pi \over 2}^0 \frac{1}{\sqrt{ \sin ^2 ( \pi -t + {\alpha \over n } ) + \frac{\beta ^2}{n^2}}} \, {\text {d}}t= \int _0^{\pi \over 2} \frac{1}{\sqrt{ \sin ^2 ( s - {\alpha \over n } ) + \frac{\beta ^2}{n^2}}} \, ds. \end{aligned}$$

Thus, let us consider that

$$\begin{aligned}&\int _0^{\pi \over 2} \frac{1}{\sqrt{ \sin ^2 ( s + {\alpha \over n } ) + \frac{\beta ^2}{n^2}}} \, ds =\int _0^{\pi \over 2} ( \frac{1}{\sqrt{ \sin ^2 ( s + {\alpha \over n } ) + \frac{\beta ^2}{n^2}}} \, - \frac{1}{\sqrt{ ( s + {\alpha \over n } )^2 + \frac{\beta ^2}{n^2}}} ) \, ds \\&\quad \quad + \log \left( {n\pi +\alpha \over \beta } + \sqrt{ ({n\pi +\alpha \over \beta } )^2+1}\right) - \log \left( { \alpha \over \beta } + \sqrt{ ({\alpha \over \beta } )^2+1}\right) \\&\quad = \int _0^{\pi \over 2} ( \frac{1}{\sqrt{ \sin ^2 ( s + {\alpha \over n } ) + \frac{\beta ^2}{n^2}}} \, - \frac{1}{\sqrt{ ( s + {\alpha \over n } )^2 + \frac{\beta ^2}{n^2}}} ) \, ds \\&\quad \quad +\log n + \log {2\pi \over \alpha + \sqrt{\alpha ^2 + \beta ^2} } + \log (1+ {\alpha \over n \pi } ) + \log \left( {1+ \sqrt{1+{\beta ^2 \over (n\pi + \alpha )^2} }\over 2} \right) . \end{aligned}$$

Furthermore,

$$\begin{aligned}&\int _0^{\pi \over 2} ( \frac{1}{\sqrt{ \sin ^2 ( s + {\alpha \over n } ) + \frac{\beta ^2}{n^2}}} \, - \frac{1}{\sqrt{ ( s + {\alpha \over n } )^2 + \frac{\beta ^2}{n^2}}} ) \, ds\\&\quad = \int _0^{\pi \over 2} \frac{( s + {\alpha \over n } )^2 - \sin ^2 ( s + {\alpha \over n } ) }{\sqrt{ \sin ^2 ( s + {\alpha \over n } ) + \frac{\beta ^2}{n^2}} \sqrt{ ( s + {\alpha \over n } )^2 + \frac{\beta ^2}{n^2}} \left( \sqrt{ \sin ^2 ( s + {\alpha \over n } ) + \frac{\beta ^2}{n^2}} + \sqrt{ ( s + {\alpha \over n } )^2 + \frac{\beta ^2}{n^2}} \right) } \, ds \\  &\quad = (t= s+ {\alpha \over n})\\&\quad = \int _{\alpha \over n}^{{\pi \over 2} + {\alpha \over n} } \frac{t^2 - \sin ^2 t }{\sqrt{ \sin ^2 t + \frac{\beta ^2}{n^2}} \sqrt{ t^2 + \frac{\beta ^2}{n^2}} \left( \sqrt{ \sin ^2 t + \frac{\beta ^2}{n^2}} + \sqrt{ t^2 + \frac{\beta ^2}{n^2}} \right) } \, {\text {d}}t \\&\quad = (\int _{C \over n}^{{\pi \over 2} + {\alpha \over n} } + \int _{\alpha \over n}^{{C \over n} } ) f(t, {\beta ^2 \over n^2} )\, {\text {d}}t \end{aligned}$$

for some fixed $C>0$, where

$$\begin{aligned} f(t, \theta ) = \frac{t^2 - \sin ^2 t }{\sqrt{ \sin ^2 t + \theta } \sqrt{ t^2 + \theta } \left( \sqrt{ \sin ^2 t + \theta } + \sqrt{ t^2 + \theta } \right) }. \end{aligned}$$

Since $\partial _\theta f(t,0) \sim {1\over t}$, as $t \rightarrow 0$, we have

$$\begin{aligned} \int _{C \over n}^{{\pi \over 2} + {\alpha \over n} } f(t, {\beta ^2 \over n^2} )\, {\text {d}}t&= \int _{C \over n}^{{\pi \over 2} + {\alpha \over n} } f(t, 0)\, {\text {d}}t + \log n \, O({\beta ^2 \over n^2})\\&= \int _0^{\pi \over 2} {t-\sin t \over t \sin t} \, {\text {d}}t + \log n \, O({\beta ^2 \over n^2}) \end{aligned}$$

Moreover, we check directly that $\int _{C \over n}^{{\pi \over 2} + {\alpha \over n} } f(t, {\beta ^2 \over n^2} )\, {\text {d}}t = O({1\over n^2})$ and we conclude that

$$\begin{aligned} \int _0^{\pi \over 2} \frac{1}{\sqrt{ \sin ^2 ( s + {\alpha \over n } ) + \frac{\beta ^2}{n^2}}} \, ds&= \log n + \log {2\pi \over \alpha + \sqrt{\alpha ^2 + \beta ^2} }+ \int _0^{\pi \over 2} {t-\sin t \over t \sin t} \, {\text {d}}t \\&\quad + \log (1+ {\alpha \over n \pi } ) + \log n \, O({\beta ^2 \over n^2}), \end{aligned}$$

and hence,

$$\begin{aligned} \int _0^{\pi } \frac{1}{\sqrt{ \sin ^2 ( s + {\alpha \over n } ) + \frac{\beta ^2}{n^2}}} \, ds&= 2\log n + \log {2\pi \over \alpha + \sqrt{\alpha ^2 + \beta ^2} }+ \int _0^{\pi \over 2} {t-\sin t \over t \sin t} \, {\text {d}}t \\&\quad + \log (1+ {\alpha \over n \pi } ) + \log n \, O({\beta ^2 \over n^2})\\&\quad + \log {2\pi \over -\alpha + \sqrt{\alpha ^2 + \beta ^2} }+ \int _0^{\pi \over 2} {t-\sin t \over t \sin t} \, {\text {d}}t \\&\quad + \log (1+ {-\alpha \over n \pi } ) + \log n \, O({\beta ^2 \over n^2}), \end{aligned}$$

which, in combination with (4.9), gives the expansion (4.12). $\square$

### Proof of (4.13)

To estimate *J*, we start rename $k= n-k-1$ and change the variable of integration $t=\pi -s$ to get

$$\begin{aligned} J&= \sum _{k=0}^{n-1} \int _{(n-k-1)\pi \over n}^{(n-k) \pi \over n} \left( \frac{1}{\sqrt{ \sin ^2 ( {n-k-1 \over n} \pi + {\alpha \over n } ) + \frac{\beta ^2}{n^2}}} - \frac{1}{\sqrt{ \sin ^2 ( s + {\alpha \over n } ) + \frac{\beta ^2}{n^2}}} \right) \, ds \\&=\sum _{k=0}^{n-1} \int _{(n-k-1)\pi \over n}^{(n-k) \pi \over n} \left( \frac{1}{\sqrt{ \sin ^2 ( {k+1 \over n} \pi - {\alpha \over n } ) + \frac{\beta ^2}{n^2}}} - \frac{1}{\sqrt{ \sin ^2 ( s + {\alpha \over n } ) + \frac{\beta ^2}{n^2}}} \right) \, ds\\&= \sum _{k=0}^{n-1} \int _{k\pi \over n}^{(k+1) \pi \over n} \left( \frac{1}{\sqrt{ \sin ^2 ( {k+1 \over n} \pi - {\alpha \over n } ) + \frac{\beta ^2}{n^2}}} - \frac{1}{\sqrt{ \sin ^2 ( t - {\alpha \over n } ) + \frac{\beta ^2}{n^2}}} \right) \, {\text {d}}t. \end{aligned}$$

Combining this expression for *J* with its definition we get

$$\begin{aligned} 2&J= \sum _{k=0}^{n-1} (a_k + b_k ) \, , \quad {\text{ where }} \\ a_k&= \int _{k\pi \over n}^{(k+1) \pi \over n} \left( \frac{1}{\sqrt{ \sin ^2 ( {k \over n} \pi + {\alpha \over n } ) + \frac{\beta ^2}{n^2}}} - \frac{1}{\sqrt{ \sin ^2 ( s + {\alpha \over n } ) + \frac{\beta ^2}{n^2}}} \right) \, ds \\ b_k&= \int _{k\pi \over n}^{(k+1) \pi \over n} \left( \frac{1}{\sqrt{ \sin ^2 ( {k+1 \over n} \pi - {\alpha \over n } ) + \frac{\beta ^2}{n^2}}} - \frac{1}{\sqrt{ \sin ^2 ( s - {\alpha \over n } ) + \frac{\beta ^2}{n^2}}} \right) \, ds. \end{aligned}$$

Using the change of variables $s={t\over n} +{k\pi \over n}$, we get

$$\begin{aligned} a_k&= \int _0^{\pi } \left( \frac{1}{\sqrt{ n^2 \sin ^2 ( {k \pi + \alpha \over n} ) + \beta ^2} }- \frac{1}{\sqrt{ n^2 \sin ^2 ( {t+ k \pi + \alpha \over n } ) + \beta ^2}}\right) \, {\text {d}}t. \end{aligned}$$

Using the change of variables ${t\over n} ={k+1 \over n} \pi - s$,

$$\begin{aligned} b_k&= \int _0^{\pi } \left( \frac{1}{\sqrt{ n^2 \sin ^2 ( {(k +1) \pi - \alpha \over n} ) + \beta ^2} }- \frac{1}{\sqrt{ n^2 \sin ^2 ( -{t\over n} + {(k+1) \pi -\alpha \over n } ) + \beta ^2}}\right) \, {\text {d}}t. \end{aligned}$$

Thus we have

$$\begin{aligned} 2J&= \sum _{k=0}^{n-1} \int _0^{\pi } \left( \frac{1}{\sqrt{ n^2 \sin ^2 ( {k \pi + \alpha \over n} ) + \beta ^2} }- \frac{1}{\sqrt{ n^2 \sin ^2 ( {s+ k \pi + \alpha \over n } ) + \beta ^2}}\right) \, ds \\&\quad + \sum _{k=0}^{n-1} \int _0^{\pi } \left( \frac{1}{\sqrt{ n^2 \sin ^2 ( {(k +1) \pi - \alpha \over n} ) + \beta ^2} }- \frac{1}{\sqrt{ n^2 \sin ^2 ( {-s+ (k+1) \pi -\alpha \over n } ) + \beta ^2}}\right) \, ds. \end{aligned}$$

Let us decompose

$$\begin{aligned} 2J&= \sum _{k=0}^{[\sqrt{n}]-1} + \sum _{k=[\sqrt{n}]}^{n-1 - [\sqrt{n}]} + \sum _{k=n - [\sqrt{n}]}^{n-1} = J_1 + J_2 + J_3. \end{aligned}$$

We claim that

4.16 $$\begin{aligned} J_1 = J_3. \end{aligned}$$

Indeed, we check they have the same number of elements which can be redistributed as

4.17 $$\begin{aligned} J_1 = \sum _{k=0}^{[\sqrt{n}]-1} (a_k + b_k) = \sum _{k=n-[\sqrt{n}]}^{n-1} (a_{n-k-1} + b_{n-k-1} ), \end{aligned}$$

where

$$\begin{aligned}&a_{n-k-1} + b_{n-k-1}\\&\quad = \int _0^{\pi } \left( \frac{1}{\sqrt{ n^2 \sin ^2 ( {(n-k-1 )\pi + \alpha \over n} ) + \beta ^2} }- \frac{1}{\sqrt{ n^2 \sin ^2 ( {s+ (n-k-1) \pi + \alpha \over n } ) + \beta ^2}}\right) \, ds \\&\quad \quad + \int _0^{\pi } \left( \frac{1}{\sqrt{ n^2 \sin ^2 ( {(n-k ) \pi - \alpha \over n} ) + \beta ^2} }- \frac{1}{\sqrt{ n^2 \sin ^2 ( {-s+ (n-k) \pi -\alpha \over n } ) + \beta ^2}}\right) \, ds \\&\quad = \int _0^{\pi } \left( \frac{1}{\sqrt{ n^2 \sin ^2 ( {(k+1 )\pi - \alpha \over n} ) + \beta ^2} }- \frac{1}{\sqrt{ n^2 \sin ^2 ( {s \pi - k \pi + \alpha \over n } ) + \beta ^2}}\right) \, ds \\&\quad \quad + \int _0^{\pi } \left( \frac{1}{\sqrt{ n^2 \sin ^2 ( {k \pi + \alpha \over n} ) + \beta ^2} }- \frac{1}{\sqrt{ n^2 \sin ^2 ( {-s-k \pi -\alpha \over n } ) + \beta ^2}}\right) \, ds \\&\quad = \int _0^{\pi } \left( \frac{1}{\sqrt{ n^2 \sin ^2 ( {(k+1 )\pi - \alpha \over n} ) + \beta ^2} }- \frac{1}{\sqrt{ n^2 \sin ^2 ( {s \pi - k \pi + \alpha \over n } ) + \beta ^2}}\right) \, ds \\&\quad \quad + \int _0^{\pi } \left( \frac{1}{\sqrt{ n^2 \sin ^2 ( {k \pi + \alpha \over n} ) + \beta ^2} }- \frac{1}{\sqrt{ n^2 \sin ^2 ( {s+k \pi +\alpha \over n } ) + \beta ^2}}\right) \, ds . \end{aligned}$$

Observe now that the second term of the last equality can be written as

$$\begin{aligned}&\int _0^\pi \frac{1}{\sqrt{ n^2 \sin ^2 ( {s-\pi - k \pi + \alpha \over n } ) + \beta ^2}} ds = \quad (s-\pi = -t)\\&\quad = \int _0^\pi \frac{1}{\sqrt{ n^2 \sin ^2 ( {t +k \pi - \alpha \over n } ) + \beta ^2}} {\text {d}}t. \end{aligned}$$

Thus we conclude that

$$\begin{aligned} a_{n-k-1} + b_{n-k-1} = a_k + b_k. \end{aligned}$$

Inserting this identity in (4.17), we conclude that $J_1=J_3$.

Let us analyze the term $J_1$. Thus we are assuming $0\le k \le [\sqrt{n}]$.

For $0\le k \le [\sqrt{n}]$, we Taylor expand the first integrand in the definition of $a_k$

$$\begin{aligned}&\left( n^2 \sin ^2 ( {k \pi + \alpha \over n} ) + \beta ^2 \right) ^{-{1\over 2}}= \left( (k\pi + \alpha )^2 (1-{1\over 3} ({k\pi +\alpha \over n})^2 + O({k\over n})^4 ) +\beta ^2 \right) ^{-{1\over 2}}\\&\quad = \left( 1+{1\over 6} ({k\pi +\alpha \over n})^2 + O({k\over n})^4 \right) \left( (k\pi + \alpha )^2 +{\beta ^2 \over (1-{1\over 3} ({k\pi +\alpha \over n})^2 + O({k\over n})^4 ) } \right) ^{-{1\over 2}} \\&\quad = \left( 1+{1\over 6} ({k\pi +\alpha \over n})^2 + O({k\over n})^4 \right) \left( (k\pi + \alpha )^2 + \beta ^2 \right) ^{-{1\over 2} } \\&\quad \quad \times \left( 1- {\beta ^2 \over (k\pi + \alpha )^2 + \beta } ({1\over 6} ({k\pi +\alpha \over n})^2 + O({k\over n})^4) ) \right) \\&\quad = \left( 1+{1\over 6} ({k\pi +\alpha \over n})^2 + O\left( {1\over n^2}\right) \right) \left( (k\pi + \alpha )^2 + \beta ^2\right) ^{-{1\over 2} }\\&\quad = \left( 1+{1\over 6} {k^2\pi ^2 \over n^2} \right) \left( (k\pi + \alpha )^2 + \beta ^2\right) ^{-{1\over 2} } \\&\qquad + \left( O({k\over n^2}) + O\left( {1\over n^2}\right) \right) \left( (k\pi + \alpha )^2 + \beta ^2\right) ^{-{1\over 2} } . \end{aligned}$$

Similarly we get, for $t \in (0,\pi )$, that

$$\begin{aligned} \left( n^2 \sin ^2 ( {t+k \pi + \alpha \over n} ) + \beta ^2 \right) ^{-{1\over 2}}&= \left( 1+{1\over 6} {k^2\pi ^2 \over n^2} \right) \left( (t+ k\pi + \alpha )^2 + \beta ^2 \right) ^{-{1\over 2} } \\&\quad + \left( O({k\over n^2}) + O\left( {1\over n^2}\right) \right) \left( (t+ k\pi + \alpha )^2 + \beta ^2\right) ^{-{1\over 2} } . \end{aligned}$$

Therefore, setting

4.18 $$\begin{aligned} \ell (s) = \sqrt{ (s+k\pi )^2 + \beta ^2}, \end{aligned}$$

we get

$$\begin{aligned} a_k&= {1+{1\over 6} {k^2\pi ^2 \over n^2} \over \ell (\alpha ) } \int _0^\pi {s^2 + 2s (k\pi + \alpha ) \over \ell (s+\alpha ) [ \ell (\alpha ) + \ell (s+\alpha ) ]} ds \\&\quad + \left( O({k\over n^2}) + O\left( {1\over n^2}\right) \right) \left( \int _0^\pi {ds \over \ell (\alpha ) }\, + \int _0^\pi {ds \over \ell (\alpha +s) }\, \right) \end{aligned}$$

and

$$\begin{aligned} b_k&= {1+{1\over 6} {k^2\pi ^2 \over n^2} \over \ell ( \pi - \alpha ) } \int _0^\pi {s^2 - 2s ( \pi - \alpha ) \over \ell ( \pi - \alpha - s) [ \ell ( \pi - \alpha ) + \ell ( \pi - \alpha -s) ] } ds \\&\quad + \left( O({k\over n^2}) + O\left( {1\over n^2}\right) \right) \left( \int _0^\pi {ds \over \ell (\pi -\alpha ) \, } + \int _0^\pi {ds \over \ell (\pi -\alpha - s )} \, \right) . \end{aligned}$$

The above expressions can be further expanded to

$$\begin{aligned} a_k&= {1\over 2 \pi ^2 k^2} \left( 1+{1\over 6} {k^2\pi ^2 \over n^2} \right) \int _0^\pi 2s\, ds \\&\quad + (1+{1\over 6} {k^2\pi ^2 \over n^2}) \int _0^\pi {2 s \, \over \sqrt{ (1 + {\alpha \over k \pi } )^2 + {\beta ^2 \over (k\pi )^2} }\, \ell (s+\alpha ) [ \ell (\alpha ) + \ell (s+\alpha )] } -{s \over \pi ^2 k^2}\, ds \\&\quad + {1+{1\over 6} {k^2\pi ^2 \over n^2} \over \sqrt{ (k\pi + \alpha )^2 + \beta ^2 } }\int _0^\pi {s^2 +2s\alpha \over \ell (s+\alpha ) [ \ell (\alpha ) + \ell (s+\alpha )] } ds \\&\quad + \left( O({k\over n^2}) + O\left( {1\over n^2}\right) \right) \left( \int _0^\pi \frac{1}{\ell (\alpha ) } \, ds + \int _0^\pi \frac{ds}{\ell (\alpha +s )} \right) ,\\ b_k&= - {1\over 2\pi ^2 k^2} \left( 1+{1\over 6} {k^2\pi ^2 \over n^2} \right) \int _0^\pi 2s\, ds \\&\quad + ( 1+{1\over 6} {k^2\pi ^2 \over n^2}) \int _0^\pi {-2 s \, \over \sqrt{ (1 + {\pi - \alpha \over k \pi } )^2 + {\beta ^2 \over (k\pi )^2} } \, \ell (\pi - \alpha - s) [ \ell (\pi - \alpha ) + \ell (\pi - \alpha -s) ] } \\&\quad +{s \over \pi ^2 k^2} \, ds \\&\quad + {1+{1\over 6} {k^2\pi ^2 \over n^2} \over \sqrt{ ((k+1) \pi -\alpha )^2 + \beta ^2 } }\int _0^\pi {s^2 - 2s (\pi - \alpha ) \over \ell (\pi - \alpha - s) [ \ell (\pi - \alpha ) + \ell (\pi - \alpha -s) ] } ds \\&\quad + \left( O({k\over n^2}) + O\left( {1\over n^2}\right) \right) \left( \int _0^\pi \frac{1}{\ell (\pi -\alpha ) } + \int _0^\pi \frac{1}{\ell (\pi -\alpha - s ) } \, ds \right) \end{aligned}$$

so that

$$\begin{aligned} a_k + b_k&= i + ii + iii + iv \\&\quad + \left( O({k\over n^2}) + O\left( {1\over n^2}\right) \right) \left( \int _0^\pi {ds \over \ell (\alpha )} \, + \int _0^\pi {ds \over \ell (\alpha +s ) } \, \right) ,\\&\quad + \left( O({k\over n^2}) + O\left( {1\over n^2}\right) \right) \left( \int _0^\pi {ds \over \ell (\pi -\alpha ) }\, + \int _0^\pi {ds \over \ell (\pi -\alpha - s ) }\, \right) , \quad {\text{ with }} \\ i&=(1+{1\over 6} {k^2\pi ^2 \over n^2}) \int _0^\pi {2 s \, \over \sqrt{ (1 + {\alpha \over k \pi } )^2 + {\beta ^2 \over (k\pi )^2}}\, \ell (s+\alpha ) [ \ell (\alpha ) + \ell (s+\alpha )] } -{ s \over \pi ^2 k^2} \, ds \\ ii&=( 1+{1\over 6} {k^2\pi ^2 \over n^2}) \int _0^\pi {-2 s \, \over \sqrt{ (1 + {\pi - \alpha \over k \pi } )^2 + {\beta ^2 \over (k\pi )^2} } \, \ell (\pi - \alpha - s) [ \ell (\pi - \alpha ) + \ell (\pi - \alpha -s) ] }\\&\quad +{s \over \pi ^2 k^2} \, ds \\ iii&= {1+{1\over 6} {k^2\pi ^2 \over n^2} \over \sqrt{ (k\pi + \alpha )^2 + \beta ^2 } }\int _0^\pi {s^2 +2s\alpha \over \ell (s+\alpha ) [ \ell (\alpha ) + \ell (s+\alpha )] } ds \\ iv&= {1+{1\over 6} {k^2\pi ^2 \over n^2} \over \sqrt{ ( \pi - \alpha )^2 + \beta ^2 } }\int _0^\pi {s^2 - 2s (\pi - \alpha ) \over \ell (\pi - \alpha - s) [ \ell (\pi - \alpha ) + \ell (\pi - \alpha -s) ] } ds. \end{aligned}$$

Expanding $\ell$ given by (4.18) as *k* becomes large, we compute that

$$\begin{aligned} i&= -{3\over 2 \pi ^3 k^3} \int _0^\pi (2\alpha + s ) \, ds +{1\over k^4} F_k (\alpha , \beta ) \\ ii&= {1\over \pi ^3 k^3} \int _0^\pi (s^2 + 2 \alpha s ) \, ds +{1\over k^4} F_k (\alpha , \beta ) \\ iii&= {3\over 2 \pi ^3 k^3} \int _0^\pi (2\pi -2\alpha - s ) \, ds +{1\over k^4} F_k (\alpha , \beta ) \\ iv&= {1\over \pi ^3 k^3} \int _0^\pi (s^2 - 2s (\pi - \alpha ) ) \, ds +{1\over k^4} F_k (\alpha , \beta ), \end{aligned}$$

where $F_k$ denotes a generic function, whose precise definition changes from line to line, with the property that it is bounded, together with its derivatives $\partial _\alpha F_k$, $\partial _\beta F_k$. We check that

$$\begin{aligned}&-{3\over 2 \pi ^3 } \int _0^\pi (2\alpha + s ) \, ds + {1\over \pi ^3 } \int _0^\pi (s^2 + 2 \alpha s ) \, ds \\&\quad \quad +{3\over 2 \pi ^3 } \int _0^\pi (2\pi -2\alpha - s ) \, ds + {1\over \pi ^3 } = ({1\over 6} - {\alpha \over 2\pi }). \end{aligned}$$

We conclude that

4.19 $$\begin{aligned} \begin{aligned} J_1&= F(\alpha , \beta ) + {1\over n} \, F_n (\alpha , \beta ), \end{aligned} \end{aligned}$$

where *F*, $F_n$ are uniformly bounded, as $n \rightarrow \infty$, together with their derivatives in $\alpha$ || $\beta$.

We now consider

$$\begin{aligned} J_2 = \sum _{k=[\sqrt{n}]}^{n-1-[\sqrt{n}]} a_k + b_k. \end{aligned}$$

We write

$$\begin{aligned} J_2&= {1\over n} \sum _{k=[\sqrt{n}]}^{n-1-[\sqrt{n}]} \int _0^\pi \left( f({\alpha \over n}) - f ({\alpha + s \over n}) + f({\pi -\alpha \over n}) - f ({\pi +s -\alpha \over n}) \right) \, ds\\&\quad {\text{ with }} \\ f(\theta )&= {1 \over \sqrt{ \sin ^2 ({k\pi \over n} + \theta )+{\beta ^2 \over n^2}}}. \end{aligned}$$

We have

$$\begin{aligned} \sqrt{ \sin ^2 ({k\pi \over n} + {\theta \over n} ) +{\beta ^2 \over n^2} }&= \sin ({k \pi \over n}) \left( (\cos {\theta \over n} + \cot {k\pi \over n} \sin {\theta \over n} )^2 +{\beta ^2 \over n^2 \, \sin ^2 ({k \pi \over n})} \right) ^{1\over 2} \\&= \sin ({k \pi \over n}) \left( 1+ 2 {\theta \over n} \cot {k\pi \over n} + O({1\over n^2}) \right) ^{1\over 2}\\&= \sin ({k \pi \over n}) \left( 1+ {\theta \over n} \cot {k\pi \over n} + O({1\over n^2}) \right) . \end{aligned}$$

Hence,

$$\begin{aligned} J_2&= {1\over n} \sum _{k=[\sqrt{n}]}^{n-1-[\sqrt{n}]} {\cot {k\pi \over n} \over \sin {k\pi \over n}} \int _0^\pi \left( {2s \over n} + O({1\over n^2}) \right) \, ds \\&= {1\over n^2} \sum _{k=[\sqrt{n}]}^{n-1-[\sqrt{n}]} {\cot {k\pi \over n} \over \sin {k\pi \over n}} \left( \pi ^2 + O({1\over n}) \right) \\&= {1\over n^2} \sum _{k=[\sqrt{n}]}^{n-1-[\sqrt{n}]} {\cos {k\pi \over n} \over \sin ^2 {k\pi \over n}} \left( \pi ^2 + O({1\over n}) \right) . \end{aligned}$$

Relabelling *k* with $n-k$, we get

$$\begin{aligned} \sum _{k=[\sqrt{n}]}^{n-1-[\sqrt{n}]}&{\cos {k\pi \over n} \over \sin ^2 {k\pi \over n}} = \sum _{k=[\sqrt{n}]+1}^{n-[\sqrt{n}]} {\cos {(n-k) \pi \over n} \over \sin ^2 {(n-k)\pi \over n}} \\&= - \sum _{k=[\sqrt{n}]+1}^{n-[\sqrt{n}]} {\cos {k \pi \over n} \over \sin ^2 {k \pi \over n}}\\&= - \sum _{k=[\sqrt{n}]}^{n-1-[\sqrt{n}]} {\cos {k \pi \over n} \over \sin ^2 {k \pi \over n}} + {\cos {[\sqrt{n}] \over n} \pi \over \sin ^2 {[\sqrt{n}] \over n} \pi } - {\cos {n- [\sqrt{n}] \over n} \pi \over \sin ^2 {n-[\sqrt{n}] \over n} \pi } \\&= - \sum _{k=[\sqrt{n}]}^{n-1-[\sqrt{n}]} {\cos {k \pi \over n} \over \sin ^2 {k \pi \over n}} + {2\cos {[\sqrt{n}] \over n} \pi \over \sin ^2 {[\sqrt{n}] \over n} \pi } . \end{aligned}$$

Thus

$$\begin{aligned} \sum _{k=[\sqrt{n}]}^{n-1-[\sqrt{n}]} {\cos {k \pi \over n} \over \sin ^2 {k \pi \over n}}&= {\cos {[\sqrt{n}] \over n} \pi \over \sin ^2 {[\sqrt{n}] \over n} \pi } , \quad {\text{ and }} \end{aligned}$$

4.20 $$\begin{aligned} J_2= {\pi ^2 \over n^2} \, {\cos {[\sqrt{n}] \over n} \pi \over \sin ^2 {[\sqrt{n}] \over n} \pi } \, (1+ O({1\over n})) = {\pi ^2 \over n} \, \, (1+ O({1\over n})). \end{aligned}$$

Putting together (4.16), (4.19) and (4.20), we obtain (4.13). $\square$

We next prove Lemma 4.2.

### Proof of Lemma 4.2.

We write that

$$\begin{aligned} {\tilde{N}}_{\Sigma ^n_h} (y)&= N_1[h] (y) + N_2[h] (y)\\ N_1[h] (y)&= \int _{{\tilde{\Omega }}^n_h }\dfrac{d {\tilde{x}}}{|X(y) -{\tilde{x}}|}\\ N_2[h] (y)&= \int _{{\tilde{\Omega }}^n_h } \left( \dfrac{1}{|X(y_h) -{\tilde{x}}|} - \dfrac{1}{|X(y) -{\tilde{x}}|}\right) d {\tilde{x}}. \end{aligned}$$

Here we use the same notations as in (4.1).

We start with $N_2$. Thus we readily get

$$\begin{aligned} N_2 [0] (y) = 0. \end{aligned}$$

Arguing as in the proof of Lemma 4.1, we can show that there exists a constant $C>0$ such that

$$\begin{aligned} | N_2 [h] (y)| \le C \, \left( \sum _{j=1}^n {1\over j^2} \right) \, \Vert h \Vert _{L^\infty } \end{aligned}$$

for all *n* large. Assume now that *h* is even in $y_2$. We aim at showing that $N_2$ is the sum of a term which is even in $y_2$ plus another term which is $n^{-1}$ smaller, as in (4.6). We start with the change of variable ${\tilde{x}} = X (x)$ as in (4.7), we have

$$\begin{aligned} N_2[h] (y)&= \int _{ \Omega ^n_h } \left( \dfrac{1}{|X(y_h) -X( x)|} - \dfrac{1}{|X(y) - X(x)|}\right) (1+ {x_2 \over R}) d x \end{aligned}$$

and we just consider $\int _{ \Omega ^n_h } \left( \dfrac{1}{|X(y_h) -X( x)|} - \dfrac{1}{|X(y) - X(x)|}\right) dx$, as the other part is $n^{-1}$-smaller for *n* large.

Writing

$$\begin{aligned} X(y_h)&= X(y) + T[h] (y), \quad T[h] (y) = h (y) \int _0^1 DX (y+ s h \nu ) \, [\nu ]\, ds, \end{aligned}$$

we have that

$$\begin{aligned} X( y )&= (y_1 , -y_2+R , y_3) + O(R^{-1})\\ X( y) - X(x)&= (y_1 - x_1 , y_2 - x_2 , y_3 - x_3 ) + O(R^{-1})\\ T[h] ( y)&= h(y) \nu ( y) + O(R^{-1}). \end{aligned}$$

Hence

$$\begin{aligned} N_2[h] (y )&= {\bar{N}}_2[h] (y) + {1\over n} \ell _2 [h] (y)\\ {\bar{N}}_2[h](y)&= \int _{ \Omega ^n_h } |y - x|^{-1} \left[ \left( 1+ 2 {h \nu (y) \cdot (y - x) \over |y - x|^2} + { h^2 \over |y - x|^2} \right) ^{-{1\over 2}} -1 \right] , \end{aligned}$$

with $\ell _2 [0] =0$ and $|\ell _2 [h] (y) | \le C \Vert h \Vert _{L^\infty }$ for some constant *C*. Using the symmetries of the domain $\Omega _h^n$, we can show that

$$\begin{aligned} {\bar{N}}_2 [h] ({\hat{y}} ) = {\bar{N}}_2 [h] (y), \quad {\hat{y}} = (y_1, - y_2, y_3), \end{aligned}$$

since *h* is assumed to be even in $y_2$.

Let us now consider $N_1 [h] (y)$. We decompose the domain ${\tilde{\Omega }}^n_h$ into

$$\begin{aligned} {\tilde{\Omega }}^n_h = {\tilde{\Omega }}^n \cup \left( {\tilde{\Omega }}^n_h \setminus {\tilde{\Omega }}^n \right) , \end{aligned}$$

and we split

$$\begin{aligned} N_1[h] (y)&= N_{\Sigma ^n} (y)+ {\bar{N}}_1 [h] (y),\\ {\bar{N}}_1 [h] (y)&= \int _{{\tilde{\Omega }}^n_h \setminus {\tilde{\Omega }}^n }\dfrac{d {\tilde{x}}}{|X(y) -{\tilde{x}}|}. \end{aligned}$$

We refer to (4.4) for the definition of $N_{\Sigma ^n} (y)$.

As in the prof of Lemma 4.1, we change variable ${\tilde{x}} = X(x)$, with $x \in \Omega _h^n \setminus \Omega ^n$ to get

$$\begin{aligned} {\bar{N}}_1 [h] (y)= \int _{ \Omega ^n_h \setminus \Omega ^n }\dfrac{ (1+ {x_2 \over R} ) \, d x}{|X(y) -X(x)|}, \end{aligned}$$

since $d {\tilde{x}} =(1+ {x_2 \over R} ) \, d x$. We perform a second change of variables in $\Omega ^n_h \setminus \Omega ^n$,

$$\begin{aligned} x = z + s h (z ) \, \nu (z), \quad z \in \Sigma ^n, \quad s \in (0,1). \end{aligned}$$

Thus

$$\begin{aligned} {\bar{N}}_1 [h] (y)&= \int _{\Sigma ^n } h(z ) \, d \sigma (z) \, \int _0^1 \dfrac{ (1+ {(z + s h (z ) \, \nu (z))_2 \over R} ) \, }{|X(y) -X(z + s h (z ) \, \nu (z))|} \, ds \\&= \int _{\Sigma ^n } \, \dfrac{ h(z ) \, d \sigma (z)\, }{|X(y) -X(z) |} \, + {\hat{N}}_1 [h] (y) \end{aligned}$$

with

$$\begin{aligned} {\hat{N}}_1 [0] (y) = (D_h {\hat{N}}_1)[0](y) = 0, \quad | {\hat{N}}_1 [h] (y) | \le C \Vert h \Vert _{L^\infty }^2 \end{aligned}$$

for some positive constant *C* uniformly bounded as $n \rightarrow \infty$. On the other hand, writing $\Sigma ^n = \cup _{k=0}^{n-1} \Sigma _k$ as in (1.6) and using the change of variable

$$\begin{aligned} z = z_0 + k T \, e_3\in \Sigma _k, \quad z_0 \in \Sigma _0, \quad e_3 = (0,0,1), \end{aligned}$$

we have

$$\begin{aligned}&\int _{\Sigma ^n } \, \dfrac{ h(z ) \, d \sigma (z) \, }{|X(y) -X(z) |} = \sum _{k=0}^{n-1} \int _{\Sigma _k } \, \dfrac{ h(z ) \, d \sigma (z)\, }{|X(y) -X(z) |} \\&\quad = \sum _{k=0}^{n-1} \int _{\Sigma _0 } \, \dfrac{ h(z_0 + kT \, e_3 ) \, d \sigma (z_0)\, }{|X(y) -X(z_0 + kT \, e_3 ) |} \, \\&\quad =\sum _{k=0}^{n-1} I_k (y), \quad {\text{ where }}\\&I_k (y) = \int _{\Sigma _0 } \, \ \dfrac{ h(z_0 ) \, d \sigma (z_0)\, }{\sqrt{|{\bar{y}} - {\bar{z}}_0|^2 + a_{Rk}^2 (1+{y_2 + z_{02} \over R} + {y_2 z_{02} \over R^2} )}} \end{aligned}$$

with

$$\begin{aligned} a_{Rk} = 2\, R \, \sin \left( {k T \over 2R } + {y_{3} - z_{03} \over 2\, R} \right) . \end{aligned}$$

To get this formula we use that *h* satisfies the symmetries (1.9) and we argue in a similar way as in the proof of Lemma 4.1, formula (4.8).

We follow the arguments to prove (4.12)–(4.13)– (4.14)–(4.15) in the proof of Lemma 4.1 to get

$$\begin{aligned} \int _{\Sigma ^n } \,&\dfrac{ h(z ) \, d \sigma (z) \, }{|X(y) -X(z) |} = \left( {2 \over T} \, \log n \, + b(y) \right) \, \int _{\Sigma _0 } \, \ h(z_0 ) \, d \sigma (z_0)\, + N_3 [h] (y) \end{aligned}$$

with *b* as in the statement of Lemma 4.2 and

$$\begin{aligned} N_3 [0] (y) = 0, \quad | N_3 [h] (y)| \le C \, \Vert h \Vert _{L^\infty } \end{aligned}$$

for some constant *C* uniformly bounded in *n*. Arguing as before, we can show that $N_3[h] (y)$ is even in $y_2$, if *h* is.

This gives the expansion in (4.6). We conclude with the remark that $y \rightarrow \ell _i [h] (y)$ in (4.6) satisfies (1.9), as consequence of Remark 4.1, Lemma 4.1 and the above argument. $\square$

## Invertibility of the Jacobi Operator

We combine the results of Propositions 3.1 and 4.1 to re-write Problem (1.16) in one to find $h: \Sigma \rightarrow {\mathbb {R}}$ and constants $\gamma$, $\lambda$ such that

5.1 $$\begin{aligned} \begin{aligned} J_{\Sigma ^n} [h]&= E_\gamma (y) + \, \gamma \, \left( {2 \over T} \, \log n \, + b(y) \, \right) \,\,\int _{\Sigma _0 } h + \, \gamma \, \ell _1 [h] (y) \\&\quad + n^{-1} \, \ell [h, D h, D^2 h] (y) + q[h, D h, D^2 h] (y) + \lambda \\&\quad {\text{ where }} \\ E_\gamma (y)&= \gamma \, F_0^n (y_{3} ) + \frac{\gamma }{n} F_1^n (y) \\&\quad + { y_2 \over n } [ F(y_3) - \gamma \, {\log n } \, \, {2\pi |\Omega _0 | \over T^2}], \\ F(y_3) =&\frac{2\pi }{T f} \Biggl ( { (2-(f')^2 ) \, f \, f'' \over (1+ (f')^2 )^{5\over 2}} +{1 + 3 (f')^2 \over (1+ (f')^2 )^{3\over 2}} \Biggl ), \end{aligned} \end{aligned}$$

Here $J_{\Sigma ^n}$ denotes the Jacobi operator of $\Sigma ^n$ defined by (3.9), $|\Omega _0|$ is the volume of the region enclosed by $\Sigma _0$, see (1.5) and *T* is the period of the Delaunay surface $\Sigma$. The functions $F_0^n$, $F_1^n$, *F* and *b* are smooth in their arguments and uniformly bounded together with their derivatives, as $n \rightarrow \infty$. They satisfy the symmetry assumptions (1.9). In addition, if *h* is even with respect to $y_2$, then the functions $\ell _1 [h] (y), \ q[h,Dh, D^2\,h ] (y)$ are even in $y_2.$ The function $E_\gamma$ in (5.1) satisfies the symmetries and periodicity conditions (1.9).

For $h \in C^{2,\alpha }$ satisfying (1.9), $\ell _1 [h]$, $\ell [h, Dh , D^2\,h]$ and $q [h, Dh , D^2\,h]$ satisfy (1.9). Besides, there exists a constant *C* such that

5.2 $$\begin{aligned} \begin{aligned}&\Vert \ell _1 [h] \Vert _{C^\alpha } + \Vert \ell [h, Dh, D^2 h] \Vert _{C^\alpha } \le C \Vert h\Vert _{C^{2, \alpha } }, \quad \\&\Vert q [h, Dh, D^2 h] \Vert _{C^\alpha } \le C \Vert h\Vert _{C^{2, \alpha } }^2. \end{aligned} \end{aligned}$$

The proofs of Propositions 3.1 and 4.1 yields uniform Lipschitz dependence on *h* of $\ell _1$, $\ell$ and *q* in (5.1). Precisely, there exists *C* such that

5.3 $$\begin{aligned} \begin{aligned} \Vert \ell _1 [h_1] - \ell _1 [h_2] \Vert _{C^\alpha }&\le C \Vert h_1 - h_2 \Vert _{C^{2, \alpha } } \\ \Vert \ell [h_1, Dh_1, D^2 h_1] - \ell [h_2, Dh_2, D^2 h_2] \Vert _{C^\alpha }&\le C \Vert h_1 - h_2 \Vert _{C^{2, \alpha } } \end{aligned} \end{aligned}$$

and

5.4 $$\begin{aligned} \begin{aligned}&\Vert q [h_1, Dh_1, D^2 h_1] - q [h_2, Dh_2, D^2 h_2] \Vert _{C^\alpha } \\&\quad \le C \left( \Vert h_1\Vert _{C^{2, \alpha } } + \Vert h_2\Vert _{C^{2, \alpha } } \right) \Vert h_1 - h_2 \Vert _{C^{2, \alpha } } \end{aligned} \end{aligned}$$

While all these terms are in principle defined only on $\Sigma ^n$, periodicity allows us to extend them to all $\Sigma$ naturally.

### Invertibility of the Jacobi operator

Let us consider a Delaunay surface $\Sigma$ with neck size $a\in (0, \frac{1}{2} )$ and period *T*, as introduced in section §1.1 and its Jacobi operator

$$\begin{aligned} J_\Sigma [h] = \Delta _\Sigma h + |A_\Sigma |^2 h \end{aligned}$$

defined for real functions $h \in C^{2,\alpha } (\Sigma )$ which are periodic in $y_3$. This subsection deals with solving a linear problem of the form

5.5 $$\begin{aligned} J_\Sigma [h] = E(y) {\quad \hbox {in } }\Sigma \end{aligned}$$

for right hand sides *E*(*y*) that are *T*-periodic in $y_3$ and even in the variables $y_3$ and $y_1$. So, we consider $E\in C^\alpha (\Sigma )$ that satisfies

5.6 $$\begin{aligned} \begin{aligned} E(y_1, y_2, y_3) =&E(-y_1, y_2, y_3 ), \\ E(y_1, y_2, y_3) =&E(y_1, y_2, -y_3 ), \\ E(y_1, y_2, y_3) =&E(y_1, y_2, y_3 +T). \end{aligned} \end{aligned}$$

To solve (5.5) in a suitable class of right-hand sides *E* satisfying (5.6), we need to identify the class of Jacobi fields that are *T*-periodic in $y_3$. The invariance of the mean curvature of $\Sigma$ under the rigid motions represented by translations along the 3 coordinate axes, gives that the 3 components of the normal vector are Jacobi fields, namely

$$\begin{aligned} J_\Sigma [ \nu _j] =0, \quad j=1,2,3. \end{aligned}$$

It is known that these functions actually span the space of all *T*-periodic in $y_3$ Jacobi fields. In other words,

#### Lemma 5.1

If $Z\in C^2 (\Sigma )$ is *T*-periodic in the variable $y_3$ then

$$\begin{aligned} J_\Sigma [Z]=0 \implies \exists \ \alpha _1, \alpha _2, \alpha _3 \in {\mathbb {R}}\mid \ Z= \sum _{j=1}^3 \alpha _j \nu _j. \end{aligned}$$

See for instance [6] Lemma 2.7, for a proof.

Rather than solving directly Problem (5.5) we consider the following projected version of it, as follows

5.7 $$\begin{aligned} \begin{aligned} J_\Sigma [h]&= E(y) -c\nu _2 - d {\quad \hbox {in } }\Sigma ,\\ \int _{\Sigma _0} h&= 0= \int _{\Sigma _0} h\nu _2. \end{aligned} \end{aligned}$$

#### Proposition 5.1

Let $\alpha \in (0,1)$ and let $E(y)\in C^\alpha (\Sigma )$ satisfy the symmetries (5.6), namely *T*-periodic in $y_3$ and even in the $y_3$ and $y_1$ variables. Then there exist scalars *c* and *d* given by (5.16) below and a unique solution *h* to problem (5.7) with the same symmetries. This *h* defines a linear operator of $h={\mathcal {T}} [E]$ with

$$\begin{aligned} \Vert {\mathcal {T}} (E)\Vert _{C^{2,\alpha } (\Sigma )} \le C \Vert E\Vert _{C^\alpha (\Sigma )}. \end{aligned}$$

For the proof, we consider first the more general problem

5.8 $$\begin{aligned} J_\Sigma [h] = E(y) {\quad \hbox {in } }\Sigma . \end{aligned}$$

The following result holds.

#### Lemma 5.2

Let $E \in C^\alpha (\Sigma )$ be *T*-periodic in $y_3$ and such that

5.9 $$\begin{aligned} \int _{\Sigma _0} E \nu _j = 0,\quad j=1,2,3,\end{aligned}$$

where $\nu _j$ are the 3 components of the normal vector to $\Sigma$. Then problem (5.8) has a unique solution *h*(*y*) which is *T*-periodic in $y_3$ and satisfies

5.10 $$\begin{aligned} \int _{\Sigma _0} h \nu _j = 0,\quad j=1,2,3. \end{aligned}$$

*h* defines a linear operator $h = {\mathcal {H}} (E)$ that satisfies an the estimate of the form

5.11 $$\begin{aligned} \Vert {\mathcal {H}} (E)\Vert _{C^{2,\alpha }(\Sigma ) } \le C \Vert E \Vert _{C^{\alpha }(\Sigma ) }. \end{aligned}$$

#### Proof

Let us consider the functions

$$\begin{aligned} \xi _j= (1- \Delta _{\Sigma } ) \nu _j = (1+|A_\Sigma |^2) \nu _j, \quad j=1,2,3 \end{aligned}$$

and consider the operator

$$\begin{aligned} {\mathcal {A}}(h) = (1- \Delta _\Sigma )^{-1} (h + |A_\Sigma |^2h), \quad \end{aligned}$$

defined on the space *M* of functions $h\in C^\alpha (\Sigma )$ that are *T*-periodic in $y_3$ such that

5.12 $$\begin{aligned} \int _{\Sigma _0} \xi _j h = 0, \quad j=1,2,3.\end{aligned}$$

${\mathcal {A}}$ is a compact operator in the Banach space *M* endowed with its natural norm. ${\mathcal {A}}$ applies the space *M* into itself. Indeed, for $h\in M$ we have

$$\begin{aligned} \begin{aligned} \int _{\Sigma _0} {\mathcal {A}}(h)\xi _j =&\int _{\Sigma _0} (1- \Delta _\Sigma )^{-1} (h + |A_\Sigma |^2h) (1- \Delta _\Sigma ) \nu _j \\ =&\int _{\Sigma _0} (h + |A_\Sigma |^2h)\nu _j = \int _{\Sigma _0} h\xi _j =0. \end{aligned} \end{aligned}$$

Now, if $h\in \ker (I - {\mathcal {A}})$ then $h\in C^{2,\alpha }(\Sigma )$ solves $J_\Sigma [h] =0$, hence from Lemma 5.1, *h* is a linear combination of the functions $\nu _j$. Conditions (5.12) then imply $h=0$. Now, we observe that Equation (5.8) can be written as

$$\begin{aligned} (I- {\mathcal {A}})(h) = G, \quad G= (\Delta _\Sigma - 1)^{-1}(E). \end{aligned}$$

We observe that $G\in M$. Indeed,

$$\begin{aligned} \begin{aligned} \int _{\Sigma _0} G\xi _j =&\int _{\Sigma _0} (\Delta _\Sigma - 1)^{-1}(E) (\Delta _\Sigma - 1)\nu _j\\ =&\int _{\Sigma _0} E\nu _j = 0, \quad j=1,2,3. \end{aligned} \end{aligned}$$

Then, Fredholm’s alternative yields a unique solution $h\in M$ to this problem and hence of (5.8). That solution defines a linear operator of *E* that satisfies a bound of the form (5.11) from elliptic estimates. Finally, we modify *h* in the form $h_* = h +\sum _{i=1}^3 \alpha _i \nu _j$ where $\alpha _i$ solves the linear system

$$\begin{aligned} \sum _{i=1}^3 \alpha _i \int _{\Sigma _0} \nu _i \nu _j = - \int _{\Sigma _0} h\nu _j, \quad j=1,2,3. \end{aligned}$$

Then $h_*$ still solves (5.8) with conditions (5.10) and satisfies an estimate of the form (5.11). The proof is concluded. $\square$

Next, we will solve the equation

5.13 $$\begin{aligned} J_\Sigma [ {\bar{h}} ] = 1 {\quad \hbox {in } }\Sigma . \end{aligned}$$

By odd symmetries of the functions $\nu _j$, the function $E=1$ satisfies the orthogonality conditions (5.9) and the solution ${\bar{h}}$ of (5.13) predicted by Lemma 5.2 exists and is unique. We need to describe this function in a more explicit form. $\square$

#### Lemma 5.3

The unique solution of Problem (5.13) is *T*-periodic and even in $y_3$ and satisfies

$$\begin{aligned} \int _{\Sigma _0} {\bar{h}} > 0 \end{aligned}$$

#### Proof

Using the notation in [6], Lemma 2.6, see also [5, 19, 24], the Jacobi operator in $\Sigma$ can be expressed in the simple form

$$\begin{aligned} J_\Sigma [h] = x(t)^{-2} (h_{\theta \theta } + h_{tt} + p(t)h ) \end{aligned}$$

Here the parametrization of the Delaunay surface in isothermal coordinates,

$$\begin{aligned} y(t,\theta ) = (x(t) \cos \theta , x(t) \sin \theta , z(t) ), \end{aligned}$$

where *z*(*t*) is strictly increasing and *x*(*t*) is positive, even and periodic with a period $2\tau >0$. We assume $x(-\tau ) = x(\tau ) = a, \quad x(0) = 1-a$, so that also $z(\pm \tau ) = \pm \frac{T}{2}$. See [6] for the precise definitions. Of course we have $x(t) = f(z(t))$. *p*(*t*) is an explicit even function. We look for a solution to Problem (5.13) which is a function only of *t*, $h= {\bar{h}}(t)$. The equation on a periodic cell $(-\frac{\tau }{2}, \frac{\tau }{2})$ then reads

5.14 $$\begin{aligned} h'' + p(t) h = x(t)^2 {\quad \hbox {in } }(- \tau , \tau ) \end{aligned}$$

and we are looking for an even solution. In coordinates $(\theta , t)$ we see that $\nu _3$ does not depend on $\theta$, more explicitly, $\nu _3(t) = - \frac{f'(z(t)) }{ \sqrt{1+ f'(z(t) )^2 }}$. We see that $\nu _3(t)$ is an odd function, positive on $(0, \tau )$. This function annihilates the Jacobi operator, and hence the linear operator in (5.14). We look for a solution in the form $h(t) = \nu _3(t)\omega (t)$. Substituting we obtain

$$\begin{aligned} \omega (\nu _3'' + p(t) \nu _3) + \nu _3\omega '' + 2\omega ' \nu _3' = x(t)^2. \end{aligned}$$

Recalling that $\nu _3'' + p(t) \nu _3=0$ and multiplying the equation by $\nu _3$ we get

$$\begin{aligned} (\nu _3^2(t) \omega '(t) )' = x(t)^2 \nu _3(t), \end{aligned}$$

from where we deduce that a solution to (5.14) in $(0,\tau )$ is given by

$$\begin{aligned} {\bar{h}}(t) = \nu _3(t) \int _0^t \frac{dz}{\nu _3^2(z)} \int _0^z x(s)^2 \nu _3(s) ds. \end{aligned}$$

This function is regular at $t=0$ and extends as an even solution to (5.14) to the entire interval $(-\tau , \tau )$ and to an even $2\tau$-periodic non-negative solution for all *t*. Hence ${\bar{h}}$ solves Problem (5.13) in the entire $\Sigma$. *h* is positive except at $t=0$. In particular, we have $\int _{\Sigma _0} {\bar{h}} > 0$, as desired. $\square$

### Proof of Proposition 5.1

We begin by identifying the unique candidates for the constants *c* and *d*. Testing Equation (5.7) against $\nu _2$ in the periodic cell $\Sigma _0$ we get

$$\begin{aligned} \begin{aligned} \int _{\Sigma _0 } J_\Sigma [h] \nu _2 = \int _{\Sigma _0} h J_\Sigma [ \nu _2] = 0&= \int _{\Sigma _0} E \nu _2 - c\int _{\Sigma _0} \nu _2^2 - d\int _{\Sigma _0} \nu _2. \end{aligned} \end{aligned}$$

Testing against ${\bar{h}}$ in Lemma 5.3, we get

5.15 $$\begin{aligned} \begin{aligned} \int _{\Sigma _0 } J_\Sigma [h] {\bar{h}} = \int _{\Sigma _0} h J_\Sigma [ {\bar{h}}] = \int _{\Sigma _0} h=0&= \int _{\Sigma _0} E {\bar{h}} - c\int _{\Sigma _0} {\bar{h}} \nu _2 - d \int _{\Sigma _0} {\bar{h}}. \end{aligned} \nonumber \\ \end{aligned}$$

Since $\int _{\Sigma _0} {\bar{h}} \nu _2 =0 = \int _{\Sigma _0} \nu _2$ and $\int _{\Sigma _0} {\bar{h}} >0$ we can explicitly solve for the coefficients *c* and *d*

5.16 $$\begin{aligned} d= \frac{ \int _{\Sigma _0} E {\bar{h}} }{\int _{\Sigma _0} {\bar{h}} }, \quad c= \frac{ \int _{\Sigma _0} E \nu _2 }{\int _{\Sigma _0} \nu _2^2 }. \end{aligned}$$

We observe that the choices of *c* and *d* as in (5.16) yield that the function ${\bar{E}} = E - c\nu _2 -d$ satisfies $\int _{\Sigma _0} {\bar{E}} \nu _2= 0$. The even symmetries (5.6) in *E* and the odd ones for $\nu _1$ and $\nu _3$ yield

$$\begin{aligned} \int _{\Sigma _0} {\bar{E}} \nu _j= 0, \quad j=1,2,3. \end{aligned}$$

Thus, with these choices of *d* and *c*, Lemma 5.2 yields the existence of a unique solution of (5.7) *T*-periodic in $y_3$ that satisfies conditions (5.10). Testing the equation once more against ${\bar{h}}$, the computation (5.15) now yields $\int _{\Sigma _0} h = 0.$ Uniqueness implies evenness in the variable $y_3$. Hence with the choices (5.16) *h* is *T*-periodic and even in $y_3$ and solves

$$\begin{aligned} \left\{ \begin{aligned} J_\Sigma [h]&= E(y) -c\nu _2 - d {\quad \hbox {in } }\Sigma ,\\ \int _{\Sigma _0} h&= 0= \int _{\Sigma _0} h\nu _2. \end{aligned}\right. \end{aligned}$$

The elliptic estimate (5.11) completes the proof. $\square$

## Resolution of the Full Problem

We recall that to solve Problem (1.16) we just need to find a function $h: \Sigma \rightarrow {\mathbb {R}}$ and constants $\gamma$, $\lambda$ such that equation (5.1) is satisfied, namely

$$\begin{aligned} \begin{aligned} J_{\Sigma ^n} [h]&= E_\gamma (y) + \, \gamma \, \left( {2 \over T} \, \log n \, + b(y) \, \right) \,\,\int _{\Sigma _0 } h + \, \gamma \, \ell _1 [h] (y) \\&\quad + n^{-1} \, \ell [h, D h, D^2 h] (y) + q[h, D h, D^2 h] (y) + \lambda , \end{aligned} \end{aligned}$$

where

$$\begin{aligned} \begin{aligned} E_\gamma (y)&= \gamma \, F_0^n (y_{3} ) + \frac{\gamma }{n} F_1^n (y) + { y_2 \over n } [ F(y_3) - \gamma \, {\log n } \, \, {2\pi |\Omega _0 | \over T^2}]. \end{aligned} \end{aligned}$$

$F(y_3)$ and the rest of the terms are defined in formula (5.1).

Instead of solving the problem directly, we formulate the projected problem

6.1 $$\begin{aligned} \begin{aligned} J_{\Sigma ^n} [h]&= E_\gamma (y) + \, \gamma \, \left( {2 \over T} \, \log n \, + b(y) \, \right) \,\,\int _{\Sigma _0 } h + \, \gamma \, \ell _1 [h] (y) \\&\quad + n^{-1} \, \ell [h, D h, D^2 h] (y) + q[h, D h, D^2 h] (y) - d - c\nu _2 \end{aligned} \end{aligned}$$

and we look for a solution *h* that satisfies $\int _{\Sigma _0} h = 0$ and we additionally require $c=0$.

We formulate this problem using the operator ${\mathcal {T}}$ in Proposition 5.1 in the fixed point form

6.2 $$\begin{aligned} \begin{aligned} h&= {{\mathcal {E}} } (h, \gamma ) \\ {{\mathcal {E}} } (h, \gamma )&:={\mathcal {T}} ( E_\gamma + \, \gamma \, \ell _1 [h] + n^{-1} \, \ell [h, D h, D^2 h] + q[h, D h, D^2 h] ) \end{aligned} \end{aligned}$$

coupled with the relation $c=0$ which becomes

6.3 $$\begin{aligned} c(h,\gamma )= &   \frac{1}{ \int _\Sigma \nu _2^2 } \int _\Sigma (\, E_\gamma + \, \gamma \, \ell _1 [h] \nonumber \\  &   + n^{-1} \, \ell [h, D h, D^2 h] + q[h, D h, D^2 h] ) \nu _2 \ =\ 0. \end{aligned}$$

The constant *d* in (6.1) is given by

$$\begin{aligned} d= \frac{1}{ \int _\Sigma {\bar{h}} } \int _\Sigma (\, E_\gamma + \gamma \, \ell _1[h] + n^{-1} \, \ell (h, D h, D^2 h) + q(h, D h, D^2 h) ) {\bar{h}} \end{aligned}$$

where ${\bar{h}}$ is given by Lemma 5.3.

Our problem is reduced to finding a solution $(h, \gamma )$ in system (6.2)–(6.3). The principle is simple. We look for a solution *h* whose size is of the order of that of $E_\gamma$ which turns out to be $O(\gamma )$. The contribution of the terms *h*-dependent of the right-hand side will then be $o(\gamma )$. It is convenient to solve first a "piece" of equation (6.2),

$$\begin{aligned} h_0 = {\mathcal {T}} ( \gamma F_0^n (y_3) + \gamma \, \ell _1[h_0] + q(h_0,Dh_0,D^2 h_0)). \end{aligned}$$

This problem can be embedded in the space of functions *h* that in addition to the symmetry and periodicity conditions (5.6) are even in the $y_2$-variable: $h(y_1,-y_2,y_3)= h(y_1,y_2,y_3)$. Thanks to (5.2)–(5.3)–(5.4), we apply the contraction mapping principle which yields that this problem has a unique small solution $h_0$, with

$$\begin{aligned} \int _{\Sigma _0} h_0 =0, \quad {\text{ and }} \quad \Vert h_0 \Vert _{C^{2, \alpha } (\Sigma _0)} \le {C \over \log n}. \end{aligned}$$

The reason being that the terms $\gamma F_0^n (y_3) + \gamma \, \ell _1[h] + q(h,Dh,D^2\,h))$ satisfy conditions (5.6) and evenness in $y_2$ if *h* does.

Now let us decompose $h=h_0+ h_1$. The problem for $h_1$ then becomes

6.4 $$\begin{aligned} \begin{aligned} h_1=&{\mathcal {T}} \Big ( E_\gamma ^1 + \vartheta _1(h_1) + \vartheta _2(h_1) \Big ) \end{aligned} \end{aligned}$$

where

$$\begin{aligned} \begin{aligned} E^1_\gamma (y) =&\frac{\gamma }{n} F_1^n (y) + { y_2 \over n } [ F(y_3) - \gamma \, {\log n } \, \, {2\pi |\Omega _0 | \over T^2}], \\ \vartheta _1 (h_1)&= n^{-1} \, \ell (h_0+h_1, D (h_0+h_1), D^2 (h_0 + h_1)) \\ \vartheta _2 (h_1)&= \gamma \ell _1 [h_0+ h_1] - \gamma \ell _1 [h_0] \\&\quad + q(h_0+h_1,D(h_0 + h_1),D^2 (h_0 + h_1))\, - \, q(h_0,Dh_0,D^2 h_0). \end{aligned} \end{aligned}$$

The following properties can be immediately checked

$$\begin{aligned} \begin{aligned} \Vert E_\gamma ^1\Vert _{C^\alpha (\Sigma _0)} \le&\, \frac{C}{n}, \\ \Vert \vartheta _1 (h_1)\Vert _{C^\alpha (\Sigma _0)} \le&\, \frac{C}{n} \bigg (\gamma + \Vert h_1\Vert _{C^{2,\alpha } (\Sigma _0)} \bigg ), \\ \Vert \vartheta _2 (h_1)\Vert _{C^\alpha (\Sigma _0)} \le&\, C\bigg [ \gamma \Vert h_1\Vert _{C^{2,\alpha }} + \Vert h_1\Vert ^2_{C^{2,\alpha } (\Sigma _0)} \bigg ] \end{aligned} \end{aligned}$$

(see (5.2), (5.3), (5.4)). Let us consider the equation (6.4) for $h_1$ in the region of functions $h_1$ with $\int _{\Sigma _0} h_1 =0$ and $\Vert h_1\Vert _{C^2_\alpha (\Sigma _0)} \le \frac{D}{n}$ for some suitably large constant *D*. We check that the operator in the right-hand side of (6.4) applies this region into itself, and in addition, it is a contraction mapping. We conclude that (6.4) has a unique solution with

$$\begin{aligned} \int _{\Sigma _0} h_1 =0, \quad {\text{ and }} \quad \Vert h_1 \Vert _{C^{2, \alpha } (\Sigma _0)} \le {C \over n}. \end{aligned}$$

We now have $h= h_0 + h_1$ defines an operator $h= h(\gamma )$. Substituting this into the equation (6.3) we have reduced the full problem to

6.5 $$\begin{aligned} \int _{\Sigma _0} ( E^1_\gamma + \vartheta _1(h_1) + \vartheta _2(h_1))\nu _2 = 0. \end{aligned}$$

Here we have used that because of the even symmetry in $y_2$ we have that

$$\begin{aligned} \int _{\Sigma _0} (\gamma F_0^n (y_3) + \gamma \, \ell _1[h_0] + n^{-1} \, \ell (h_0, D h_0, D^2 h_0) + q(h_0,Dh_0,D^2 h_0)) \nu _2 = 0. \end{aligned}$$

The relation (6.3) will be at main order the equation

$$\begin{aligned} \int _{\Sigma _0} E_\gamma ^1 \nu _2 = 0. \end{aligned}$$

Let us compute the left-hand side of this equation. We will denote by $c_1,c_2,...$ different positive constants arising in the computation.

Using the parametrization of the Delaunay surface introduced in §3, we have $\nu _2 = {\sin \theta \over \sqrt{1+ (f')^2}}$ and $d\sigma = \sqrt{{\det } g} \, {\text {d}}y_3 \, {\text {d}}\theta$ (see (3.28)) and hence

$$\begin{aligned} c_1:= \int _{\Sigma _0} \nu _2^2 = (\int _0^{2\pi } \sin ^2 \theta \, {\text {d}} \theta ) \int _{-{T \over 2}}^{T\over 2} {f \over \sqrt{1+ (f')^2 } } \, {\text {d}}y_3. \end{aligned}$$

Using Propositions 3.1 and 4.1 we find

$$\begin{aligned} \int _{\Sigma _0} E_\gamma ^1 \, \nu _2&= {1\over R} \int _0^{2\pi } \int _{-{T \over 2}}^{T\over 2} f \sin ^2 \theta \, \Biggl ( { (2-(f')^2 ) \, f \, f'' \over (1+ (f')^2 )^{5\over 2}} +{1 + 3 (f')^2 \over (1+ (f')^2 )^{3\over 2}} \Biggl ) \, {\text {d}}y_3 \, d\theta \\&\quad -\gamma \, {\log n \over n} \, {2\pi |\Omega _0| \over T^2} \int _0^{2\pi } \int _{-{T \over 2}}^{T\over 2} f^2 \sin ^2 \theta \, d y_3 \, d\theta + O \left( {\gamma \over n}\right) \\&= {2\pi \over nT} \, \left( \int _0^{2\pi } \sin ^2 \theta \, d \theta \right) \, \left( 2 \, I_a - \gamma \, \log n \, {|\Omega _0 |\over T} + O(\gamma ) \right) \end{aligned}$$

where $I_a$ is the integral defined in (2.2). Setting

$$\begin{aligned} \begin{aligned} c_2 =&{2\pi \over T} \, \left( \int _0^{2\pi } \sin ^2 \theta \, d \theta \right) \, \\ c_3 =&{|\Omega _0 |\over T},\\ c_4 =&{2\pi \over T} \, \left( \int _{-{T \over 2}}^{T\over 2} {f \over \sqrt{1+ (f')^2 } } \, {\text {d}}y_3 \right) ^{-1} \, \end{aligned} \end{aligned}$$

we find

$$\begin{aligned} \begin{aligned} { \int _{\Sigma _0} E_\gamma ^1 \, \nu _2 \over \int _{\Sigma _0} \nu _2^2 }&= \frac{c_4}{n} \left( 2 \, I_a - c_3 \gamma \, \log n \, + O(\gamma ) \right) \end{aligned} \end{aligned}$$

and hence at main order

$$\begin{aligned} \gamma \approx \gamma _n:= \frac{c_5}{\log n }, \quad c_5= \frac{2}{c_3} I_a. \end{aligned}$$

The value of $\gamma _n$ will indeed be a positive number if $I_a>0$, which is precisely what we are assuming. Now, we assume that we are working in a region of positive scalars $\gamma$ with

$$\begin{aligned} |\gamma -\gamma _n| \le \frac{M}{\log ^2 n} \end{aligned}$$

for a fixed and adequately large number *M*.

Then the full problem is reduced, after substitution into (6.5) to finding $\gamma$ such that a relation of the following form holds

$$\begin{aligned} \int _{\Sigma _0} \left( E_\gamma ^1 + O\left( \frac{\gamma }{n}\right) \right) \nu _2 = c_2 (2 I_a- c_3 \gamma \log n + o(1) ) \end{aligned}$$

where the smaller order terms satisfy also small Lipschitz conditions. The choice of $\gamma$ such that this quantity equals zero immediately follows. The continuity on *a* of the parameter $\gamma$ and the function *h* in uniform norms follows from the fixed point characterization in a standard manner. The proof is concluded. $\square$

## Data Availability

Data sharing not applicable to this article as no datasets were generated or analysed during the current study.

## Appendix

### Proof of Lemma 2.1

It is straightforward to check that $I_a >0$ for any ${1\over 4} \le a < {1\over 2}$. Since *f* satisfies (1.4), one can show that the following quantity is conserved:

7.1 $$\begin{aligned} f^2 - \frac{f}{ \sqrt{1+ f'(s)^2}} = - a(1-a). \end{aligned}$$

An algebraic manipulation using (1.4) and (7.1) gives that

$$\begin{aligned} \begin{aligned} I_a&= \int _0^{T\over 2} {f_* (s) \over 1+ (f'(s))^2} \, ds, \quad {\text{ where }} \\ f_*(t)&= f^2 \, (-1 + 4 (f')^2 ) + \, a \, (1-a) \, (3 + 2 (f')^2). \end{aligned} \end{aligned}$$

Since $f(s)^2 \le (1-a)^2$ for all $s \in [0,{T\over 2}]$ and $a \in [{1\over 4}, {1\over 2} ]$, we readily get

$$\begin{aligned} f_*(s) > - f(s)^2 + 3a (1-a) \ge 0. \quad \end{aligned}$$

Let us now consider *a* close to 0.

Let us consider the change of variable in the integral (2.2) given by

$$\begin{aligned} s= z(t). \end{aligned}$$

Using the notation in [5, 6], $s=z(t)$ is the unique diffeomorphism such that $z(0)=0$, $z' >0$ and

$$\begin{aligned} y(t,\theta ) = (x(t) \cos \theta , x(t) \sin \theta , z(t) ), \quad x(t)= f(z(t)) \end{aligned}$$

is a conformal parametrization of the unduloid $\Sigma$, i.e.

$$\begin{aligned} {\partial } _t y \cdot {\partial } _\theta y = 0= |{\partial } _t y|^2 - |{\partial } _\theta y |^2. \end{aligned}$$

This relation is equivalent to

7.2 $$\begin{aligned} x^2= (x')^2 + (z')^2. \end{aligned}$$

The functions $x=x(t)$ and $z=z(t)$ satisfy

7.3 $$\begin{aligned} \begin{aligned} x''&= (1-2 a (1-a)) x - 2 x, \quad x(0)=1-a, \quad x'(0)=0 \\ z'&= a (1-a) + x^2, \quad z(0) =0. \end{aligned} \end{aligned}$$

The function *x* is $2\tau$-periodic, $x (t+2\tau ) = x(t)$ for all *t*, $x(-\tau ) = x(\tau ) = a$ and $z(\pm \tau ) = \pm \frac{T}{2}$. We refer to [5, 6, 24] for the derivation of these facts.

Using (7.2), we compute

$$\begin{aligned} {\text {d}}s = z'(t) {\text {d}}t, \quad {{\text {d}}f \over {\text {d}}s} = {z' \over x'}, \quad 1+ ({{\text {d}}f \over {\text {d}}s})^2 = {x^2 + \left( a (1-a) + x^2 \right) ^2} \end{aligned}$$

and hence

7.4 $$\begin{aligned} \begin{aligned} I_a&= \int _0^{\tau } A_a (t) B_a (t) \, {\text {d}}t \\ A_a (t)&= a (1-a) + x^2 \\ B_a (t)&= - z' + 4 (x')^2 + {a (1-a) \over x^2} \, ( 3 (z')^2 + 2 (x')^2 ) \end{aligned} \end{aligned}$$

We claim that there exists $C>0$ such that for all $a >0$ small enough

7.5 $$\begin{aligned} x(t) = \textrm{sech } (t)\, \left( 1 + a (-1 + t \tanh t) \right) + a^2\, \varphi _a (t) \end{aligned}$$

where

7.6 $$\begin{aligned} \Vert \varphi _a \Vert _{C^2_{\textrm{loc}} ({\mathbb {R}})} \le C. \end{aligned}$$

For the moment we assume the validity of (7.5)–(7.6) and proceed with our argument to prove that $I_a >0$ for all $a>0$ small. Using the notation introduced in (7.4), we differentiate $I_a$ with respect to *a*

$$\begin{aligned} \partial _a I_a = \int _0^\tau \left( \partial _a A_a \, B_a + A_a \, \partial _a B_a \right) \, {\text {d}}t + (A_a B_a) (\tau ) \, \left( \partial _a \tau \right) . \end{aligned}$$

In Lemma 2.2 in [5] it is proved that $\tau = -2 \log a + {\tilde{\tau }} (a)$ with ${\tilde{\tau }} (a)$ a smooth function which is uniformly bounded with its derivative as $a \rightarrow 0$. This fact, in combination with (7.5)–(7.6), give that

$$\begin{aligned} (A_a B_a) (\tau ) \, \left( \partial _a \tau \right) = O(a) \quad {\text{ as }} \quad a \rightarrow 0. \end{aligned}$$

Let

7.7 $$\begin{aligned} x_0 (t) = \text {sech}t, \quad \varphi (t) = \text {sech}t \, (-1 + t \tanh t). \end{aligned}$$

Using (7.2) and (7.3), we get

$$\begin{aligned} ({\partial } _a A_a )_{a=0}&= 1+ 2 x_0 \varphi \\ ({\partial } _a B_a )_{a=0}&=- 9 x_0^2 +2 + 8 x_0 \varphi -20 x_0^3 \varphi . \end{aligned}$$

Since $$I_0=0$$, $$A_0= x_0^2$$ and $$B_0 = 4 x_0^2 -5 x_0^4 $$, Taylor expanding $$I_a$$ in (7.4) we obtain

$$\begin{aligned} I_a&= a \, \int _0^{\infty } \left( 1+ 2 x_0 \varphi \right) \left( 4 x_0^2 -5 x_0^4 \right) + x_0^2 \left( - 9 x_0^2 +2 + 8 x_0 \varphi -20 x_0^3 \varphi \right) \, {\text {d}}t \\&\quad + O(a^2) \quad {\text{ as }} \quad a \rightarrow 0. \end{aligned}$$

We use that

$$\begin{aligned} \int \text {sech}^2 (t) \, {\text {d}}t&= \tanh t + c, \quad \\ \int \text {sech}^4 (t) \, {\text {d}}t&= {1\over 3} \, (2 + \cosh (2 t)) \, \text {sech}^2(t) \, \tanh (t)+c , \quad \\ \int \text {sech}^6 (t) \, {\text {d}}t&={1\over 15} \left( 8 + 6 \cosh (2t) + \cosh (4t) \right) ) \, \text {sech}^4 (t) \, \tanh (t) +c \\ \int \text {sech}^4 (t) \, t \, \tanh t \, {\text {d}}t&= {1\over 48} (-12 t + 4 \sinh (2 t) + \sinh (4 t)) \text {sech}^4(t) + c\\ \int \text {sech}^6 (t) \, t \, \tanh t \, {\text {d}}t&= {1\over 360} (-60 t + 15 \sinh (2 t) + 6 \sinh (4 t)\\  &\quad + \sinh (6 t)) \, \text {sech}^6 (t) + c \end{aligned}$$

to get

$$\begin{aligned} \int _0^\infty x_0^2 (t) \, {\text {d}}t&=1, \quad \int _0^\infty x_0^4 (t) \, {\text {d}}t = {2\over 3} , \quad \int _0^\infty x_0^6 (t) \, {\text {d}}t = {8 \over 15}\\ \int _0^\infty x_0^4 (t) \, t \, \tanh t \, {\text {d}}t&= {1\over 6}, \quad \int _0^\infty x_0^6 (t) \, t \, \tanh t \, {\text {d}}t = {4 \over 45}. \end{aligned}$$

This gives

$$\begin{aligned}&\int _0^{\infty } \left( 1+ 2 x_0 \varphi \right) \left( 4 x_0^2 -5 x_0^4 \right) + x_0^2 \left( - 9 x_0^2 +2 + 8 x_0 \varphi -20 x_0^3 \varphi \right) \, {\text {d}}t\\&\quad = \int _0^\infty \left( -30 \, x_0^4 + 6 \, x_0^2 + 30 \, x_0^6 + 16 \, x_0^4 \, t \, \tanh t - 30 \, x_0^6 \, t \,\tanh t \right) \, {\text {d}}t \\&\quad = 2 >0 \end{aligned}$$

and hence $I_a >0$ for all $a>0$ small.

We now prove (7.5)–(7.6). Observe that the function $x_0$ in (7.7) solves

$$\begin{aligned} x''= x - 2 x^3, \quad {\text{ in }} \quad {\mathbb {R}}, \quad x (0)=1, \quad x'(0)=0. \end{aligned}$$

The proof of (7.5)–(7.6) is based on a formal linearization of (7.3) in *a*, at $a=0$. Hence we consider the linearized operator around $x_0$

$$\begin{aligned} L_0 (\varphi ) = \varphi '' - \varphi + 6 x_0^2 \varphi . \end{aligned}$$

A direct computation gives that the function $\varphi$ defined in (7.7) solves

$$\begin{aligned} L_0 (\varphi ) =-2 x_0, \quad \varphi (0)=-1, \quad \varphi ' (0)=0. \end{aligned}$$

Replacing the expression for *x* in (7.5) in (7.3), we get that $\varphi _a$ solves

7.8 $$\begin{aligned} \begin{aligned} L_0 (\varphi _a)&=2 a^2 x_0 - 2a^2 (1-a) \varphi - 6 x_0 a^2 \varphi ^2 - 2 a^3 \varphi ^3 + N (\varphi _a) \\ \varphi _a (0)&=0, \quad \varphi _a' (0) = 0 \end{aligned} \end{aligned}$$

where

$$\begin{aligned} N(\varphi _a)= 2 a (1-a) \varphi _a - 6x_0 [ (a \varphi + \varphi _a)^2 - a^2 \varphi ^2] -2 [ (a\varphi + \varphi _a)^3 - a^3 \varphi ^3]. \end{aligned}$$

The function $x_0' (t) = \tanh (t) \, \text {sech}(t)$ solves $L_0 (x_0' ) =0$. Using the variation of parameter formula we check that, for a bounded function *h*,

7.9 $$\begin{aligned} \psi (t) = \Theta (h) (t):= x_0' (t) \int _0^t {ds \over (x_0')^2 (s) } \int _0^s h(\eta ) x_0' (\eta ) \, d\eta \end{aligned}$$

is the only solution to

$$\begin{aligned} L_0 (\psi ) = h, \quad \psi (0) =0, \quad \psi ' (0)=0. \end{aligned}$$

Besides there exists a constant $C>0$ such that

$$\begin{aligned} \Vert \Theta (h) \Vert _{C^{2, \alpha }_{\textrm{loc}} ({\mathbb {R}})} \le C \Vert h \Vert _{C^{0, \alpha }_{\textrm{loc}} ({\mathbb {R}})}. \end{aligned}$$

In terms of the operator $\Theta$ the solution $\varphi _a$ to (7.8) is a fixed point for the problem

$$\begin{aligned} \varphi _a = {{\mathcal {A}}} (\varphi _a):= \Theta \left( 2 a^2 x_0 - 2a^2 (1-a) \varphi - 6 x_0 a^2 \varphi ^2 - 2 a^3 \varphi ^3 + N (\varphi _a) \right) . \end{aligned}$$

Using Contraction Mapping, we show that the above problem has a unique fixed point in the set

$$\begin{aligned} {{\mathcal {B}}} = \{ \psi \in C^{2, \alpha }_{\textrm{loc}} ({\mathbb {R}}) \,: \, \Vert \psi \Vert _{C^{2, \alpha }_{\textrm{loc}} ({\mathbb {R}})} \le r a^2\}. \end{aligned}$$

For any $\psi \in {{\mathcal {B}}}$, a direct use of the explicit formula (7.9) gives

$$\begin{aligned} \Vert {{\mathcal {A}}} (\psi ) \Vert _{C^{2, \alpha }_{\textrm{loc}} ({\mathbb {R}})}&\le C \Vert 2 a^2 x_0 - 2a^2 (1-a) \varphi - 6 x_0 a^2 \varphi ^2 - 2 a^3 \varphi ^3 + N (\psi ) \Vert _{C^{0, \alpha }_{\textrm{loc}} ({\mathbb {R}})} \\&\le C \Vert 2 a^2 x_0 - 2a^2 (1-a) \varphi - 6 x_0 a^2 \varphi ^2 - 2 a^3 \varphi ^3 \Vert _{C^{0, \alpha }_{\textrm{loc}} ({\mathbb {R}})} \\&\quad + C \Vert N (\psi ) \Vert _{C^{0, \alpha }_{\textrm{loc}} ({\mathbb {R}})} \le c_1 a^2, \end{aligned}$$

for some $c_1$. Taking $r>c_1$, this estimate shows that the map ${{\mathcal {A}}}$ send ${{\mathcal {B}}}$ into itself.

Besides, for any $\psi _1$, $\psi _2 \in {{\mathcal {B}}}$, we see that

$$\begin{aligned} \Vert {{\mathcal {A}}} (\psi _1 ) - {{\mathcal {A}}} (\psi _2 ) \Vert _{C^{2, \alpha }_{\textrm{loc}} ({\mathbb {R}})}&\le C \Vert N (\psi _1 ) - N(\psi _2) \Vert _{C^{0, \alpha }_{\textrm{loc}} ({\mathbb {R}})} \\&\le C a (1-a) \Vert \psi _1 - \psi _2 \Vert _{C^{0, \alpha }_{\textrm{loc}} ({\mathbb {R}})} \\&\quad + C \Vert x_0 a \varphi (\psi _1 - \psi _2) \Vert _{C^{0, \alpha }_{\textrm{loc}} ({\mathbb {R}})} + C \Vert \psi _1^2 - \psi _2^2 \Vert _{C^{0, \alpha }_{\textrm{loc}} ({\mathbb {R}})} \\&\le C a \Vert \psi _1 - \psi _2 \Vert _{C^{0, \alpha }_{\textrm{loc}} ({\mathbb {R}})} \\&\quad + C \Vert \psi _1^2 + \psi _2^2 \Vert _{C^{0, \alpha }_{\textrm{loc}} ({\mathbb {R}})} \Vert \psi _1 - \psi _2 \Vert _{C^{0, \alpha }_{\textrm{loc}} ({\mathbb {R}})} \end{aligned}$$

for a constant *C* independent of *a*, whose value changes from line to line, and within the same line. This gives that for any sufficiently $a>0$, ${{\mathcal {A}}}$ defines a contraction mapping of the set ${{\mathcal {B}}}$. This concludes the proof of (7.5)–(7.6). $\square$
